# Fuzzy identification of bioactive components for different efficacies of rhubarb by the back propagation neural network association analysis of UPLC-Q-TOF/MS^E^ and integrated effects

**DOI:** 10.1186/s13020-022-00612-9

**Published:** 2022-04-26

**Authors:** Jia-Qian Chen, Yan-Yan Chen, Xia Du, Hui-Juan Tao, Zong-Jin Pu, Xu-Qin Shi, Shi-Jun Yue, Gui-Sheng Zhou, Er-Xin Shang, Yu-Ping Tang, Jin-Ao Duan

**Affiliations:** 1 Key Laboratory of Shaanxi Administration of Traditional Chinese Medicine for TCM Compatibility, State Key Laboratory of Research & Development of Characteristic Qin Medicine Resources (Cultivation), Shaanxi Key Laboratory of Chinese Medicine Fundamentals and New Drugs Research, Shaanxi Collaborative Innovation Center of Chinese Medicinal Resources Industrialization, Shaanxi University of Chinese Medicine, 712046 Xi’an, Shaanxi Province China; 2 Jiangsu Key Laboratory for High Technology Research of TCM Formulae, Jiangsu Collaborative Innovation Center of Chinese Medicinal Resources Industrialization, and National and Local Collaborative Engineering Center of Chinese Medicinal Resources Industrialization and Formulae Innovative Medicine, Nanjing University of Chinese Medicine, 210023 Nanjing, Jiangsu Province China; 3 Shaanxi Academy of Traditional Chinese Medicine, 710003 Xi’an, Shaanxi Province China

**Keywords:** Rhubarb, Efficacy, Bioactive component, Fuzzy identification, Integrated effect, Back propagation neural network

## Abstract

**Background:**

Rhei Radix et Rhizoma (rhubarb), as one of the typical representatives of multi-effect traditional Chinese medicines (TCMs), has been utilized in the treatment of various diseases due to its multicomponent nature. However, there are few systematic investigations for the corresponding effect of individual components in rhubarb. Hence, we aimed to develop a novel strategy to fuzzily identify bioactive components for different efficacies of rhubarb by the back propagation (BP) neural network association analysis of ultra-performance liquid chromatography/quadrupole time-of-flight mass spectrometry for every data (UPLC-Q-TOF/MS^E^) and integrated effects.

**Methods:**

Through applying the fuzzy chemical identification, most components of rhubarb were classified into different chemical groups. Meanwhile the integration effect values of different efficacies can be determined by animal experiment evaluation and multi-attribute comprehensive indexes. Then the BP neural network was employed for association analysis of components and different efficacies by correlating the component contents determined from UPLC-Q-TOF/MS^E^ profiling and the integration effect values. Finally, the effect contribution of one type of components may be totaled to demonstrate the universal and individual characters for different efficacies of rhubarb.

**Results:**

It suggested that combined anthraquinones, flavanols and their polymers may be the universal character to the multi-functional properties of rhubarb. Other components contributed to the individuality of rhubarb efficacies, including stilbene glycosides, anthranones and their dimers, free anthraquinones, chromones, gallic acid and gallotannins, butyrylbenzenes and their glycosides.

**Conclusions:**

Our findings demonstrated that the bioactive components for different efficacies of rhubarb were not exactly the same and can be systematically differentiated by the network-oriented strategy. These efforts will advance our knowledge and understanding of the bioactive components in rhubarb and provide scientific evidence to support the expansion of its use in clinical applications and the further development of some products based on this medicinal herb.

**Supplementary information:**

The online version contains supplementary material available at 10.1186/s13020-022-00612-9.

## Introduction

Traditional Chinese medicine (TCM) is an ancient system of traditional medicine developed in China over thousands of years. The basic research of pharmacodynamic substances is the foundation and key in the modernization process of TCM. One of the most distinctive features of herbs is that one medicine can be utilized for multiple diseases. Each herb contains dozens or hundreds of compounds that interact through synergy or antagonism to reflect the overall therapeutic efficacy. Especially for multi-functional TCMs, the types of chemicals are even more diverse. The complexity of their multicomponent nature leads to limitations in identifying bioactive components for different efficacies, which hinders the recognition of TCM in the international market.

Rhei Radix et Rhizoma (rhubarb), as one of the typical representatives of multi-effect TCMs, belongs to the *Rheum* L. genus from the Polygonaceae family, and its application can be traced back to 270 BC in Chinese “*Shen Nong Ben Cao Jing*”. It is also one of the most ancient and important medicinal herbs in many countries. To date, it is not only officially listed in the Chinese Pharmacopoeia but also appears in the British Pharmacopoeia and European Pharmacopoeia [1]. Rhubarb is commonly used for “removing accumulation with purgation” (E1), “clearing heat and purging fire” (E2), “cooling blood and detoxifying” (E3), “eliminating blood stasis to remove obstruction in channels” (E4) and “disinhibiting dampness and removing jaundice” (E5) recorded as main efficacies in many Chinese medical classics and Chinese Pharmacopoeia. In modern research, rhubarb is one of the most effective laxatives and has been used worldwide for the treatment of intestinal constipation [2]. In addition, it also exhibits choleretic [3], renal and hepatic protective [4, 5], antineoplastic [6], hypolipidemic [7], promoting blood circulation [8] as well as other pharmacological effects. Although many reports [9, 10] attributed the biological activities of rhubarb to several classes of compounds, including anthraquinones, flavonoids and their glycosides, stilbene glycosides, chromones, gallotannins, butyrylbenzene glycosides and naphthalene glycosides, it is especially regarded that free and combined anthraquinones are the primary bioactive components of rhubarb [11, 12]. However, the contributions of these components to different efficacies of rhubarb are still unknown and lack systematic studies currently. Therefore, it is an urgent scientific problem to clarify main effective substances for different efficacies of rhubarb.

Although many methods have been recently developed to investigate the bioactive components for the pharmacological functions of herbal medicines [13–20], no attempt has been made to touch upon the systemic research of multiple effects, since each herbal efficacy corresponds to various bioactive components. Following our previous attempt [21–25], we were predominantly inspired by the idea that most components in herbal medicines can be grouped into a class of chemical structure types, and they may be assigned to different chemical groups by a fuzzy chemical identification strategy without the need to identify their exact chemical structures. The correlation analysis of chemical components and effects can be further used to identify active component groups, which may give explanations for the chemical nature of complex multifunctional TCMs. Back propagation (BP) neural network has emerged as an advanced correlation model with high prediction accuracy and optimization capability [26, 27]. It can be tailored to reveal the substance basis [28], spectrum-effect relationship [29] or compatibility application [30] of TCM by linking certain chemical constituents to pharmacodynamic index data. However, unlike previous studies, we tried to investigate as many components as possible to distinguish different effects, rather than subjectively limited some known components as relevant targets. As a result, we can find the regularity of TCM component action system, which coincided with the holistic view in the study of multi-effect herbs.

**Fig. 1:**
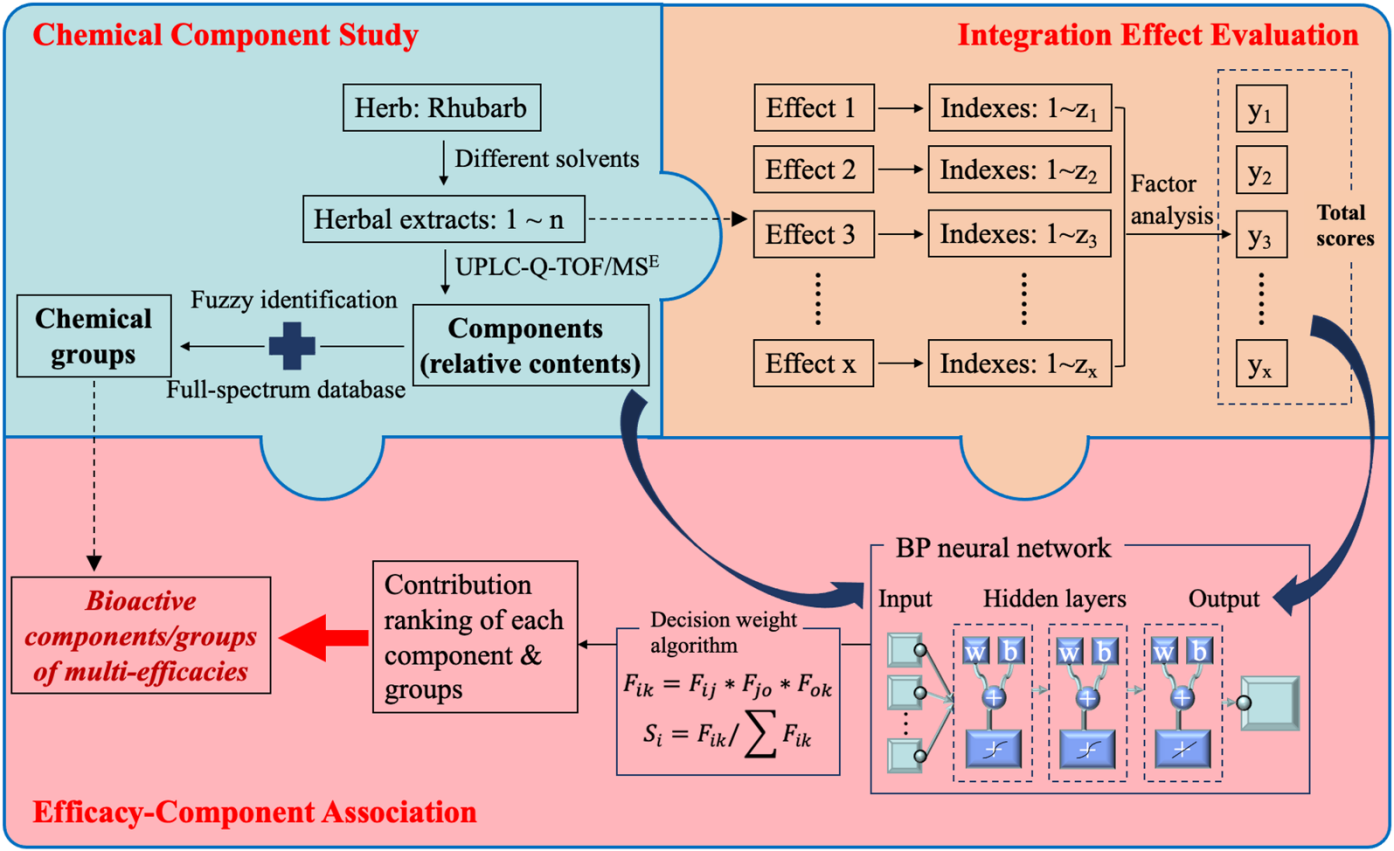
Summary diagram of the developed strategy and approach

Here, we aimed to develop a novel strategy to fuzzily identify bioactive components for different efficacies of rhubarb through the BP neural network association analysis of ultra-performance liquid chromatography/quadrupole time-of-flight mass spectrometry for every data (UPLC-Q-TOF/MS^E^) and integrated effects (Fig. 1). By applying the fuzzy chemical identification method, most components of rhubarb were easily assigned to different chemical groups, and further their relative contents in different extracts were determined by UPLC-Q-TOF/MS^E^ profiling. Meanwhile, the integrated effect values of different efficacies can be obtained by animal experiment evaluation and multi-attribute comprehensive indexes. Subsequently, the BP neural network was employed to correlate component contents with integration effects. Finally, the contributions of each component on different efficacies were quantitatively characterized, and further the effect contributions of certain component types were able to be cumulated to demonstrate the universal and individual characters of rhubarb efficacies. In this study, the methodology was successfully applied to comprehensively differentiate the bioactive components in rhubarb via quantifying their contributions to the five major efficacies. These efforts will advance our knowledge and understanding of the chemical nature for different efficacies of rhubarb, and provide scientific evidence to support the expansion of its use in clinical applications and the further development of some products based on this medicinal herb.

## Materials and methods

### Reagents and equipment

Anhydrous ethanol (analytical grade ≥ 99.7%), methanol (HPLC grade) and dimethyl sulfoxide (DMSO, ≥ 99.9%) were purchased from Merck. Lipopolysaccharide (LPS, L2880) was from Sigma. α-Naphthyl isothiocyanate (ANIT, E1909091) was from Aladdin. White sugar (20,180,404), Yili whole milk powder (20,180,409), Jinluo edible lard (20,180,317) and Yujiangyuan virgin olive oil (20,191,008) were bought from the local supermarket. Adrenalin hydrochloride (Adr, 1 mg/mL for livestock, 20,190,307) was bought from Shanghai Quanyu Biotechnology Animal Pharmaceutical Co., Ltd. Drugs included Cisapride Tablet (20,180,518, Anglikang), Compound Danshen Dripping Pill (170,912, Tasly), Ursodeoxycholic Acid Capsule (L19001A, Kangzhe) and 28 chemical references of rhubarb (purity ≥ 98%, Nanjing Jin Yibai Biological Technology Co., Ltd., details listed in Additional file 1: Table S1). Antibodies used for western blotting were the same as our preceding research [31] and provided in the Supplementary Materials. Mouse enzyme-linked immunosorbent assay (ELISA) kits covering MTL, SS, VIP, AchE, TG, Na^+^-K^+^-ATPase, TNF-α, IL-1β, IL-6, HSP-70, (T-)SOD, NO, TXB_2_, 6-keto-PGF_1α_, PGE_2_, ET-1, Mg^2+^, Ca^2+^, MDA, GSH, ALT, AST, ALP, GST, GGT, TBIL, DBIL and TBA were all obtained from Shanghai Enzyme-linked Biotechnology Co., Ltd. Kits of coagulation four indices of TT, PT, APTT and FIB were from Steellex. Serum and tissue Fe^3+^ test kits (colorimetry, A039-1-1, A039-2-1) were from Nanjing Jiancheng Bioengineering Institute. The experiments were equipped with micro-plate reader (EnSpire, Molecular Devices), protein electrophoresis apparatus (PowerPac, BioRad), gel imager (#1,708,195, BioRad), platelet aggregation/coagulation analyzer (LG-PABER-1, Steellex), and UHPLC Acquity™ system coupled to Synapt™ Q-TOF mass spectrometer (Waters Corp.).

### Preparation of rhubarb liquids

The rhubarb was collected from Gannan Prefecture, Gansu Province, China, and was processed into decoction pieces by Gannan Baicao Biotechnology Development Co., Ltd. They were identified by Professor Hui Yan (Department of Pharmacognosy, Nanjing University of Chinese Medicine) as *Rheum tanguticum* Maxim. ex Balf. (No. NJUTCM-20,171,015) [32]. According to the data mining of multifunctional preparation and usage of rhubarb before [33], it was found that the most commonly used extraction methods were water and wine decoction repeated twice for 15–200 min. Based on the preliminary detection of component changes among different extraction methods (Additional file 1: Figure S1), we designed the rhubarb groups from two aspects of 9 different concentrations of ethanol-water (EW) solvents and 2 extraction time. Short time (S) was 10 min for the first decoction and 5 min for the second while long time (L) was 120 min for the first and 80 min for the second. A total of 18 rhubarb groups were set up including water-S, water-L, 10% EW-S, 10% EW-L, 20% EW-S, 20% EW-L, 35% EW-S, 35% EW-L, 50% EW-S, 50% EW-L, 65% EW-S, 65% EW-L, 80% EW-S, 80% EW-L, 90% EW-S, 90% EW-L, ethanol-S, ethanol-L. The highest dose of Chinese Pharmacopoeia was selected to be converted to 1.95 g/kg for mouse dosage. The process was to precisely weigh rhubarb powder passing through No.4 Pharmacopoeia sieve, fully soak with 2 times the amount of extraction solvent and then add 8 times the amount of corresponding solvent to boil out by reflux twice. After merging two filtrates when they were hot, the extracting solution was concentrated till no alcohol taste using rotatory evaporation (50 °C), and finally diluted with water as the suspension of 0.195 g/mL for use.

### Pretreatment of rhubarb samples and chemical references

Rhubarb samples: Before vacuum concentration, 1 mL of each liquid was pipetted and accurately added into the homologous solvent to a concentration of 0.03 g/mL. Then 100 µL of each sample was mixed as the quality control (QC) sample. Reference solution: The 28 chemical references were critically weighed 1 mg each adding with a trace amount of DMSO for hydrotropy and methanol till 1 mL, followed by ultrasound to become the transparent solution. 100 µL of each mother liquor was added to 1 mL of methanol for dilution, and then 100 µL of each dilution was taken as the mixing standard sample. All above samples were placed in HPLC glass vials through microfiltration membrane (0.22 μm) and stored at 4 °C for testing.

### UPLC-Q-TOF/MS^E^ profiling

To make the relative content determination of liquid ingredients more accurate, the acquisition time of mass spectrometry scanning was changed from 0.3 s to 0.1 s, and the wavelength range of ultraviolet (UV) detection was set at 190–420 nm. The phase A was water containing 0.1% formic acid and B was acetonitrile. We optimized the gradient elution program: 0 ~ 2 min, 3 ~ 10% B; 2 ~ 5 min, 10 ~ 15% B; 5 ~ 8 min, 15% B; 8 ~ 11 min, 15 ~ 23% B; 11 ~ 16 min, 23 ~ 30% B; 16 ~ 20 min, 30 ~ 60% B; 20 ~ 24 min, 60 ~ 80% B; 24 ~ 25 min, 80 ~ 3% B. Remaining device parameters were the same as we described previously [34]. At the beginning of the analytical batch, QC sample was continuously injected 5 times to balance the system. Then 18 rhubarb samples were injected successively, and each sample was parallel for 3 times. Regular injection of corresponding blank solvent and QC samples can be used to evaluate the reliability and repeatability of instrument status throughout the workflow sequence. Under the same conditions, 28 rhubarb standard samples and the mixing standard sample were detected in turn.

### Fuzzy chemical identification

By the fuzzy chemical identification method [23], TCM components can be identified quickly, in which the core is to classify uncertain ones because compounds with the same mother nuclei possess similar action properties and interaction rules. It not only avoids some defects such as complex identification procedures of a great many components or scarcity of chemical references, but also reflects the vague and holistic view of TCM. It needs to be implemented in conjunction with the full-spectrum information database of nearly 300 chemical compounds of rhubarb (Additional file 1: Table S2) we established through querying numerous relevant literature reports and network platforms such as TCMSP (tcmspw.com), SymMap (www.symmap.org) and PubChem (pubchem.ncbi.nlm.nih.gov). Under the premise of selecting different types of known components with high contents in rhubarb as the reference, we summarized the peak order, fragmentation information and pathways of different categories, and then established corresponding compound group networks to recognize unknown components rather than to confirm all components necessarily.

### Mouse models and determination of pharmacodynamic indexes

BALB/c mice, weighing 22 ± 2 g, were purchased from Shanghai Sippr-BK Laboratory Animal Co., Ltd. (license number: SCXK (Hu) 2018-0006). Adaptive feeding environment and all animal related procedures were strictly in accordance with the criteria of the Animal Ethics Committee of Nanjing University of Chinese Medicine. These mice were free to eat conventional food and drink water. Before modeling, they were stratified by weight and randomly divided into 21 groups including the control, model, positive and 18 rhubarb-treated groups.

#### Constipation model for evaluation of E1 and E2

The constipation model with gastrointestinal accumulated heat induced by dyspepsia was based on our established method [31]. The positive drug was Cisapride Tablet for the treatment of gastrointestinal dynamic diseases, and its maximum dosage was 3.9 mg/kg converted for mice. We divided 168 male mice (No. 20,180,006,004,662) into groups of 8 on average. Besides the control group, 0.8 mL of self-made high-calorie feed mixed by sugar, milk powder and lard [31] was additionally given to each mouse by intragastric administration 3 times a day at an interval of 6 h. The modeling lasted seven days. Positive and rhubarb-treated groups were administered with corresponding drug liquids (0.01 mL/g) in the morning of the 6th to 8th day while the control and model groups were intragastrically given isodose water at the same time. After 30 min of administration on day 8, all mice were intragastrically given Indian ink (0.01 mL/g) and immediately placed in the metabolic cages alone to observe defecation characteristics of first black stool time, the number of black stools and fecal weights within 12 h. On the 9th day, we weighed these mice, collected their serum, and then dissected 0.5 cm of duodena behind the pylorus, stomachs, colons and colonic contents. Biochemical index levels of MTL, SS, VIP, TG in mouse serum and AchE, Na^+^-K^+^-ATPase, TNF-α, IL-1β in duodenal tissues were detected by ELISA, and expressions of common inflammatory proteins in the colon covering p-NF-κB p65, NF-κB p65, p-p38, p38, p-ERK, ERK, p-JNK, JNK and TLR4 were determined by western blotting as depicted previously [31].

#### Blood stasis syndrome for evaluation of E3 and E4

The blood stasis syndrome induced by noxious heat can be established by LPS-induced inflammation combined with Adr-induced stagnation of the circulation of vital energy [35]. Existing literature reports basically used LPS to simulate the process of exogenous heat toxin, and its modeling dose for mice was 3 mg/kg prepared by 0.9% normal saline (NS). Adr injection for livestock will assist to achieve the effect of blood stasis in a short time, and its combined dosage was determined as 5 mg/kg through our continuous exploration in order to greatly reduce the mortality of mice. In this model, Compound Danshen Dripping Pill was chosen as the positive drug (105.3 mg/kg for the maximum dose of mice), which was often used clinically to regulate Qi and activate blood circulation. We separated 252 female mice (No. 20,180,006,007,816) into 12 mice in each group. On the first day of modeling, except injecting isodose 0.9% NS for the control group, all mice were intraperitoneally injected with LPS at 0.01 mL/g for the first time and their anal temperatures should be measured punctually 4 h later. If the temperatures were abnormal, the model was successful, and then corresponding drug liquids were administered once every night (0.01 mL/g, isodose water given to the control and model groups) for 6 consecutive days. On the 5th day, mice were subjected to LPS at 0.01 mL/g by intraperitoneal injection and tested for anal temperatures 4 h later again. On the 6th day, they were given two subcutaneous injections of Adr at 0.005 mL/g with a four-hour interval while the control group was accordingly injected with 0.9% NS. After fasting for at least 12 h, they were weighed and sacrificed on day 7. We randomly collected orbital blood of six mice in each group first, added 3.2% sodium citrate anticoagulant into their blood (1:9, v/v) immediately, and pipetted plasma by centrifugation to detect coagulation four indices of TT, PT, APTT and FIB. Secondly, serum from the remaining mice in each group was collected for ELISA levels of HSP-70, SOD, NO, TNF-α, IL-1β, IL-6, TXB_2_, 6-keto-PGF_1α_, PGE_2_, ET-1, Mg^2+^ and Ca^2+^. Thirdly, all mice needed precise weighing of their spleens and thymuses. In addition, colon tissues were used to detect expression levels of the above 9 common inflammatory proteins by western blotting likewise.

#### Cholestasis for evaluation of E5

ANIT can cause acute liver injury and has been used to establish the model of cholestatic jaundice in many studies. Through pretesting, we determined its modeling dose of 75 mg/kg for mice. The positive drug was Ursodeoxycholic Acid Capsule in this experiment, which can be cholagogic effectively at the maximum mouse equivalent dosage of 91 mg/kg. Male mice (No. 20,180,006,009,949) were separated into an average of 8 per group. Positive and rhubarb groups were treated by prophylactic administration (0.01 mL/g) once a day for 3 days [3] while the control and model groups were given the same dose of water. On the 4th day, all mice suffered intragastrically 0.01 mL/g of ANIT that dissolved in olive oil except the control group (equal dose of olive oil), and then were administered with corresponding drug liquids once after 6 h. They were given drug liquids again on the fifth night and fasted overnight. On day 6 (i.e., 48 h after ANIT modeling), we weighed them, drawn blood to store serum, and then took the whole livers and gallbladders for exact weighing respectively. Test kits were applied to detect levels of T-SOD, MDA, GSH, Fe^3+^ in mouse liver and ALT, AST, ALP, GST, GGT, TBIL, DBIL, TBA, Fe^3+^ in serum. The liver homogenate was prepared as follows: 50 mg of liver tissues of each mouse were added with 0.9% NS (20 µL/mg) and two steel balls, and put into an automatic grinding machine for 3 min. The homogenate was placed on the ice for 30 min and centrifuged at 4 °C to get the supernatant for determination.

### BP neural network correlation method

The relative content of each component in 18 rhubarb samples was correlated with 5 integration effects severally using the BP neural network. It mainly consisted of three steps. (1) Data normalization: y = (x - MinValue)/ (MaxValue - MinValue), where x and y were the values before and after conversion, and MaxValue and MinValue were the maximum and minimum values in samples. (2) Data set division: taking all component contents as input and corresponding integration effect values as output, and all of 1 ~ 18 group data as training samples, among which 90% EW-L, ethanol-S and ethanol-L groups were taken as test samples. (3) Quantity-effect correlation: based on BP neural network algorithm, adjusting various parameters to optimize the model for relevant investigation between components and efficacies, so as to reveal the influence of each component on the overall effect.

### Statistical analysis

Experimental data were processed by GraphPad Prism 7.0 and SPSS 22.0, and expressed as “mean ± standard deviation (SD)”. One-way analysis of variance or independent sample *t*-test was carried out to compare the data between groups. Bilateral *P* value less than 0.05 was considered to be statistically significant.

## Results

### Semi-quantitative and qualitative analysis of rhubarb components based on UPLC-Q-TOF/MS^E^ and fuzzy chemical identification

**Fig. 2:**
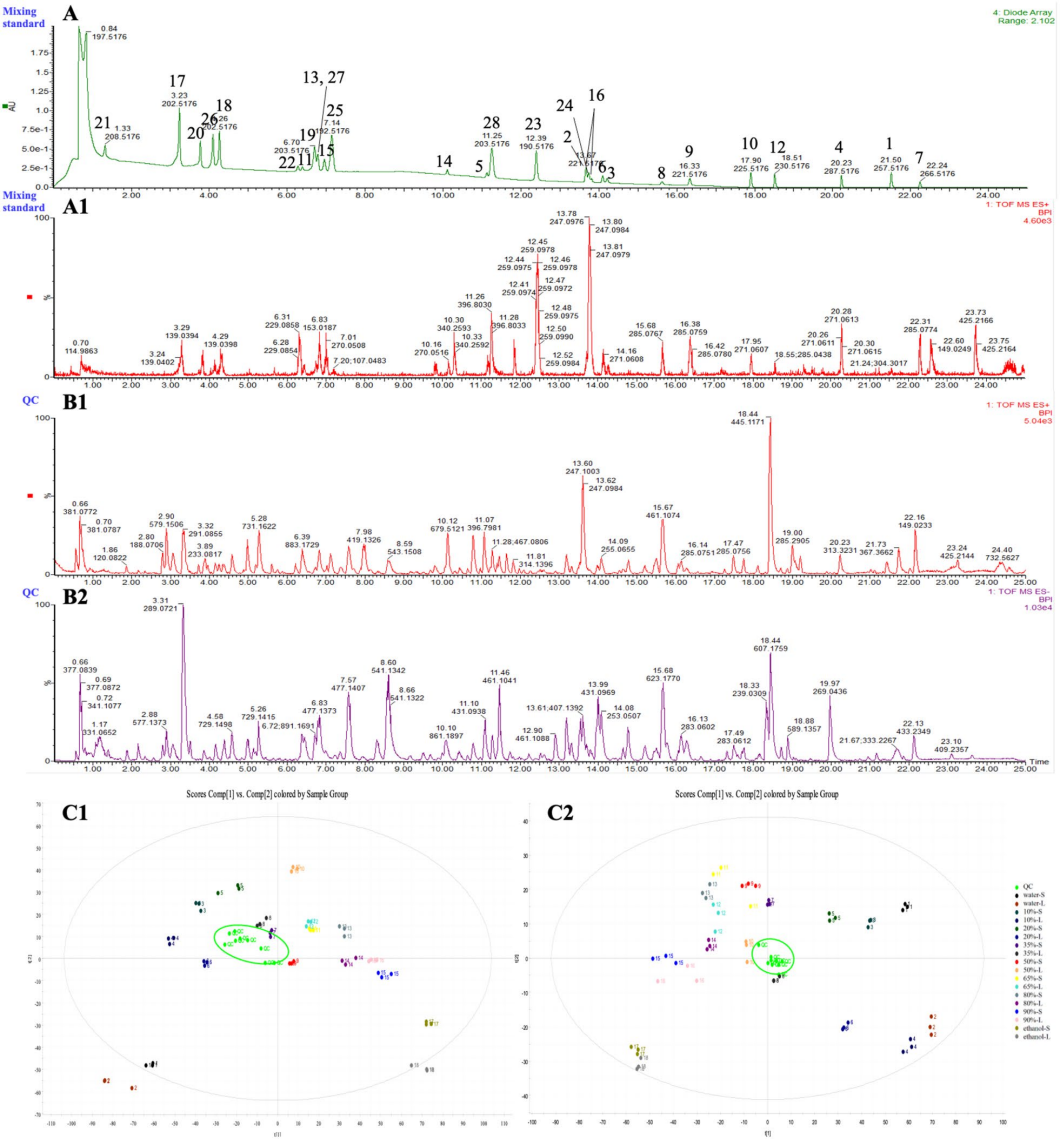
UV spectrum (**A**) and BPI chromatogram (A1) of the mixing standard sample covering 28 chemical references; Representative BPI chromatograms of the rhubarb QC sample (**B**) and PCA plots of all rhubarb groups (**C**) detected in positive (1) or negative (2) ion modes

The mixing standard sample basically achieved good separation within 25 min, which can provide a reference for qualitative analysis of various compounds. In PCA plots, QC samples were all close to the center of the Hotelling T2 ellipse, while 18 rhubarb groups were obviously separated but the same group of repeated sampling was clustered together. These indicated the stability of the UPLC-Q-TOF/MS^E^ method (Fig. 2). Combined with retention time (RT) and base peak intensity (BPI) from total ion chromatograms, we screened out 108 unknown components with high responsiveness and different variation trends from 18 rhubarb samples. Peak areas, that is, relative contents of all components, were listed in Additional file 1: Table S3 calculated by EZinfo 2.0 software.

**Fig. 3:**
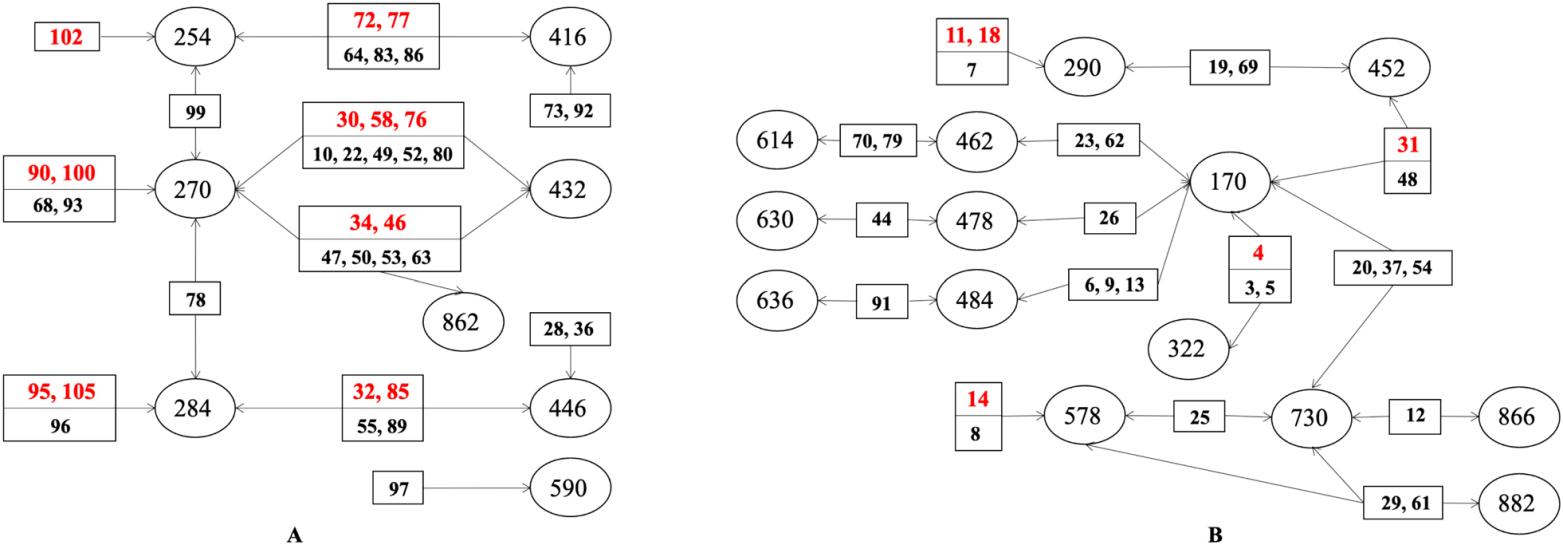
Networks of compound groups taking high-content anthraquinones (**A**), and flavanols and their polymers, gallic acid and gallotannins (**B**) for examples (Inside the circle is the molecular weight determined by molecular ion peaks in positive and negative ion modes; Inside the box is the serial number of each component of rhubarb samples, and red means components confirmed by chemical references)

Furthermore, according to MS^E^ characteristic fragmentation patterns of references, 25 components were confirmed via matching the 108 components. With reference to the full-spectrum information database of chemical compounds of rhubarb in Additional file 1: Table S2, we constructed compound group networks of different categories (Fig. 3) to classify the remaining components (Table 1).

**Table 1:** Classification of 108 rhubarb components on the basis of fuzzy chemical identification

Category^▲^	Confirmed component (No., RT)	Remaining component attribution (RT)
Rh-01	Aloe-emodin (**90**, 17.70)	**68** (12.58), **78** (14.51), **93** (18.13), **96** (18.46), **99** (19.27)
	Rhein (**95**, 18.33)	
	Emodin (**100**, 19.99)	
	Chrysophanol (**102**, 21.24)	
	Physcion (**105**, 22.00)	
Rh-02	Aloe-emodin 8-*O*-*β*-D-glucoside (**30**, 6.31)	**10** (3.11), **22** (4.76), **28** (5.65), **36** (6.97), **49** (9.84), **52** (10.28), **55** (10.64), **64** (11.67), **80** (15.11), **83** (15.72), **86** (16.24), **89** (17.48), **92** (17.76), **97** (18.94)
	Rhein 8-*O*-*β*-D-glucoside (**32**, 6.60)	
	Emodin 1-*O*-*β*-D-glucoside (**58**, 10.97)	
	Chrysophanol 1-*O*-*β*-D-glucoside (**72**, 13.50)	
	Emodin 8-*O*-*β*-D-glucoside (**76**, 13.90)	
	Chrysophanol 8-*O*-*β*-D-glucoside (**77**, 14.05)	
	Physcion 8-*O*-*β*-D-glucoside (**85**, 16.12)	
Rh-03	Sennoside B (**34**, 6.82)	**47** (9.57), **50** (9.96), **53** (10.38), **63** (11.41), **73** (13.52)
	Sennoside A (**46**, 9.56)	
Rh-04	Cianidanol (**11**, 3.24)	**7** (2.75), **8** (2.83), **12** (3.29), **19** (4.25), **20** (4.45), **25** (5.13), **29** (6.19), **37** (7.11), **54** (10.48), **61** (11.29), **69** (12.79), **84** (16.06)
	(-)-Epicatechin (**18**, 4.24)	
	(-)-Epicatechin gallate (**31**, 6.60)	
	Procyanidin B2 (**14**, 3.76)	
Rh-05	Gallic acid (**4**, 1.38)	**3** (1.19), **5** (1.84), **6** (2.12), **9** (3.00), **13** (3.40), **23** (4.76), **26** (5.18), **44** (8.83), **62** (11.34), **70** (13.09), **79** (14.66), **91** (17.74)
Rh-06	Resveratroloside (**24**, 4.86)	**40** (7.79), **42** (8.01), **43** (8.28), **57** (10.82), **104** (21.72)
Rh-07	Torachrysone 8-*O*-glucoside (**74**, 13.55)	**21** (4.52), **81** (15.44)
Rh-08	Raspberryketone glucoside (**17**, 4.10)	**39** (7.68), **60** (11.16), **75** (13.73), **82** (15.60), **87** (16.61)
	Lindleyin (**33**, 6.70)	
	4-(4-Hydroxyphenyl)-2-butanone (**38**, 7.41)	
Rh-09	5-Acetyl-7-hydroxy-2-methyl-chromone (**59**, 11.06)	**15** (3.82), **16** (3.90), **41** (7.99), **48** (9.68), **56** (10.66), **98** (19.09)
Rh-10	-	**65** (11.72), **67** (12.10), **101** (21.08), **103** (21.31)
Others	-	**1** (0.81), **2** (0.94), **27** (5.35), **35** (6.96), **45** (9.31), **51** (10.12), **66** (11.90), **71** (13.25), **88** (17.26), **94** (18.15), **106** (22.28), **107** (23.23), **108** (23.77)

^▲^ Rh-01 ~ 10 represent free anthraquinones, combined anthraquinones, anthranones and their dimers, flavanols and their polymers, gallic acid and gallotannins, stilbene glycosides, naphthalene glycosides, butyrylbenzenes and their glycosides, chromones, and flavonoid (flavonol) glycosides in turn

### Evaluation of five integration effects based on factor analysis

TCM syndromes cannot tend to be completely cured by a single effect, so we explore two or even more effects in an animal model to improve the research efficiency and fitness with actual symptoms. Mice’s performance of the first constipation model, selection of E1 and E2 indexes and their pharmacodynamic changes were basically consistent with our previous discussion [31]. First black stool time, the number of black stools, fecal weights within 12 h, colonic content weights, organ coefficients of colon and stomach, MTL, SS, VIP and AchE can reflect E1 (Additional file 1: Figure S2). Expression levels of TG, Na^+^-K^+^-ATPase, TNF-α, IL-1β and grayscale ratios of the aforementioned 9 colonic inflammatory proteins can represent E2 (Additional file 1: Figure S3).

The treatment of the second blood stasis syndrome induced by noxious heat also included two aspects of both cooling and activating blood. Model mice appeared obvious symptoms of blood stasis with enlarged spleens and decrescent thymuses (*P* < 0.001), while drug groups’ spleens recovered to some extent. On the one hand, we took anal temperatures, HSP-70, SOD, NO, TNF-α, IL-1β, IL-6 and the same 9 levels of colonic inflammatory proteins as E3 indicators (Additional file 1: Figure S4), which resembled E2 that played an anti-inflammatory role from the perspective of Western medicine. Mice maintained opposite hypothermia (*P* < 0.001) in febrile response to LPS, and some drug groups were able to significantly antagonize this toxic reaction. Compared with the model, most groups down-regulated the levels of inflammatory factors (*P* < 0.05), especially phosphorylated proteins and TLR4 (*P* < 0.001) in high-proportioned ethanol-extracted rhubarb groups. It may be because LPS can act through the activation of NF-κB signaling pathway mediated by TLR family in connection with pro-inflammatory cytokines [36]. On the other hand, indicators of E4 (Additional file 1: Figure S5) covered TT, PT, APTT, FIB, TXB_2_, 6-keto-PGF_1α_, ratios of TXB_2_ to 6-keto-PGF_1α_, PGE_2_, ET-1, Mg^2+^ and Ca^2+^. The positive group had a better improvement on coagulation four indices, but most of rhubarb groups only showed significant callback to PT and FIB (*P* < 0.05). Ethanol-extracted rhubarb corrected the imbalance of elevatory values of TXB_2_/6-keto-PGF_1α_ (*P* < 0.001), which facilitated blood circulation [35]. Prostaglandins can dilate blood vessels whereas endothelins constricted them, which all displayed callback tendency of varying degrees in different drug groups.

With regard to the third cholestasis model, as shown in Additional file 1: Figure S6, model mice performed visibly declining weights (*P* < 0.01), darkening serum color, intumescent gallbladders and inky bile accompanied by obvious liver damage of which there were dense bleeding points on the surface. The overall state of the positive group presented relatively well recovery, and these lesions also improved to a certain degree in part of rhubarb-treated groups. Whereupon we investigated measurement indexes associated with jaundice including organ coefficients of liver and gallbladder, T-SOD, MDA, GSH, tissue Fe^3+^, ALT, AST, ALP, serum Fe^3+^, GST, GGT, TBIL, DBIL and TBA to evaluate E5 (Additional file 1: Figure S7). The level regulation of oxidative stress in liver tissues (*P* < 0.05) by rhubarb decocted for a long time may be one of the main mechanisms of removing jaundice. Except GGT and DBIL, other serum liver function levels were generally elevated in model mice and decreased prominently (*P* < 0.05) in most rhubarb-treated groups.

Factor analysis was carried out for the above pharmacodynamic indexes of five effects to obtain their integration effect values respectively. Referring to the approach we had described [31], comprehensive weight scores of each group were calculated from the scoring coefficient matrix of indicator variables (Additional file 1: Tables S4–S8), and then Kolmogorov-Smirnov normal distribution test was utilized to examine the results (Additional file 1: Tables S9–S13). At last, as illustrated in Table 2, there existed significant differences between the model and control groups. The therapeutic efficacy of positive drugs emerged very obviously in all five effects while the effectiveness of rhubarb with different preparation was not exactly the same. E1 was conspicuous in groups of 10% EW-S ~ 35% EW-L and 80% EW-L ~ ethanol-L. High-concentration ethanol groups possessed better E2 on the whole. E3 of rhubarb extracted by over 20% EW was all evident, particularly for the long-time extraction, which conformed to the TCM theory that steaming with wine can be good at clearing blood-aspect heat toxin. In contrast, E4 of rhubarb was relatively little and only worked when it was prepared with water or more than 80% ethanol. 10% EW-L ~ ethanol-L groups showed prominent E5 among which three groups of 35% EW-L, 50% EW-L and 65% EW-L were the best, suggesting that “wine and water co-decoction” may be more conducive to exert the curative effect. Thus, it suggested that different extraction methods were adept at different effects.

**Table 2:** Integration effects of rhubarb on the basis of factor analysis (*n* = 6 ~ 8, mean ± SD)

Groups	E1	E2	E3	E4	E5
Control	(2.08 ± 0.28)	(− 2.53 ± 1.96)	(− 1.24 ± 3.53)	(1.89 ± 0.60)	(− 1.07 ± 2.78)
Model	(− 5.48 ± 1.96)^###^	(5.39 ± 3.77)^###^	(4.40 ± 3.62)^#^	(− 0.89 ± 1.86)^##^	(5.40 ± 2.36)^###^
Positive	(1.65 ± 3.91)***	(0.01 ± 2.12)***	(− 0.88 ± 2.32)**	(1.70 ± 1.78)*	(− 0.48 ± 3.09)**
water-S	(− 0.58 ± 2.56)***	(− 0.32 ± 3.04)**	(2.65 ± 3.50)	(0.62 ± 1.77)	(3.60 ± 1.53)
water-L	(− 0.35 ± 1.67)***	(0.12 ± 0.42)**	(1.22 ± 2.90)	(1.92 ± 2.34)*	(2.70 ± 3.69)
10%-S	(0.87 ± 0.80)***	(− 0.83 ± 1.89)***	(2.27 ± 2.72)	(1.60 ± 3.66)	(3.22 ± 1.77)
10%-L	(0.02 ± 2.53)***	(0.30 ± 0.38)**	(0.91 ± 2.04)	(0.77 ± 0.63)	(1.10 ± 4.00)*
20%-S	(0.29 ± 2.42)***	(0.64 ± 3.21)**	(1.65 ± 3.36)	(0.08 ± 1.24)	(1.27 ± 3.26)*
20%-L	(0.52 ± 2.09)***	(1.93 ± 2.98)*	(− 0.82 ± 3.59)*	(0.01 ± 1.80)	(0.45 ± 2.58)**
35%-S	(0.09 ± 3.00)***	(1.22 ± 3.76)*	(− 0.60 ± 4.00)*	(− 0.98 ± 2.97)	(0.35 ± 3.84)**
35%-L	(0.80 ± 2.55)***	(1.48 ± 3.52)*	(− 0.50 ± 1.44)*	(− 0.33 ± 0.74)	(− 0.97 ± 1.73)***
50%-S	(− 0.55 ± 2.25)***	(− 0.11 ± 2.62)**	(− 0.27 ± 3.83)*	(− 0.94 ± 3.69)	(− 0.21 ± 3.89)**
50%-L	(0.13 ± 2.98)***	(1.10 ± 2.48)*	(− 0.82 ± 1.42)*	(− 0.17 ± 2.32)	(− 1.01 ± 2.29)***
65%-S	(− 1.84 ± 2.63)**	(− 1.10 ± 3.09)***	(− 0.61 ± 2.24)*	(− 0.69 ± 3.38)	(− 0.32 ± 2.39)***
65%-L	(− 0.29 ± 3.30)**	(-0.53 ± 2.24)***	(− 1.92 ± 2.01)**	(− 0.66 ± 2.06)	(− 1.55 ± 3.29)***
80%-S	(− 0.67 ± 3.18)**	(− 1.38 ± 2.57)***	(− 0.12 ± 3.36)*	(− 0.52 ± 2.21)	(0.14 ± 3.88)**
80%-L	(0.47 ± 3.49)***	(− 0.76 ± 3.02)**	(− 1.71 ± 2.02)**	(0.89 ± 2.31)	(− 0.37 ± 3.48)**
90%-S	(1.00 ± 3.00)***	(− 1.43 ± 2.71)***	(− 2.25 ± 2.69)**	(1.58 ± 1.49)*	(− 0.76 ± 3.76)**
90%-L	(0.47 ± 2.38)***	(− 2.11 ± 1.25)***	(− 2.40 ± 3.29)**	(0.66 ± 1.79)	(− 0.42 ± 3.00)***
ethanol-S	(1.42 ± 3.08)***	(− 3.39 ± 1.99)***	(− 1.96 ± 2.34)**	(1.22 ± 2.91)	(− 0.49 ± 3.86)**
ethanol-L	(2.19 ± 3.40)***	(− 2.61 ± 0.61)***	(− 1.25 ± 1.54)**	(0.98 ± 1.44)	(0.08 ± 3.96)**

^#^ *P* < 0.05, ^##^ *P* < 0.01, ^###^ *P* < 0.001 compared with the Control;

* *P* < 0.05, ** *P* < 0.01, *** *P* < 0.001 compared with the Model

### Establishment of quantity-effect correlation method based on BP neural network

The advanced BP neural network mimics neurons based on the structure and function of the biological brain with high prediction accuracy and optimization ability [26, 27]. It can be intelligently applied in the fitting correlation analysis to help us determine the contribution degree of each chemical component to the corresponding efficacy. So, we tried to objectively associate as many components detected with efficacies as possible. The reasonable quantity-effect correlation method was established by repeatedly debugging parameters as follows.

**Fig. 4:**
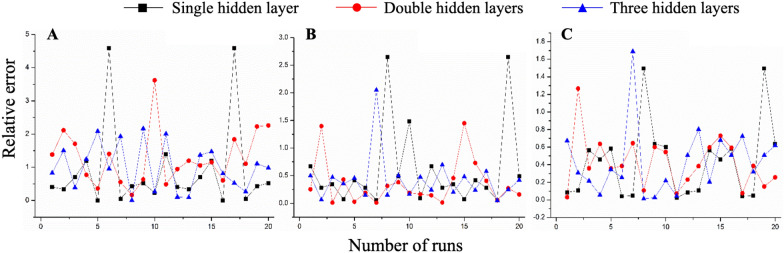
Relative errors between the predicted and true values of 90% EW-L (**A**), ethanol-S (**B**) and ethanol-L (**C**) groups influenced by 1, 2 and 3 hidden layers

**Fig. 5:**
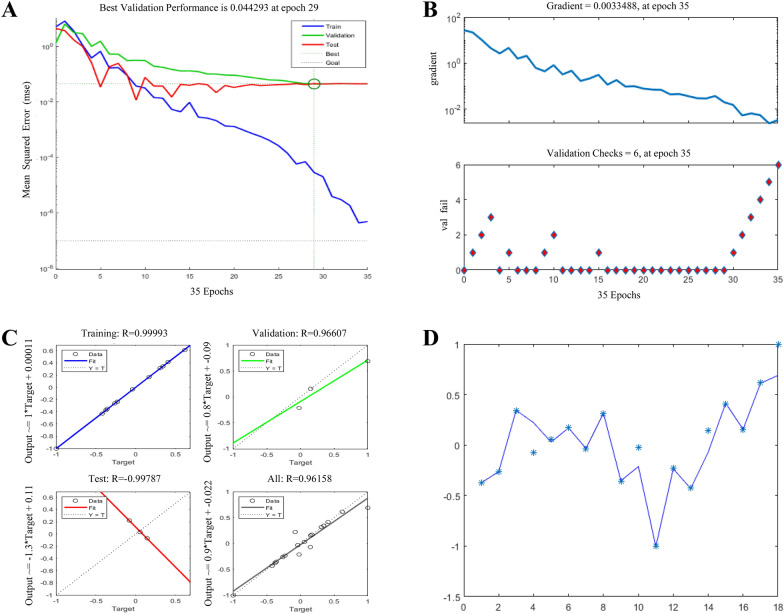
Running effect diagrams of the neural network performance (**A**), training state (**B**), regression analysis (**C**), comparison between the predicted and true values (**D**: the line is the prediction curve and “*” denotes the true value of each group) belonging to E1 as an example (Diagrams of E2 ~ 5 are shown in Additional file 1: Figure S8 b ~ e)

(1) The number of hidden layers: Theoretically, the more hidden layers, the stronger the ability to fit functions is. However, in fact, many layers are apt to cause overfitting as well as increase training difficulty of convergence. On the premise of setting 108 nodes in the input layer and outputting 1 node, we explored the fitting consequence at 1, 2 and 3 layers. The number of nodes in different hidden layers was randomly set to 10, and relative errors between the predicted and true values were consequently compared by repeating 20 runs. It can be seen in Fig. 4 that, although the relative error values of double hidden layers were not optimal, they were more stable and not easy to appear local extremum. Three hidden layers had maximum negative values combined with observing the predicted values of test samples (Additional file 1: Table S14). So, we adopted 2 hidden layers for the next step.

(2) The number of neurons per hidden layer: According to empirical formulas and the setting principle of hidden neurons, in the range of 108 input nodes to 1 output node, the number of neurons was set every 20 values (i.e., 20, 40, 60, 80, 100), and then added 5, 10 and 15 from 0 to 20, 70 and 75 from 60 to 80, 130, 160 and 190 behind 100. The second hidden layer kept pace with the first one. Five sets of data from three test samples were acquired by 5 runs under each number of neurons (Additional file 1: Table S15). Synthetically in view of the predicted, true and their relative error values, we set the number of neurons at 20 due to its steadier relative errors.

(3) Learning rate: In general, it tends to choose small adaptive learning rates to ensure the stability of the system, but too small learning rates will lead to a very long training time. In order to obtain better results, we ran test samples for five times severally when the learning rate was 0.01, 0.02, 0.03, 0.04, 0.05, 0.06, 0.07, 0.08, 0.09, 0.1, 0.2, 0.3, 0.4, 0.5, 0.6, 0.7 and 0.8 (Additional file 1: Table S16). It was found that there were no significant trend changes. Relatively speaking, the learning rate of 0.02 can make BP algorithm with gradient descent preferable.

(4) Running effect: We adjusted the display frequency to 100, training times to 1000 and the minimum error of training target to 0.0000001 for further accurate prediction of this method. After repeated debugging, the final running program was demonstrated in Additional file 1: Figure S8 (a). All training samples and corresponding integration effect values were substituted into respective optimized neural networks. The training will stop when the mean squared error was less than its set point or the gradient reached its set value, and it was forced to end as well when errors did not decrease but rise continuously for 6 times by validation checks of the generalization capability. As a result, the fitting correlation effects were all excellent (Fig. 5).

(5) Decision weight calculation: The above neural network training results only reflected the relationship among neurons. What’s more, we needed to clarify the relative importance of each input factor to output information. Hence, the following neural network learning algorithms were sequentially used to work out decision weights of each input component.

(The input layer: *n* = 108, the first hidden layer: *m* = 20, the second hidden layer: *p* = 20, the output layer: *l* = 1.)

Impact of the *o* unit in the second hidden layer on the *k* output relative to all units:

$${F}_{ok}=\left|{w}_{ok}\right|/\sum\sum\left|{w}_{ok}\right|$$ *o* = 1, 2…*p*, *k* = 1, 2…*l*.

Impact of the *j* unit in the first hidden layer on the *o* unit in the second hidden layer relative to all units:

$${F}_{jo}=\left|{w}_{jo}\right|/{\sum\sum}\left|{w}_{jo}\right|$$ *j* = 1, 2…*m*, *o* = 1, 2…*p*.

Impact of the *i* unit in the input layer on the *j* unit in the first hidden layer relative to all units:

$${F}_{ij}=\left|{w}_{ij}\right|/\sum\sum\left|{w}_{ij}\right|$$ *i* = 1, 2…*n*, *j* = 1, 2…*m*.

Thus, impact of the *i* input on the *k* output:

$${F}_{ik}={F}_{ij}*{F}_{jo}*{F}_{ok}$$

Decision weights of input components can be expressed as:

$${S}_{i}={F}_{ik}/\sum{F}_{ik}$$

### Main bioactive components for five effects of rhubarb

We analyzed the components whose contribution was greater than 0.01 ranking from the ultimate decision weight Additional file 1: Table S17, which were 30 in E1 (Nos. **42** > **21** > **35** > **37** > **34** > **46** > **11** > **98** > **36** > **27** > **59** > **88** > **89** > **18** > **108** > **78** > **105** > **72** > **6** > **55** > **106** > **45** > **61** > **51** > **90** > **32** > **41** > **95** > **38** > **107**), 28 in E2 (Nos. **42** > **7** > **107** > **83** > **29** > **28** > **61** > **98** > **108** > **8** > **80** > **35** > **58** > **81** > **40** > **15** > **92** > **33** > **106** > **36** > **50** > **79** > **11** > **100** > **57** > **19** > **101** > **39**), 35 in E3 (Nos. **24** > **18** > **12** > **86** > **72** > **37** > **51** > **80** > **26** > **55** > **34** > **21** > **16** > **46** > **97** > **42** > **22** > **33** > **88** > **28** > **92** > **27** > **2** > **67** > **70** > **59** > **36**, **44** > **90** > **1** > **82** > **6** > **73** > **4**, **30**), 32 in E4 (Nos. **40** > **81** > **56** > **75** > **69** > **53** > **9** > **64** > **100** > **58** > **49** > **29** > **74** > **83** > **90** > **52** > **63** > **42** > **66** > **106** > **67** > **48** > **31** > **62** > **71** > **89** > **87** > **92** > **20** > **60** > **46** > **36**) and 33 in E5 (Nos. **19** > **55** > **62** > **12** > **33** > **52** > **22** > **35** > **87** > **64** > **69** > **95** > **59** > **42** > **88** > **46** > **43** > **25** > **107** > **72** > **29** > **104** > **71** > **94** > **89** > **99** > **92** > **81** > **36** > **45** > **58** > **108** > **102**). Table 3 summarized the total contribution of each category of rhubarb components. Thereinto, the top 10 of total weights in five effects and of each effect should be focused on respectively (Additional file 1: Tables S18–S23).

**Fig. 6:**
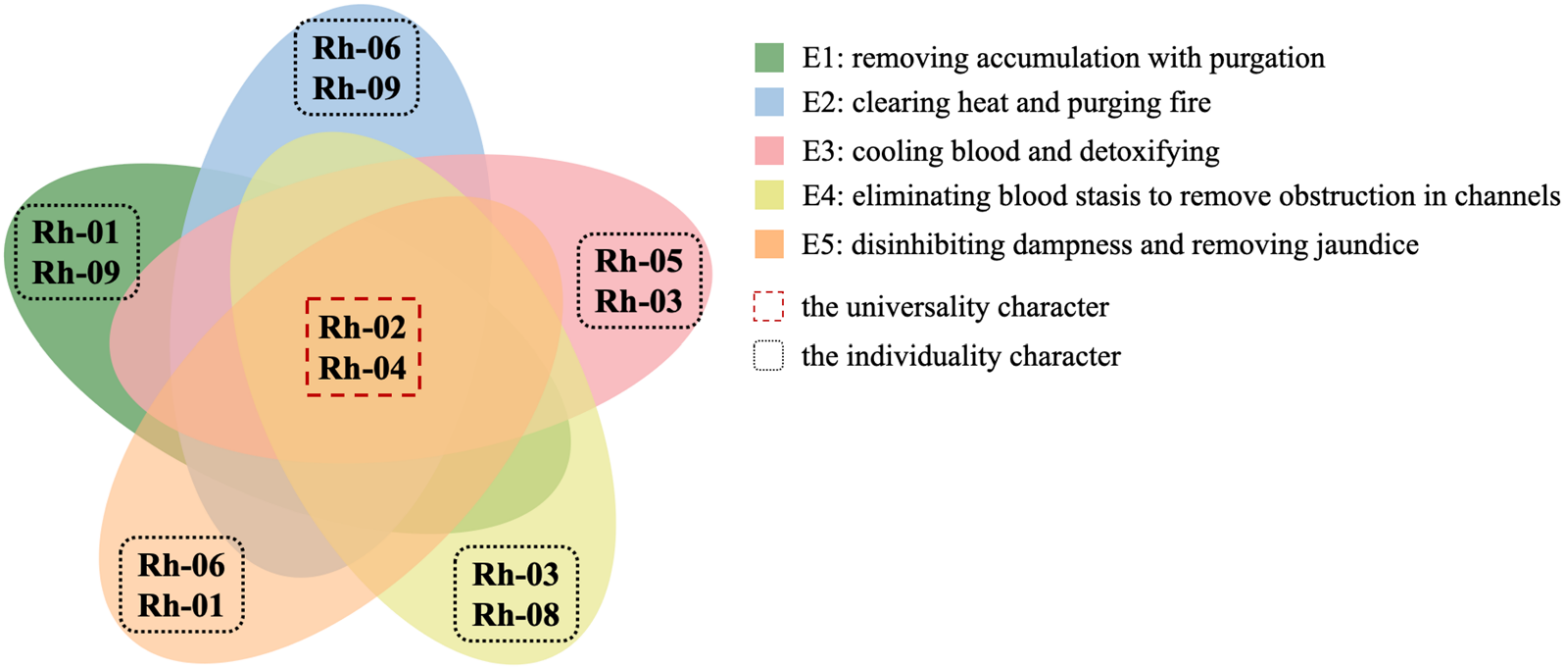
Venn diagram of the universal and individual characters for different efficacies of rhubarb

As intuitively shown in Fig. 6, we constructed the venn diagram to reveal the universal and individual characters of the five effects. It suggested that combined anthraquinones, flavanols and their polymers may be the universal character to the multi-functional properties of rhubarb. Other components contributed to the individuality of rhubarb efficacies, including stilbene glycosides, anthranones and their dimers, free anthraquinones, chromones, gallic acid and gallotannins, butyrylbenzenes and their glycosides.

**Table 3:** Total contribution of each category of rhubarb components with weights over 0.01

Category^▲^	E1	E2	E3	E4	E5	Total
Rh-02	0.05621^(1)^	0.06679^(2)^	0.11795^(1)^	0.09344^(1)^	0.10226^(1)^	0.43665
Rh-04	0.05001^(2)^	0.06684^(1)^	0.04032^(3)^	0.04748^(2)^	0.06193^(2)^	0.26658
Rh-06	0.01966^(6)^	0.03350^(3)^	0.02689^(5)^	0.02719^(5)^	0.03299^(3)^	0.14023
Rh-03	0.02866^(5)^	0.01069^(7)^	0.03469^(4)^	0.03579^(3)^	0.01110^(8)^	0.12093
Rh-01	0.04255^(3)^	0.01052^(9)^	0.01023^(10)^	0.02507^(8)^	0.03212^(4)^	0.12049
Rh-09	0.03611^(4)^	0.02220^(4)^	0.02266^(6)^	0.02550^(7)^	0.01157^(7)^	0.11804
Rh-05	0.01059^(8)^	0.01060^(8)^	0.05335^(2)^	0.02418^(9)^	0.01423^(6)^	0.11295
Rh-08	0.01014^(9)^	0.02084^(5)^	0.02138^(7)^	0.03495^(4)^	0.02452^(5)^	0.11183
Rh-07	0.01925^(7)^	0.01101^(6)^	0.01243^(8)^	0.02694^(6)^	0.01023^(9)^	0.07986
Rh-10	0.00000^(10)^	0.01005^(10)^	0.01060^(9)^	0.01113^(10)^	0.00000^(10)^	0.03178

^▲^ Rh-01 ~ 10 represent free anthraquinones, combined anthraquinones, anthranones and their dimers, flavanols and their polymers, gallic acid and gallotannins, stilbene glycosides, naphthalene glycosides, butyrylbenzenes and their glycosides, chromones, and flavonoid (flavonol) glycosides in turn; The ordinal number after the values represents the order in each efficacy (the smaller the number, the higher the contributions of this category to the efficacy)

## Discussion

The three animal models we selected were tailored to different efficacies of rhubarb. The first model was the constipation with gastrointestinal accumulated heat induced by dyspepsia, which had been established in our preceding report [31]. In brief, it simulated a kind of common TCM syndrome named Yangmingfu-visceraexcess that was mainly characterized by abdominal distension with pain, difficult defecation and accumulated internal heat. The treatment of this syndrome was a comprehensive result of rhubarb exerting effects of E1 and E2. The second model was the blood stasis syndrome induced by noxious heat. At present, rhubarb is also widely used in blood syndrome with considerable clinical effects, among which the blood stasis syndrome is one of the most common clinical syndromes [37, 38]. Under the guidance of TCM theory, blood stasis is closely related to etiology of noxious heat and stagnation of the circulation of vital energy. From the perspective of modern medicine, it is caused by inflammatory reaction, resulting in microcirculation disorders and abnormal blood rheology [39]. LPS combined with Adr modeling can be contributed to study the curative effects of E3 and E4 simultaneously [35, 40, 41]. The third model was cholestatic jaundice reported by Zhang CE et al. [3]. The existing TCM syndromes of Yang-Huang and Yin-Huang have some disadvantages, such as time-consuming modeling, complicated operation steps, many interference factors, poor stability and reproducibility. The drug-induced cholestasis is a well-established alternative model for studying E5 [42, 43]. Hence, we chose these models in mice to carry out studies on rhubarb efficacies.

The hypothesis of “additive effect” was put forward in 2014 [44, 45]. That is, the assemblage or superposition of “effective forms” is the core chemical essence of medicinal herbs. Compounds with identical mother nuclei structure are grouped together and may have the same pharmacological target. Both the “additive effect” of various components on single target and the “synergy effect” on multi-targets, even universal and individual characters of different efficacies, can be well reflected in the Table 3 above, which is also the essence of fuzzy identification our team proposed for new interpretations to pharmacodynamic components and action mechanisms of multi-effect TCMs.

Therefore, in this study, we quantified the “additive effect” of rhubarb chemical groups. Through specific values, we can see that combined anthraquinones and flavanols and their polymers may be the universal characters of the multi-functional properties of rhubarb, reflecting the correlation among multiple efficacies to some extent. For example, chrysophanol glucosides rank the 18th, 4th, 4th, 8th and 10th severally in E1 ~ 5. Their aglycones, namely free anthraquinones, possess strong bacteriostasis and notably affect the activation of lipid inflammatory mediators [46]. There are currently many evidences that rhubarb anthraquinones can treat constipation, ischemic lesions [47], sepsis [48] and other inflammation. Especially now in the suppression of hepatobiliary diseases [49, 50], Kehuanglidan Capsule has completed the treatment of chronic viral hepatitis jaundice in the drug clinical trial registration and information public platform (www.chinadrugtrials.org.cn), and its dominant ingredients are the rhubarb anthraquinones. Some literature speculated that these anthraquinone glycosides may revert to free type ones through metabolic transformation in vivo to exert various pharmacological actions. In addition, flavanols such as Cianidanol and (-)-Epicatechin rank in the top 10 of E1 ~ 5. Catechins contribute to improve metabolic changes caused by a high-fat diet [51], and can maintain normal blood circulation by reducing the adhesion of platelets [52] and efficaciously shorten icteric period by lowering serum bilirubin levels [53]. Procyanidins are the polymers of flavanols. They also have great contributions for E1, E2, E3 and E5, showing protective effects on the digestive system for the treatment of gastrointestinal diseases, cholecystitis, constipation or diarrhea, etc. In particular, oligomeric procyanidins are the most in the top 10 of E2, which are the internationally recognized natural antioxidant to scavenge free radicals.

With respect to individual characters of different efficacies, stilbene glycosides are important to E2 ~ 5 that are closely linked to anti-bacteria, antiphlogosis and other aspects [54]. Stilbene compounds are always a momentous part of rhubarb chiefly for anti-hyperlipidemia and antioxidation [49]. Resveratrol glucosides are the representative ranking high in these efficacies, which can alleviate LPS stimulation by inhibiting TNF-α and macrophages from producing NO [55]. Sennosides belonging to anthranone dimers are responsible not only for defaecation (E1), but also for detoxifying to treat bacillary dysentery, epidemic hemorrhagic fever (E3) and postpartum milk return in clinic (E4). Free anthraquinones, mainly Emodin, Physcion, Rhein or their derivates, act directly in E1 and E5. Chromones appear in the top 10 of E1 and E2, which have been proven to remove cholesterol, inflammation and so on [49]. A kind of gallotannins ranks the 9th in E3. Gallotannins are generally characterized by potent antivirus, astringency and hemostasis [49]. E3 of rhubarb is related to clinical blood loss syndrome, and grey relational analysis has attested to a big impact of tannins and anthraquinones on the hemostatic function [56]. butyrylbenzenes and their glycosides may play a certain protective role on the cardiovascular system (E4).

To sum up, we focused on those components whose contribution over 0.01 and the top 10 components for different efficacies of rhubarb. Furthermore, the contributions of different component groups in rhubarb to efficacies were scientifically understood. The discovery of a novel drug advocated a holistic network-oriented approach [57]. Based on traditional medicine philosophies in the view of holism, our findings may be preferable for systemic research of rhubarb, providing the basis for further studies on its mechanisms of action, compatibility of prescription, precise clinical applications, and the like. We will continue to carry out subsequent work for in-depth experimental validation on the discoverable effective constituents ulteriorly as well.

## Conclusions

This study proposed a novel strategy to fuzzily identify bioactive components for different efficacies of rhubarb by BP neural network association analysis of UPLC-Q-TOF/MS^E^ and integrated effects. Based on the fuzzy chemical identification, we grouped more than 100 components into ten categories, and then established three mouse models to explore the five efficacies recorded in Chinese Pharmacopoeia. It suggested that combined anthraquinones and flavanols and their polymers were the universal features of the multi-functional properties of rhubarb while stilbene glycosides, anthranones and their dimers, free anthraquinones, chromones, gallic acid and gallotannins, and butyrylbenzenes and their glycosides contributed to the individuality of rhubarb’s efficacies. These results will provide scientific evidence to support the extensive use of rhubarb in clinical applications as well as the further development of several products based on this medicinal herb.

## Supplementary Information

**Additional file 1: Table S1.** Information of 28 chemical references of rhubarb. **Table S2**. The full-spectrum information database of chemical compounds of rhubarb. **Table S3.** Relative contents of 108 chemical components from rhubarb samples (n = 3). **Table S4.** The scoring coefficient matrix of E1 (10 indicators, KMO = 0.548 & significance of Bartlett’s Test = 0, initial eigenvalue > 0.8, on behalf of 80.834% raw data). **Table S5.** The scoring coefficient matrix of E2 (13 indicators, KMO = 0.562 & significance of Bartlett’s Test = 0, initial eigenvalue > 0.7, on behalf of 82.112% raw data). **Table S6.** The scoring coefficient matrix of E3 (16 indicators, KMO = 0.646 & significance of Bartlett’s Test = 0, initial eigenvalue > 0.7, on behalf of 78.44% raw data). **Table S7.** The scoring coefficient matrix of E4 (10 indicators excluding APTT with the worst correlation, KMO = 0.55 & significance of Bartlett’s Test = 0, initial eigenvalue > 0.65, on behalf of 84.936% raw data). **Table S8.** The scoring coefficient matrix of E5 (15 indicators, KMO = 0.807 & significance of Bartlett’s Test = 0, initial eigenvalue > 0.65, on behalf of 87.993% raw data). **Table S9.** Normal distribution test parameters of the integration E1 in each group. **Table S10.** Normal distribution test parameters of the integration E2 in each group. **Table S11.** Normal distribution test parameters of the integration E3 in each group. **Table S12.** Normal distribution test parameters of the integration E4 in each group. **Table S13.** Normal distribution test parameters of the integration E5 in each group. **Table S14.** The predicted (P), true (T) and their relative error (R) values of test samples in single, double and three hidden layers. <Emphasis Type="Bold">Table S15.</Emphasis> The predicted (P), true (T) and their relative error (R) values of test samples in a different number of neurons per hidden layer. <Emphasis Type="Bold">Table S16.</Emphasis> The predicted (P), true (T) and their relative error (R) values of test samples in different learning rates. <Emphasis Type="Bold">Table S17.</Emphasis> Decision weights over 0.01 of rhubarb components ranking by their total weights (the ordinal number after the values represents the top 10 order in each effect respectively; weights < 0.01 are ignored by “-”). <Emphasis Type="Bold">Table S18.</Emphasis> Identification of the top 10 components in total decision weights of five effects. <Emphasis Type="Bold">Table S19.</Emphasis> Identification of the top 10 components in E1.**Table S20.** Identification of the top 10 components in E2. **Table S21.** Identification of the top 10 components in E3. **Table S22.** Identification of the top 10 components in E4. **Table S23.** Identification of the top 10 components in E5.**Figure S1.** The LC-MS profiling related to different extraction methods: effect of extraction solvents (A), extraction time (B), extraction sequence and incorporation (C) on the difference of composition changes. **Figure S2.** E1 indexes: defecation characteristics of first black stool time (A1), the number of black stools (A2), fecal weights within 12 h (A3); colonic content weights (B1), organ coefficients of colon (B2) and stomach (B3) after dissection; MTL (C1), SS (C2), VIP (C3) in mouse serum; AchE (D) in duodenal tissues. (n = 8, mean ± SD). **Figure S3.** E2 indexes: TG (A) in mouse serum; Na+-K+-ATPase (B1), TNF-α (B2), IL-1β (B3) in duodenal tissues; representative exposed protein bands (C) and corresponding grayscale ratios of p-NF-κB p65 (C1), NF-κB p65 (C2), p-p38 (C3), p38 (C4), p-ERK (C5), ERK (C6), p-JNK (C7), JNK (C8), TLR4 (C9) in the colon of each group. (n = 8, mean ± SD). **Figure S4.** E3 indexes: the second measurement of anal temperatures (A); HSP-70 (B1), SOD (B2), NO (B3), TNF-α (B4), IL-1β (B5), IL-6 (B6) in mouse serum; representative exposed protein bands (C) and corresponding grayscale ratios of p-NF-κB p65 (C1), NF-κB p65 (C2), p-p38 (C3), p38 (C4), p-ERK (C5), ERK (C6), p-JNK (C7), JNK (C8), TLR4 (C9) in the colon of each group. (n = 6, mean ± SD). **Figure S5.** E4 indexes: TT (A1), PT (A2), APTT (A3), FIB (A4) in mouse plasma; TXB2 (B1), 6-keto-PGF1α (B2), ratios of TXB2 to 6-keto-PGF1α (B3), PGE2 (B4), ET-1 (B5), Mg2+ (B6), Ca2+ (B7) in the serum. (n = 6, mean ± SD). **Figure S6.** Cholestatic manifestations of weight changes (A) before and after ANIT modeling, representative serum color (B), livers and gallbladders (C) after morphological dissection in each mouse group. (n = 8, mean ± SD). **Figure S7.** E5 indexes: organ coefficients of liver (A1) and gallbladder (A2); T-SOD (B1), MDA (B2), GSH (B3), Fe3+ (B4) in the homogenate of liver tissues; ALT (C1), AST (C2), ALP (C3), Fe3+ (C4), GST (C5), GGT (C6), TBIL (C7), DBIL (C8), TBA (C9) in mouse serum. (n = 8, mean ± SD). **Figure S8**. The running program of BP neural network (a) including four parts: the network structure, algorithms, training progress and plots; running effect diagrams of E2 (b), E3 (c), E4 (d) and E5 (e) including the neural network performance (1), training state (2), regression analysis (3), comparison between the predicted and true values (4: the line is the prediction curve and “*” denotes the true value of each group).

## Data Availability

The research data generated from this study are included in the article and additional files.

## Abbreviations

AchE: Acetyl cholinesterase
Adr: Adrenalin hydrochloride
ALP: Alkaline phosphatase
ALT: Glutamic-pyruvic transaminase
ANIT: α-Naphthyl isothiocyanate
APTT: Activated partial thromboplastin time
AST: Glutamic-oxalacetic transaminase
BP: Back propagation
BPI: Base peak intensity
Ca^2+^: Calcium ion
CAS: Chemical abstracts service
DBIL: Direct bilirubin
DMSO: Dimethyl sulfoxide
E1: Removing accumulation with purgation
E2: Clearing heat and purging fire
E3: Cooling blood and detoxifying
E4: Eliminating blood stasis to remove obstruction in channels
E5: Disinhibiting dampness and removing jaundice
ELISA: Enzyme-linked immunosorbent assay
ET-1: Endothelin-1
EW: Ethanol–water
Fe^3+^: Ferric ion
FIB: Fibrinogen
GAPDH: Glyceraldehyde-3-phosphate dehydrogenase
GGT: γ-Glutamyl transpeptidase
GSH: Glutathione
GST: Glutathione sulfhydryl transferase
HSP-70: Heat shock protein-70
IL: Interleukin
L: Long time
LPS: Lipopolysaccharide
MDA: Malondialdehyde
Mg^2+^: Magnesium ion
MTL: Motilin
Na^+^-K^+^-ATPase: Sodium potassium pump
NO: Nitric oxideNS: normal saline
PCA: Principal component analysis
(p-)ERK: (Phosphorylated-)extracellular regulated protein kinase
PG: Prostaglandin
(p-)JNK: (Phosphorylated-)c-Jun N-terminal kinase
(p-)NF-κB p65: (Phosphorylated-)nuclear factor-kappaB protein65
(p-)p38: (Phosphorylated-)protein38
PT: Prothrombin time
QC: Quality control
RT: Retention time
S: Short time
SD: Standard deviation
SS: Somatostatin
TBA: Total bile acid
TBIL: Total bilirubin
TCM: Traditional Chinese medicine
TG: Triglyceride
TLR4: Toll-like receptor4
TNF: Tumor necrosis factor
(T-)SOD: (Total-)superoxide dismutase
TT: Thrombin time
TXB_2_: Thromboxane B_2_
UPLC-Q-TOF/MS^E^: Ultra-performance liquid chromatography/quadrupole time-of-flight mass spectrometry for every data
UV: Ultraviolet
VIP: Vasoactive intestinal peptide
