# Supplementary material for: Fuzzy identification of bioactive components for different efficacies of rhubarb by the back propagation neural network association analysis of UPLC-Q-TOF/MSE and integrated effects
Source: Chin Med. 2022 Apr 26;17:50. doi: 10.1186/s13020-022-00612-9 (PMC9040240; doi:10.1186/s13020-022-00612-9)
Supplement: Supplementary file 1 — Additional file 1: Table S1. Information of 28 chemical references of rhubarb. Table S2. The full-spectrum information database of chemical compounds of rhubarb. Table S3. Relative contents of 108 chemical components from rhubarb samples (n = 3). Table S4. The scoring coefficient matrix of E1 (10 indicators, KMO = 0.548 & significance of Bartlett’s Test = 0, initial eigenvalue > 0.8, on behalf of 80.834% raw data). Table S5. The scoring coefficient matrix of E2 (13 indicators, KMO = 0.562 & significance of Bartlett’s Test = 0, initial eigenvalue > 0.7, on behalf of 82.112% raw data). Table S6. The scoring coefficient matrix of E3 (16 indicators, KMO = 0.646 & significance of Bartlett’s Test = 0, initial eigenvalue > 0.7, on behalf of 78.44% raw data). Table S7. The scoring coefficient matrix of E4 (10 indicators excluding APTT with the worst correlation, KMO = 0.55 & significance of Bartlett’s Test = 0, initial eigenvalue > 0.65, on behalf of 84.936% raw data). Table S8. The scoring coefficient matrix of E5 (15 indicators, KMO = 0.807 & significance of Bartlett’s Test = 0, initial eigenvalue > 0.65, on behalf of 87.993% raw data). Table S9. Normal distribution test parameters of the integration E1 in each group. Table S10. Normal distribution test parameters of the integration E2 in each group. Table S11. Normal distribution test parameters of the integration E3 in each group. Table S12. Normal distribution test parameters of the integration E4 in each group. Table S13. Normal distribution test parameters of the integration E5 in each group. Table S14. The predicted (P), true (T) and their relative error (R) values of test samples in single, double and three hidden layers. Table S15. The predicted (P), true (T) and their relative error (R) values of test samples in a different number of neurons per hidden layer. Table S16. The predicted (P), true (T) and their relative error (R) values of test samples [file 13020_2022_612_MOESM1_ESM.doc]

Contents

[Contents 1](#__RefHeading___Toc98011338)

[Materials and methods 3](#__RefHeading___Toc98011339)

[Table S1 4](#__RefHeading___Toc98011340)

[Table S2 6](#__RefHeading___Toc98011341)

[**References 48**](#__RefHeading___Toc98011342)

[Table S3 51](#__RefHeading___Toc98011343)

[Table S4 55](#__RefHeading___Toc98011344)

[Table S5 56](#__RefHeading___Toc98011345)

[Table S6 57](#__RefHeading___Toc98011346)

[Table S7 58](#__RefHeading___Toc98011347)

[Table S8 59](#__RefHeading___Toc98011348)

[Table S9 60](#__RefHeading___Toc98011349)

[Table S10 61](#__RefHeading___Toc98011350)

[Table S11 62](#__RefHeading___Toc98011351)

[Table S12 63](#__RefHeading___Toc98011352)

[Table S13 64](#__RefHeading___Toc98011353)

[Table S14 65](#__RefHeading___Toc98011354)

[Table S15 66](#__RefHeading___Toc98011355)

[Table S16 68](#__RefHeading___Toc98011356)

[Table S17 70](#__RefHeading___Toc98011357)

[Table S18 73](#__RefHeading___Toc98011358)

[Table S19 74](#__RefHeading___Toc98011359)

[Table S20 75](#__RefHeading___Toc98011360)

[Table S21 76](#__RefHeading___Toc98011361)

[Table S22 77](#__RefHeading___Toc98011362)

[Table S23 78](#__RefHeading___Toc98011363)

[Figure S1 79](#__RefHeading___Toc98011364)

[Figure S2 80](#__RefHeading___Toc98011365)

[Figure S3 81](#__RefHeading___Toc98011366)

[Figure S4 82](#__RefHeading___Toc98011367)

[Figure S5 83](#__RefHeading___Toc98011368)

[Figure S6 84](#__RefHeading___Toc98011369)

[Figure S7 85](#__RefHeading___Toc98011370)

[Figure S8 86](#__RefHeading___Toc98011371)

**Materials and methods**

1. Table S1, Figure S1 and Table S2 were listed below.
2. Antibodies: anti-p-NF-κB p65 (Abcam, GR3202914-2), anti-NF-κB p65, anti-TLR4 (Santa Cruz Biotechnology, Inc., #F2912, #A2714), anti-p-p38, anti-p-ERK, anti-p-JNK (Ruiying Biotechnology Co., Ltd., RLP0718, RLP0101, RLP0157), anti-p38 (Cell Signaling Technology, Inc., 8690S), anti-ERK, anti-JNK (Proteintech Group, Inc., 16443-1-AP, 51151-1-AP), anti-GAPDH (GeneTex, Inc., 41577), and Goat anti-rabbit (#ABL3012)/mouse (#ABL3032) IgG secondary antibodies HRP conjugated were from AbSci, Llc.
3. Western blot: We exactly weighed about 0.5 cm of colon tissues, added with RIPA lysis buffer 20 μL/mg and steel balls to grind for 3–5 min. After putting colon homogenates standing on the ice for 30 min, we centrifuged them at 12000 r/min for 10 min at 4 °C and then pipetted the supernatants. On the one hand, we quantified proteins of the supernatants by the instructions of BCA protein assay kit to decide the loading quantity of samples. On the other hand, we prepared samples by adding 30 μL of the buffer solution into 120 μL of each supernatant, boiled them for 10 min and stored them at −20 °C for use after reaching room temperature. On the next day, we followed the normative operating procedure of Western blotting, such as loading samples on the colloid, running electrophoresis, transferring membrane, and combining samples with primary antibodies (anti-NF-κB p65/p-NF-κB p65/p38/p-p38/ERK/p-ERK/JNK/p-JNK/TLR4/GAPDH) after blocking and placing them at 4°C for overnight. On the third day, we mainly incubated samples with goat anti-rabbit IgG secondary antibody HRP conjugated after warming the membranes and used ECL luminous fluid for color development. Finally, the membranes with samples would be scanned by gel imager. We picked the best exposed protein bands and used Image Lab 3.0 software for gray analysis to calculate the grayscale statistics of each group of bands.

**Table S1**

Information of 28 chemical references of rhubarb.

| No. | Name | CAS | Chemical structure | | Exact mass |
| --- | --- | --- | --- | --- | --- |
| 1 | Chrysophanol | 481-74-3 |  | R_1_=R_3_=R_4_=H R_2_=CH_3_ | 254.0579 |
| 2 | Chrysophanol 1-*O*-*β*-D-glucoside | 4839-60-5 |  | R_1_=Glc R_2_=CH_3_ R_3_=R_4_=H | 416.1107 |
| 3 | Chrysophanol 8-*O*-*β*-D-glucoside | 13241-28-6 |  | R_1_=R_3_=H R_2_=CH_3_ R_4_=Glc | 416.1107 |
| 4 | Emodin | 518-82-1 |  | R_1_=R_4_=H  R_2_=CH_3_  R_3_=OH | 270.0528 |
| 5 | Emodin 1-*O*-*β*-D-glucoside | 38840-23-2 |  | R_1_=Glc R_2_=CH_3_ R_3_=OH  R_4_=H | 432.1056 |
| 6 | Emodin 8-*O*-*β*-D-glucoside | 23313-21-5 |  | R_1_=H  R_2_=CH_3_  R_3_=OH R_4_=Glc | 432.1056 |
| 7 | Physcion | 521-61-9 |  | R_1_=R_4_=H R_2_=CH_3_ R_3_=OCH_3_ | 284.0685 |
| 8 | Physcion 1-*O*-*β*-D-glucoside | 26296-54-8 |  | R_1_=Glc R_2_=CH_3_ R_3_=OCH_3_ R_4_=H | 446.1213 |
| 9 | Physcion 8-*O*-*β*-D-glucoside | 23451-01-6 |  | R_1_=H R_2_=CH_3_ R_3_=OCH_3_ R_4_=Glc | 446.1213 |
| 10 | Aloe-emodin | 481-72-1 |  | R_1_=R_3_=R_4_=H R_2_=CH_2_OH | 270.0528 |
| 11 | Aloe-emodin 8-*O*-*β*-D-glucoside | 33037-46-6 |  | R_1_=R_3_=H R_2_=CH_2_OH R_4_=Glc | 432.1056 |
| 12 | Rhein | 478-43-3 |  | R_1_=R_3_=R_4_=H R_2_=COOH | 284.0321 |
| 13 | Rhein 8-*O*-*β*-D-glucoside | 34298-86-7 |  | R_1_=R_3_=H R_2_=COOH R_4_=Glc | 446.0849 |
| 14 | Sennoside A | 81-27-6 |  | R=COOH (10,10'-*trans*) | 862.1956 |
| 15 | Sennoside B | 128-57-4 |  | R=COOH (10,10'-*cis*) | 862.1956 |
| 16 | Sennoside C | 37271-16-2 |  | R=CH_2_OH (10,10'-*trans*) | 848.2164 |
| 17 | Cianidanol | 154-23-4 |  | R=H (3*S*) | 290.0790 |
| 18 | (-)-Epicatechin | 490-46-0 |  | R=H (3*R*) | 290.0790 |
| 19 | (-)-Epicatechin gallate | 1257-08-5 |  | R=G (3*R*) | 442.0900 |
| 20 | Procyanidin B2 | 29106-49-8 |  | | 578.1424 |
| 21 | Gallic acid | 149-91-7 |  | | 170.0215 |
| 22 | Resveratroloside | 38963-95-0 |  | R_1_=H R_2_=Glc | 390.1315 |
| 23 | Rhapontigenin | 500-65-2 |  | R_1_=OH R_2_=CH_3_ | 258.0892 |
| 24 | Torachrysone 8-*O*-glucoside | 64032-49-1 |  | | 408.1420 |
| 25 | 4-(4-Hydroxyphenyl)-2-butanone | 5471-51-2 |  | R=H | 164.0837 |
| 26 | Raspberryketone glucoside | 38963-94-9 |  | R=Glc | 326.1365 |
| 27 | Lindleyin | 59282-56-3 |  | R=Glc^6^→O-G | 478.1475 |
| 28 | 5-Acetyl-7-hydroxy-2-methyl-chromone | 94356-33-9 |  | | 218.0579 |

**Table S2**

The full-spectrum information database of chemical compounds of rhubarb.

| No. | Compound name | CAS | Structure | Exact mass (Formula) | MS | UV | PubChem CID/ Refer |
| --- | --- | --- | --- | --- | --- | --- | --- |
| 1. Anthraquinones | | | | | | | |
| 1 | 9,10-Anthraquinone | 84-65-1 | R_1_=R_2_=R_3_=R_4_=R_5_=R_6_=R_7_=R_8_=H | 208.0524 (C_14_H_8_O_2_) | EI^+^: 208, 180, 76/ 152 | 252, 278, 330 (log E = 4.7, 4.1, 3.7) | 6780/ [1](#_ENREF_1) |
| 2 | Alizarin | 72-48-0 | R_1_=R_2_=OH R_3_=R_4_=R_5_=R_6_=R_7_=R_8_=H | 240.0423 (C_14_H_8_O_4_) | ESI^-^: 239.035 [M-H]^-^, 211.04, 210.032/ 238.025; EI^+^: 240, 239, 241 |  | 6293/ [1](#_ENREF_1) |
| 3 | 1,2-Dimethoxy-9,10-anthraquinone | 6003-12-9 | R_1_=R_2_=OCH_3_ R_3_=R_4_=R_5_=R_6_=R_7_=R_8_=H | 268.0736 (C_16_H_12_O_4_) |  |  | 4217712/ [1](#_ENREF_1) |
| 4 | 1,4-Dihydroxy-9,10-anthraquinone | 81-64-1 | R_1_=R_4_=OH R_2_=R_3_=R_5_=R_6_=R_7_=R_8_=H | 240.0423 (C_14_H_8_O_4_) | ESI^-^: 239.03 [M-H]^-^, 223.028, 240.042/ 211.039; EI^+^: 240, 241, 239 | 330, 470, 520 (Log E = 3.5, 3.9, 3.7) | 6688/ [1](#_ENREF_1) |
| 5 | 1,4,5-Trihydroxy-2-methoxy-3-methylanthracene-9,10-dione |  | R_1_=R_4_=R_5_=OH R_2_=OCH_3_ R_3_=CH_3_ R_6_=R_7_=R_8_=H | 300.0634 (C_16_H_12_O_6_) |  |  | TCMSP |
| 6 | 1,4-Dihydroxy-2-methoxy-3-methyl-5-[(2*S*,3*R*,4*S*,5*S*,6*R*)-3,4,5-trihydroxy-6-(hydroxymethyl)oxan-2-yl]oxyanthracene-9,10-dione | 959571-86-9 | R_1_=R_4_=OH R_2_=OCH_3_ R_3_=CH_3_ R_5_=O-Glc R_6_=R_7_=R_8_=H | 462.1162 (C_22_H_22_O_11_) |  |  | 11968838/ TCMSP |
| 7 | 1,5-Dihydroxy-9,10-anthraquinone | 117-12-4 | R_1_=R_5_=OH R_2_=R_3_=R_4_=R_6_=R_7_=R_8_=H | 240.0423 (C_14_H_8_O_4_) | ESI^+^: 241.049 [M+H]^+^, 213.055, 121.028, 185.06; EI^+^: 240, 241, 184/ 92 | 416 | 8328/ |
| 8 | 2,6-Dihydroxy-9,10-anthraquinone | 84-60-6 | R_1_=R_3_=R_4_=R_5_=R_7_=R_8_=H R_2_=R_6_=OH | 240.0423 (C_14_H_8_O_4_) | ESI^-^: 239.035 [M-H]^-^, 211.04, 195.045; EI^+^: 240, 155, 184/ 212 |  | 6776/ [1](#_ENREF_1) |
| 9 | 2,6-Dimethoxy-9,10-anthraquinone | 963-96-2 | R_1_=R_3_=R_4_=R_5_=R_7_=R_8_=H R_2_=R_6_=OCH_3_ | 268.0736 (C_16_H_12_O_4_) |  |  | 4589998/ [1](#_ENREF_1) |
| 10 | Danthron | 117-10-2 | R_1_=R_8_=OH R_2_=R_3_=R_4_=R_5_=R_6_=R_7_=H | 240.0423 (C_14_H_8_O_4_) | ESI^-^: 239.035 [M-H]^-^, 240.038, 241.04; EI^+^: 240, 184, 212; ESI^+^(IT): 241.049 [M+H]^+^, 222.1, 213, 185 | 430, 250 (log E = 4.35, 4.6) | 2950/ [1](#_ENREF_1) |
| 11 | Chrysophanol | 481-74-3 | R_1_=R_8_=OH R_2_=R_4_=R_5_=R_6_=R_7_=H R_3_=CH_3_ | 254.0579 (C_15_H_10_O_4_) | ESI^+^: 255.065 [M+H]^+^, 176.987, 177.031, 177.069/ 173.078; EI^+^: 254, 255, 226 | 226, 256, 278, 288, 436 (epsilon X10-3 = 41, 28, 14, 14, 11.8) | 10208 |
| 12 | 1-Hydroxy-8-methoxy-3-methyl-9,10-anthraquinone | 3300-25-2 | R_1_=OH R_2_=R_4_=R_5_=R_6_=R_7_=H R_3_=CH_3_ R_8_=OCH_3_ | 268.0736 (C_16_H_12_O_4_) | ESI^+^: 268 [M]^+^, 250, 239, 222, 149 |  | 11300144/ [3](#_ENREF_3) |
| 13 | Chrysophanol 1-*O*-*β*-D-glucoside | 4839-60-5 | R_1_=O-Glc R_2_=R_4_=R_5_=R_6_=R_7_=H R_3_=CH_3_ R_8_=OH | 416.1107 (C_21_H_20_O_9_) | ESI^+^: 439.07 [M+Na]+/ 416.04 [M]+/ 417.07 [M+H]^+^, 415.04, 418.04 | 220, 260, 285 | 6324923 |
| 14 | Chrysophanol 8-*O*-*β*-D-glucoside | 13241-28-6 | R_1_=OH R_2_=R_4_=R_5_=R_6_=R_7_=H R_3_=CH_3_ R_8_=O-Glc | 416.1107 (C_21_H_20_O_9_) | ESI^+^: 416 [M]^+^, 254 | 407, 256, 223, 198, 192 | 442731/ |
| 15 | Chrysophanol 8-*O*-*β*-D-(6'-*O*-acetyl)-glucoside | 440087-83-2 | R_1_=OH R_2_=R_4_=R_5_=R_6_=R_7_=H R_3_=CH_3_ R_8_=O-Glc^6^→O-COCH_3_ | 458.1213 (C_23_H_22_O_10_) |  |  |  |
| 16 | Chrysophanol 8-*O*-*β*-D-(6'-*O*-galloyl)-glucoside | 266997-57-3 | R_1_=OH R_2_=R_4_=R_5_=R_6_=R_7_=H R_3_=CH_3_ R_8_=O-Glc^6^→O-G | 568.1217 (C_28_H_24_O_13_) | ESI^+^: 591 [M+Na]+/ 569.1 [M+H]^+^, 153, 256, 255, 315, 570.1, 86.1, 571.1 | 220, 260, 284, 409 (log E = 4.58, 4.32, 4.17, 3.79) | 78384671/ 5315852/ |
| 17 | Chrysophanol 8-*O*-*β*-D-(6'-*O*-malonyl)-glucoside | 205107-13-7 | R_1_=OH R_2_=R_4_=R_5_=R_6_=R_7_=H R_3_=CH_3_ R_8_=O-Glc^6^→O-COCH_2_COOH | 502.1111 (C_24_H_22_O_12_) | ESI^+^: 502 [M]^+^, 254, 226 | 221, 256, 282 (log E = 4.1, 2.2, 1.9) | 45359837/ [9](#_ENREF_9) |
| 18 | Emodin | 518-82-1 | R_1_=R_6_=R_8_=OH R_2_=R_4_=R_5_=R_7_=H R_3_=CH_3_ | 270.0528 (C_15_H_10_O_5_) | ESI^+^: 271.06 [M+H]^+^, 197.06, 225.051/ 169.064; EI^+^: 270, 271, 242; ESI^+^(IT): 271, 239, 270 | 222, 252, 265, 289, 437 (log E = 4.55, 4.26, 4.27, 4.34, 4.1) | 3220 |
| 19 | Ω-Hydroxyemodin/ Citreorosein | 481-73-2 | R_1_=R_6_=R_8_=OH R_2_=R_4_=R_5_=R_7_=H R_3_=CH_2_OH | 286.0477 (C_15_H_10_O_6_) | ESI^+^: 287.054 [M+H]^+^, 241.047, 269.041; ESI^-^: 285.04 [M-H]^-^, 286.044, 285.189 |  | 361512 |
| 20 | Revandchinone 3 | 00-00-6363 | R_1_=R_8_=OH R_2_=R_4_=R_5_=R_7_=H R_3_=CH_3_ R_6_=O(CH_2_)_21_CH_3_ | 578.3971 (C_37_H_54_O_5_) | ESI^+^: 601 [M+Na]^+^ | 440, 285, 280 | 5320941/ [10](#_ENREF_10) |
| 21 | Rhababerone/ Isoemodin | 476-62-0 | R_1_=R_4_=R_6_=R_7_=H R_2_=R_5_=R_8_=OH R_3_=CH_3_ | 270.0528 (C_15_H_10_O_5_) |  |  | 12310964/ [1](#_ENREF_1) |
| 22 | 1,6,8-Trimethoxy-3-methyl-9,10-anthraquinone | 6414-42-2 | R_1_=R_6_=R_8_=OCH_3_ R_2_=R_4_=R_5_=R_7_=H R_3_=CH_3_ | 312.0998 (C_18_H_16_O_5_) |  |  | 7330510/ [1](#_ENREF_1) |
| 23 | Emodin 1-*O*-*β*-D-glucoside | 38840-23-2/ 86451-01-6 | R_1_=O-Glc R_2_=R_4_=R_5_=R_7_=H R_3_=CH_3_ R_6_=R_8_=OH | 432.1056 (C_21_H_20_O_10_) | ESI^-^: 431 [M-H]^-^, 256, 207 | 253, 287, 425 (log E = 4.34, 4.36, 3.92) | 5319333/ |
| 24 | Emodin 6-*O*-*β*-D-glucoside | 34298-85-6 | R_1_=R_8_=OH R_2_=R_4_=R_5_=R_7_=H R_3_=CH_3_ R_6_=O-Glc | 432.1056 (C_21_H_20_O_10_) |  |  | 5317038 |
| 25 | Emodin 8-*O*-*β*-D-glucoside | 23313-21-5 | R_1_=R_6_=OH R_2_=R_4_=R_5_=R_7_=H R_3_=CH_3_ R_8_=O-Glc | 432.1056 (C_21_H_20_O_10_) | ESI^-^: 431.1 [M-H]^-^, 269.045, 225.055, 270.05 | 280 | 99649/ |
| 26 | Emodin 8-*O*-*β*-D-gentiobioside | 66466-22-6 | R_1_=R_6_=OH R_2_=R_4_=R_5_=R_7_=H R_3_=CH_3_ R_8_=O-Glc^6^→O→^1^Glc | 594.1585 (C_27_H_30_O_15_) | ESI^+^: 595 [M+H]^+^ |  | 71587230/ [15](#_ENREF_15) |
| 27 | Emodin 8-*O*-*β*-D-glucopyranosyl-6-*O*-sulfate | 600153-62-6 | R_1_=OH R_2_=R_4_=R_5_=R_7_=H R_3_=CH_3_ R_6_=OSO_3_H R_8_=O-Glc | 512.0625 (C_21_H_20_O_13_S) | ESI^-^: 511 [M-H]^-^, 431, 269, 349 | 326, 410 | [16](#_ENREF_16) |
| 28 | Physcion | 521-61-9 | R_1_=R_8_=OH R_2_=R_4_=R_5_=R_7_=H R_3_=CH_3_ R_6_=OCH_3_ | 284.0685 (C_16_H_12_O_5_) | ESI^+^: 285.077 [M+H]^+^, 270.054, 242.058; ESI^-^: 283.061 [M-H]^-^, 283.264, 284.268; EI^+^: 284, 285, 255/ 128 | 220, 253, 263.8, 292, 433 | 10639/ [17](#_ENREF_17) |
| 29 | 1,8-Dihydroxy-3-methoxy-2,6-dimethylanthracene-9,10-dione |  | R_1_=R_8_=OH R_2_=R_4_=R_5_=H R_3_=R_7_=CH_3_ R_6_=OCH_3_ | 298.0841 (C_17_H_14_O_5_) |  |  | 21770756/ TCMSP |
| 30 | Physcion 1-*O*-*β*-D-glucoside | 26296-54-8 | R_1_=O-Glc R_2_=R_4_=R_5_=R_7_=H R_3_=CH_3_ R_6_=OCH_3_ R_8_=OH | 446.1213 (C_22_H_22_O_10_) | ESI^+^: 469.11 [M+Na]^+^, 475.33, 437.19, 285; ESI^-^: 283 | 283, 422 | 5319323/ [1](#_ENREF_1) |
| 31 | Physcion 8-*O*-*β*-D-glucoside | 23451-01-6/ 29013-18-1/ 1329-27-7 | R_1_=OH R_2_=R_4_=R_5_=R_7_=H R_3_=CH_3_ R_6_=OCH_3_ R_8_=O-Glc | 446.1213 (C_22_H_22_O_10_) | ESI^+^: 469.2 [M+Na]^+^, 437.3, 481.5 | 205, 256, 270 | 168938/ |
| 32 | Physcion 8-*O*-*β*-D-gentiobioside | 84268-38-2 | R_1_=OH R_2_=R_4_=R_5_=R_7_=H R_3_=CH_3_ R_6_=OCH_3_ R_8_=O-Glc^6^→O→^1^Glc | 608.1741 (C_28_H_32_O_15_) |  |  | 442762 |
| 33 | 1,6-Dimethoxy-3-methyl-8-[3,4,5-trimethoxy-6-(methoxymethyl)oxan-2-yl]oxyanthracene-9,10-dione | 85933-17-1 | R_1_=R_6_=OCH_3_ R_2_=R_4_=R_5_=R_7_=H R_3_=CH_3_ R_8_=O | 516.1995 (C_27_H_32_O_10_) |  |  | 44297849/ [1](#_ENREF_1) |
| 34 | Aloe-emodin | 481-72-1 | R_1_=R_8_=OH R_2_=R_4_=R_5_=R_6_=R_7_=H R_3_=CH_2_OH | 270.0528 (C_15_H_10_O_5_) | ESI^+^: 271.06 [M+H]^+^, 153.018, 119.047/ 271.106, 271.02; EI^+^: 270, 241, 121 | 226, 256, 288 (log E = 4.5, 4.1, 3.8) | 10207/ [9](#_ENREF_9) |
| 35 | 6-Methyl-aloe-emodin | 873431-39-1 | R_1_=R_8_=OH R_2_=R_4_=R_5_=R_7_=H R_3_=CH_2_OH R_6_=CH_3_ | 284.0685 (C_16_H_12_O_5_) | ESI^+^: 284 [M]^+^, 267, 270, 228, 121, 107, 256 | 221, 252, 266, 290, 420 (log E = 2.51, 4.81, 3.4, 1.6, 3.6) | 71413104/ [19](#_ENREF_19) |
| 36 | Aloe-emodin 1-*O*-*β*-D-glucoside | 266997-58-4 | R_1_=O-Glc R_2_=R_4_=R_5_=R_6_=R_7_=H R_3_=CH_2_OH R_8_=OH | 432.1056 (C_21_H_20_O_10_) | ESI^+^: 455 [M+Na]^+^ | 222, 257, 282, 407 (log E = 4.55, 4.37, 4.06, 3.84) | 44339807/ [8](#_ENREF_8) |
| 37 | Aloe-emodin 8-*O*-*β*-D-glucoside | 33037-46-6 | R_1_=OH R_2_=R_4_=R_5_=R_6_=R_7_=H R_3_=CH_2_OH R_8_=O-Glc | 432.1056 (C_21_H_20_O_10_) | ESI^+^: 432 [M]^+^, 270 | 222, 253, 283 (log E = 4.2, 3.1, 1.2) | 5317644/ [9](#_ENREF_9) |
| 38 | Aloe-emodin Ω-*O*-*β*-D-glucoside | 29010-56-8 | R_1_=R_8_=OH R_2_=R_4_=R_5_=R_6_=R_7_=H R_3_=CH_2_O-Glc | 432.1056 (C_21_H_20_O_10_) |  | 226, 256, 433 (log E = 4.72, 4.4, 4.05) | 147295/ [11](#_ENREF_11) |
| 39 | Aloe-emodin 1,8-di-*O*-*β*-D-glucoside | 26289-44-1 | R_1_=R_8_=O-Glc R_2_=R_4_=R_5_=R_6_=R_7_=H R_3_=CH_2_OH | 594.1585 (C_27_H_30_O_15_) |  |  | 73157752 |
| 40 | Rhein | 478-43-3 | R_1_=R_8_=OH R_2_=R_4_=R_5_=R_6_=R_7_=H R_3_=COOH | 284.0321 (C_15_H_8_O_6_) | ESI^+^: 285.039 [M+H]^+^, 155.048, 183.041; ESI^-^: 283.3 [M-H]^-^, 239.2, 257.2; EI^+^: 284, 63, 126; ESI^+^(IT): 241, 267, 285.1 | 229, 258 (log E = 4.1, 3.2) | 10168/ [9](#_ENREF_9) |
| 41 | 6-Methyl-rhein | 401621-27-0 | R_1_=R_8_=OH R_2_=R_4_=R_5_=R_7_=H R_3_=COOH R_6_=CH_3_ | 298.0477 (C_16_H_10_O_6_) | ESI^+^: 298 [M]^+^, 281, 284, 242, 270, 107, 135 | 232, 248, 281, 448, 472 (log E = 4.42, 4.08, 4.18, 4.06, 4.08) | 3009664/ [19](#_ENREF_19) |
| 42 | Rheinal | 154658-30-7 | R_1_=R_8_=OH R_2_=R_4_=R_5_=R_6_=R_7_=H R_3_=CHO | 268.0372 (C_15_H_8_O_5_) | ESI^+^: 268 [M]^+^, 254, 212, 240, 93, 234, 119 | 230, 250, 279, 446, 470 (log E = 4.43, 4.11, 4.18, 4.06, 4.08) | 18996986/ [20](#_ENREF_20) |
| 43 | Diacerein | 13739-02-1 | R_1_=R_8_=OCOCH_3_ R_2_=R_4_=R_5_=R_6_=R_7_=H R_3_=COOH | 368.0532 (C_19_H_12_O_8_) |  |  | 26248/ [1](#_ENREF_1) |
| 44 | Rhein 8-*O*-*β*-D-glucoside | 34298-86-7 | R_1_=OH R_2_=R_4_=R_5_=R_6_=R_7_=H R_3_=COOH R_8_=O-Glc | 446.0849 (C_21_H_18_O_11_) | ESI^+^: 285; ESI^-^: 283 | 254 | 5320961/ |
| 45 | Rhein-8-*O*-*β*-D-[6' -*O*-(3''-methoxylmalonyl)]glucopyranoside | 1333328-11-2/ 2185837-12-9 | R_1_=OH R_2_=R_4_=R_5_=R_6_=R_7_=H R_3_=COOH R_8_=O-Glc^6^→O-COCH_2_COOCH_3_ | 546.1010 (C_25_H_22_O_14_) | ESI^+^: 1110.232 [2M+NH_4_]^+^; ESI^-^: 545 [M-H]-/ 1091 [2M-H]^-^ | 230, 260, 410 | [21](#_ENREF_21) |
| 46 | Rhein 11-*O*-*β*-D-glucoside | 67565-95-1 | R_1_=R_8_=OH R_2_=R_4_=R_5_=R_6_=R_7_=H R_3_=COO-Glc | 446.0849 (C_21_H_18_O_11_) | ESI^+^: 447 [M+H]^+^ | 227, 254, 430 (log E = 4.56, 4.3, 4.05) | [20](#_ENREF_20) |
| 47 | Rhein diglucoside | 106599-03-5/ 899815-64-6 | R_1_=R_8_=O-Glc R_2_=R_4_=R_5_=R_6_=R_7_=H R_3_=COOH | 608.1377 (C_27_H_28_O_16_) |  |  | 5320960/ TCMSP |
| 48 | Rhein-11-*O*-*β*-D-glucoside hexaacetate | 72380-12-2 | R_1_=R_8_=OCOCH_3_ R_2_=R_4_=R_5_=R_6_=R_7_=H  R_3_=COO | 698.1483 (C_33_H_30_O_17_) |  |  | [1](#_ENREF_1) |
| 49 | Laccaic acid D | 18499-84-8 | R_1_=CH_3_ R_2_=COOH R_3_=R_6_=R_8_=OH R_4_=R_5_=R_7_=H | 314.0426 (C_16_H_10_O_7_) |  |  | 9883304 |
| 50 | 1-Methyl-8-hydroxyl-9,10-anthraquinone-3-*O*-*β*-D-(6' -*O*-cinnamoyl)glucopyranoside |  | R_1_=CH_3_ R_2_=COOH R_3_=O-Glc^6^→O-Cin R_4_=R_5_=R_6_=R_7_=H R_8_=OH | 590.1424 (C_31_H_26_O_12_) | ESI^+^: 608.176 [M+NH_4_]^+^; ESI^-^: 589 [M-H]^-^ | 218, 269, 411 | [21](#_ENREF_21) |
| 1. Anthranones | | | | | | | |
| 1 | Emodin anthrone | 491-60-1 | R_1_=R_4_=R_5_=R_6_=H R_2_=CH_3_ R_3_=OH | 256.0736 (C_15_H_12_O_4_) |  |  | 122635/ TCMSP |
| 2 | Cassialoin | 60462-09-1 | R_1_=R_3_=R_4_=H R_2_=CH_3_ R_5_=OH R_6_=Glc | 418.1264 (C_21_H_22_O_9_) |  | 260, 268, 300, 365 (log E = 3.78, 3.91, 3.91, 4.08) | 101316715/ [22](#_ENREF_22) |
| 3 | Cascaroside C | 53823-09-9 | R_1_=R_3_=H R_2_=CH_3_ R_4_=R_6_=Glc R_5_=H (10*S*) | 564.1843 (C_27_H_32_O_13_) |  |  | 46173832 |
| 4 | 10-Hydroxycascaroside C |  | R_1_=R_3_=H R_2_=CH_3_ R_4_=R_6_=Glc R_5_=OH (10*R*) | 580.1792 (C_27_H_32_O_14_) | ESI^-^: 579.173 [M-H]^-^ | 266, 301, 322 (log E = 3.75, 3.89, 3.83) | 21577053/ [5](#_ENREF_5) |
| 5 | Cascaroside D | 53861-35-1 | R_1_=R_3_=H R_2_=CH_3_ R_4_=R_6_=Glc R_5_=H (10*R*) | 564.1843 (C_27_H_32_O_13_) |  |  | 46173831 |
| 6 | 10-Hydroxycascaroside D |  | R_1_=R_3_=H R_2_=CH_3_ R_4_=R_6_=Glc R_5_=OH (10*S*) | 580.1792 (C_27_H_32_O_14_) | ESI^-^: 579.172 [M-H]^-^ | 263, 298, 325 (log E = 3.73, 3.91, 3.86) | 21577054/ [5](#_ENREF_5) |
| 7 | 10*R*-Chrysaloin 1-*O*-*β*-D-glucoside |  | R_1_=R_6_=Glc R_2_=CH_3_ R_3_=R_4_=R_5_=H | 564.1843 (C_27_H_32_O_13_) | ESI^-^: 563.174 [M-H]^-^ | 249, 294, 321 (log E = 3.78, 3.91, 3.86) | [5](#_ENREF_5) |
| 8 | 4,5,9-Trihydroxy-10-oxo-9-[(2*R*,3*R*,4*S*,5*S*,6*R*)-3,4,5-trihydroxy-6-(hydroxymethyl)oxan-2-yl]anthracene-2-carboxylic acid |  | R_1_=R_3_=R_4_=H R_2_=COOH R_5_=OH R_6_=Glc | 448.1006 (C_21_H_20_O_11_) | ESI^+^: 448 [M]^+^ |  | 13888127/ [23](#_ENREF_23) |
| 9 | Rheinoside A | 111545-28-9 | R_1_=R_3_=H R_2_=COOH R_4_=R_6_=Glc R_5_=OH (10*S*) | 610.1534 (C_27_H_30_O_16_) | ESI^+^: 633 [M+Na]^+^ | 267, 296, 346 | 13888123/ [23](#_ENREF_23) |
| 10 | Rheinoside B | 111614-10-9 | R_1_=R_3_=H R_2_=COOH R_4_=R_6_=Glc R_5_=OH (10*R*) | 610.1534 (C_27_H_30_O_16_) | ESI^+^: 633 [M+Na]^+^ | 269, 298, 345 | [23](#_ENREF_23) |
| 11 | Rheinoside C | 111545-29-0 | R_1_=R_3_=H R_2_=COOH R_4_=R_6_=Glc R_5_=H (10*R*) | 594.1585 (C_27_H_30_O_15_) | ESI^+^: 617 [M+Na]^+^ | 268, 292, 335 | 13888128/ [23](#_ENREF_23) |
| 12 | Rheinoside D | 111614-11-0 | R_1_=R_3_=H R_2_=COOH R_4_=R_6_=Glc R_5_=H (10*S*) | 594.1585 (C_27_H_30_O_15_) | ESI^+^: 617 [M+Na]^+^ | 270, 295, 327 | [23](#_ENREF_23) |
| 13 | Revandchinone 1 |  | R_1_=R_4_=R_5_=H R_2_=CH_3_ R_3_=OCH_3_ R_6_=OCO(CH_2_)_7_CHCH(CH_2_)_7_CH_3_ | 550.3294 (C_34_H_46_O_6_) | ESI^+^: 551 [M+H]^+^; EI^+^: 57, 85, 149, 284, 232 | 440, 285 | 5320939/ [10](#_ENREF_10) |
| 14 | Revandchinone 2 |  | R_1_=R_3_=R_4_=R_5_=H R_2_=CH_3_ R_6_=OCO(CH_2_)_26_CH_3_ | 662.4910 (C_43_H_66_O_5_) | ESI^+^: 663 [M+H]^+^; EI^+^: 104, 254, 204 | 440, 290 | 5320940/ [10](#_ENREF_10) |
| 15 | Revandchinone 4 |  | R_1_=R_4_=H R_2_=CH_2_OH R_3_=R_5_=OH R_6_=O(CH_2_)_17_CH_3_ | 556.3400 (C_33_H_48_O_7_) | ESI^+^: 579 [M+Na]^+^ | 440, 285 | 5320942/ [10](#_ENREF_10) |
|  | | | | | | | |
| 16 | Emodin bianthrone | 2506-11-8 | R_1_=R_4_=CH_3_ R_2_=R_5_=OH R_3_=R_6_=H | 510.1315 (C_30_H_22_O_8_) |  | 220, 262, 276, 365 | 10097848/ [24](#_ENREF_24) |
| 17 | Palmidin A | 17062-55-4 | R_1_=CH_2_OH R_2_=R_3_=R_6_=H R_4_=CH_3_ R_5_=OH | 510.1315 (C_30_H_22_O_8_) |  | 220, 258, 278, 365 | 5320384/ [24](#_ENREF_24) |
| 18 | Palmidin B | 17062-56-5 | R_1_=CH_3_ R_2_=R_3_=R_5_=R_6_=H R_4_=CH_2_OH | 494.1365 (C_30_H_22_O_7_) |  | 220, 258, 271 | 5320385/ [24](#_ENREF_24) |
| 19 | Palmidin C | 17177-86-5 | R_1_=R_4_=CH_3_ R_2_=R_3_=R_6_=H R_5_=OH | 494.1365 (C_30_H_22_O_7_) |  | 220, 258, 276, 360 | 5320386/ 44567180/ [24](#_ENREF_24) |
| 20 | Rheidin A |  | R_1_=COOH R_2_=R_3_=R_6_=H R_4_=CH_3_ R_5_=OH | 524.1107 (C_30_H_20_O_9_) |  |  | 5320957 |
| 21 | Rheidin B |  | R_1_=COOH R_2_=R_3_=R_5_=R_6_=H R_4_=CH_3_ | 508.1158 (C_30_H_20_O_8_) |  |  | 5320958 |
| 22 | Rheidin C |  | R_1_=COOH R_2_=R_3_=R_6_=H R_4_=CH_3_ R_5_=OCH_3_ | 538.1264 (C_31_H_22_O_9_) |  |  | 5320959 |
| 23 | Sennidin A | 641-12-3 | R_1_=R_4_=COOH R_2_=R_3_=R_5_=R_6_=H (10,10'-*trans*) | 538.0900  (C_30_H_18_O_10_) |  |  | 92826/ [25](#_ENREF_25) |
| 24 | Sennoside A | 81-27-6/ 8013-11-4 | R_1_=R_4_=COOH R_2_=R_5_=H R_3_=R_6_=Glc (10,10'-*trans*) | 862.1956 (C_42_H_38_O_20_) | ESI^+^: 880.23 [M+NH_4_]^+^, 270.052, 271.057, 269.045; ESI^-^: 861.189 [M-H]^-^, 227.032, 386.097, 224.046 | 340 | 73111/ [13](#_ENREF_13) |
| 25 | Sennidin B | 517-44-2 | R_1_=R_4_=COOH R_2_=R_3_=R_5_=R_6_=H (10,10'-*cis*) | 538.0900  (C_30_H_18_O_10_) | ESI^-^(IT): 537.083 [M-H]^-^, 493, 224, 268 |  | 10459879 |
| 26 | Sennoside B | 128-57-4 | R_1_=R_4_=COOH R_2_=R_5_=H R_3_=R_6_=Glc (10,10'-*cis*) | 862.1956 (C_42_H_38_O_20_) | ESI^-^: 861.186 [M-H]^-^, 430.088, 430.588 | 270, 308, 355 | 91440/ [26](#_ENREF_26) |
| 27 | Sennidin C | 5355-93-1 | R_1_=COOH R_2_=R_3_=R_5_=R_6_=H R_4_=CH_2_OH (10,10'-*trans*) | 524.1107 (C_30_H_20_O_9_) |  |  | 5321255 |
| 28 | Sennoside C | 37271-16-2 | R_1_=COOH R_2_=R_5_=H R_3_=R_6_=Glc R_4_=CH_2_OH (10,10'-*trans*) | 848.2164 (C_42_H_40_O_19_) | ESI^+^: 255, 271; ESI^-^: 253, 431 | 270, 323 | 46173829/ [26](#_ENREF_26) |
| 29 | Sennidin D | 62279-78-1 | R_1_=COOH R_2_=R_3_=R_5_=R_6_=H R_4_=CH_2_OH (10,10'-*cis*) | 524.1107 (C_30_H_20_O_9_) |  |  | TCMSP |
| 30 | Sennoside D | 37271-17-3 | R_1_=COOH R_2_=R_5_=H R_3_=R_6_=Glc R_4_=CH_2_OH (10,10'-*cis*) | 848.2164 (C_42_H_40_O_19_) |  |  | 46173830 |
| 31 | Sennoside E | 792907-49-4 | R_1_=R_4_=COOH R_2_=R_5_=H R_3_=Glc R_6_=Glc^6^→O-COCOOH (10,10'-*trans*) | 934.1804 (C_44_H_38_O_23_) |  |  | 73425506 |
| 32 | Sennoside F | 1037532-47-0 | R_1_=R_4_=COOH R_2_=R_5_=H R_3_=Glc R_6_=Glc^6^→O-COCOOH (10,10'-*cis*) | 934.1804 (C_44_H_38_O_23_) |  |  | 72941975 |
| 1. Tannins and acylglycosides | | | | | | | |
| 1 | Cianidanol | 154-23-4/ 100786-01-4 | R_1_=R_2_=R_3_=R_4_=R_5_=R_6_=R_7_=R_8_=H (3*S*) | 290.0790 (C_15_H_14_O_6_) | ESI^+^: 291.08 [M+H]+/ 313.06 [M+Na]^+^, 130.05, 130.049, 162.035/ 139.034, 123.039, 165.049/ 271.121, 272.126, 253.134; ESI^-^: 289.064 [M-H]^-^, 160.018, 189.014, 245.005 | 280 (log E = 3.59) | 9064/ [27](#_ENREF_27) |
| 2 | (+)-Catechin 5-*O*-*β*-D-glucopyranoside | 88126-53-8 | R_1_=R_3_=R_4_=R_5_=R_6_=R_7_=R_8_=H R_2_=Glc (3*S*) | 452.1319 (C_21_H_24_O_11_) |  |  | 6324898 |
| 3 | (+)-Catechin 6-*C*-glucoside | 105371-27-5 | R_1_=R_2_=R_4_=R_5_=R_6_=R_7_=R_8_=H R_3_=Glc (3*S*) | 452.1319 (C_21_H_24_O_11_) | ESI^+^: 452 [M]^+^, 434, 290 |  | 21626712/ [28](#_ENREF_28) |
| 4 | (+)-Catechin 7-*O*-*β*-D-glucopyranoside | 88197-03-9 | R_1_=R_2_=R_3_=R_5_=R_6_=R_7_=R_8_=H R_4_=Glc (3*S*) | 452.1319 (C_21_H_24_O_11_) |  |  | 44257085 |
| 5 | (+)-Catechin 8-*C*-glucoside | 105371-26-4 | R_1_=R_2_=R_3_=R_4_=R_6_=R_7_=R_8_=H R_5_=Glc (3*S*) | 452.1319 (C_21_H_24_O_11_) | ESI^+^: 453 [M+H]^+^, 434 |  | 21626713/ [28](#_ENREF_28) |
| 6 | (+)-Catechin 3'-*O*-*β*-D-glucopyranoside | 150337-42-1 | R_1_=R_2_=R_3_=R_4_=R_5_=R_7_=R_8_=H R_6_=Glc (3*S*) | 452.1319 (C_21_H_24_O_11_) | ESI^+^: 491 [M+K]+/ 475 [M+Na]+/ 453 [M+H]^+^, 290, 163 |  | 44257088/ [28](#_ENREF_28) |
| 7 | (+)-Catechin 4'-*O*-*β*-D-glucopyranoside | 105330-52-7/ 1037072-19-7/ 183387-70-4/ 1705566-89-7 | R_1_=R_2_=R_3_=R_4_=R_5_=R_6_=R_8_=H R_7_=Glc (3*S*) | 452.1319 (C_21_H_24_O_11_) | ESI^+^: 491 [M+K]+/ 475 [M+Na]+/ 453 [M+H]^+^, 290, 163 |  | 44257089/ [28](#_ENREF_28) |
| 8 | (+)-Catechin 5,3'-di-*O*-*β*-D-glucopyranoside | 105330-54-9 | R_1_=R_3_=R_4_=R_5_=R_7_=R_8_=H R_2_=R_6_=Glc (3*S*) | 614.1847 (C_27_H_34_O_16_) | ESI^+^: 637 [M+Na]^+^, 452, 290, 163 |  | 44257090/ [28](#_ENREF_28) |
| 9 | (+)-Catechin 5,4'-di-*O*-*β*-D-glucopyranoside | 105330-56-1 | R_1_=R_3_=R_4_=R_5_=R_6_=R_8_=H R_2_=R_7_=Glc (3*S*) | 614.1847 (C_27_H_34_O_16_) | ESI^+^: 637 [M+Na]+/ 615 [M+H]^+^, 475, 453, 290, 163 |  | 44257091/ [28](#_ENREF_28) |
| 10 | (+)-Catechin 7,3'-di-*O*-*β*-D-glucopyranoside | 105330-53-8 | R_1_=R_2_=R_3_=R_5_=R_7_=R_8_=H R_4_=R_6_=Glc (3*S*) | 614.1847 (C_27_H_34_O_16_) | ESI^+^: 637 [M+Na]+/ 615 [M+H]^+^, 452, 290 |  | 44257092/ [28](#_ENREF_28) |
| 11 | (+)-Catechin 3',4'-di-*O*-*β*-D-glucopyranoside | 105330-55-0 | R_1_=R_2_=R_3_=R_4_=R_5_=R_8_=H R_6_=R_7_=Glc (3*S*) | 614.1847 (C_27_H_34_O_16_) | ESI^+^: 637 [M+Na]^+^, 452, 290, 163 |  | 44257093/ [28](#_ENREF_28) |
| 12 | Catechol pentaacetate | 16198-01-9 | R_1_=R_2_=R_4_=R_6_=R_7_=COCH_3_ R_3_=R_5_=R_8_=H (3*S*) | 500.1319 (C_25_H_24_O_11_) | ESI^+^: 518.16 [M+NH_4_]^+^, 399.1, 357.09/ 315.084 |  | 5315742/ TCMSP |
| 13 | (-)-Epicatechin | 490-46-0/ 7295-85-4 | R_1_=R_2_=R_3_=R_4_=R_5_=R_6_=R_7_=R_8_=H (3*R*) | 290.0790 (C_15_H_14_O_6_) | ESI^+^: 291.085 [M+H]^+^,139.039 , 123.046, 147.041/ 207.06; ESI^-^: 289.072/ 289.064 [M-H]^-^, 245.08, 109.028/ 125.013; EI^+^: 368, 179, 355; ESI^+^(IT): 123, 139, 165 | 280 | 72276/ [1](#_ENREF_1) |
| 14 | (-)-Epicatechin gallate | 1257-08-5 | R_1_=G R_2_=R_3_=R_4_=R_5_=R_6_=R_7_=R_8_=H (3*R*) | 442.0900 (C_22_H_18_O_10_) | ESI^-^: 441.08 [M-H]^-^, 169.013/ 125.023, 289.07; ESI^+^(IT): 443.097 [M+H]^+^, 273, 151, 291.1 | 276 | 107905/ [29](#_ENREF_29) |
| 15 | Epicatechin pentaacetate | 20194-41-6 | R_1_=R_2_=R_4_=R_6_=R_7_=COCH_3_ R_3_=R_5_=R_8_=H (3*R*) | 500.1319 (C_25_H_24_O_11_) | ESI^+^: 518.163 [M+NH_4_]^+^, 399.106, 357.096/ 315.086 |  | 5317058/ TCMSP |
| 16 | (+)-Gallocatechin | 970-73-0/ 1617-55-6 | R_1_=R_2_=R_3_=R_4_=R_5_=R_6_=R_7_=H R_8_=OH (3*S*) | 306.0739 (C_15_H_14_O_7_) | ESI^-^: 305.1 [M-H]^-^, 125.025, 137.025, 109.03/ 139.04/ 179.032, 221.043, 219.063 | 271 | 65084/ [30](#_ENREF_30) |
|  | | | | | | | |
| 17 | Procyanidin B1 | 20315-25-7 | R_1_=R_7_=OH R_2_=R_3_=R_4_=R_5_=R_6_=H | 578.1424 (C_30_H_26_O_12_) | ESI^+^: 579.15 [M+H]^+^, 127.04, 139.04; ESI^-^: 577.135 [M-H]^-^, 125.02, 289.07/ 245.081, 407.078; ESI^+^(IT): 427, 409, 291 |  | 11250133 |
| 18 | Procyanidin B1 3-*O*-gallate | 79907-45-2 | R_1_=O-G R_2_=R_3_=R_4_=R_5_=R_6_=H R_7_=OH | 730.1534 (C_37_H_30_O_16_) |  |  | 12795888 |
| 19 | Procyanidin B1 6-*C*-*β*-D-glucopyranoside | 105371-29-7 | R_1_=R_7_=OH R_2_=R_4_=R_5_=R_6_=H R_3_=Glc | 740.1952 (C_36_H_36_O_17_) | ESI^+^: 741 [M+H]^+^ |  | 21636073/ [28](#_ENREF_28) |
| 20 | Procyanidin B1 8-*C*-*β*-D-glucopyranoside | 105371-28-6 | R_1_=R_7_=OH R_2_=R_3_=R_4_=R_6_=H R_5_=Glc | 740.1952 (C_36_H_36_O_17_) | ESI^+^: 741 [M+H]^+^ |  | 21636072/ [28](#_ENREF_28) |
| 21 | Procyanidin B2 | 29106-49-8 | R_1_=R_6_=OH R_2_=R_3_=R_4_=R_5_=R_7_=H | 578.1424 (C_30_H_26_O_12_) | ESI^+^: 579.1 [M+H]+/ 601.089 [M+Na]^+^, 127.039, 139.039/ 289.048, 291.063, 427.07/ 311.029, 449.054; ESI^-^: 577.135 [M-H]^-^, 125.025, 289.073 | 280 | 122738/ [31](#_ENREF_31) |
| 22 | Procyanidin B2 3'-*O*-gallate | 73086-04-1 | R_1_=OH R_2_=R_3_=R_4_=R_5_=R_7_=H R_6_=O-G | 730.1534 (C_37_H_30_O_16_) |  |  | 15593124 |
| 23 | Procyanidin B2 3,3'-di-*O*-gallate | 79907-44-1 | R_1_=R_6_=O-G R_2_=R_3_=R_4_=R_5_=R_7_=H | 882.1643 (C_44_H_34_O_20_) |  |  | 124016 |
| 24 | Procyanidin B2 6-*C*-*β*-D-glucopyranoside | 103215-59-4 | R_1_=R_6_=OH R_2_=R_4_=R_5_=R_7_=H R_3_=Glc | 740.1952 (C_36_H_36_O_17_) |  |  | 131752185 |
| 25 | Procyanidin B2 8-*C*-*β*-D-glucopyranoside | 103215-58-3 | R_1_=R_6_=OH R_2_=R_3_=R_4_=R_7_=H R_5_=Glc | 740.1952 (C_36_H_36_O_17_) |  |  | 21637579 |
| 26 | Procyanidin B3 | 23567-23-9 | R_1_=R_3_=R_4_=R_5_=R_6_=H R_2_=R_7_=OH | 578.1424 (C_30_H_26_O_12_) | ESI^+^: 579 [M+H]^+^, 427, 409, 291 |  | 146798 |
| 27 | Procyanidin B3 7-*O*-*β*-D-glucopyranoside | 105330-57-2 | R_1_=R_3_=R_5_=R_6_=H R_2_=R_7_=OH R_4_=Glc | 740.1952 (C_36_H_36_O_17_) | ESI^+^: 741 [M+H]^+^ |  | 21636071/ [28](#_ENREF_28) |
| 28 | Procyanidin B4 | 29106-51-2 | R_1_=R_3_=R_4_=R_5_=R_7_=H R_2_=R_6_=OH | 578.1424 (C_30_H_26_O_12_) | ESI^+^: 579 [M+H]^+^, 427, 409, 291 |  | 147299 |
| 29 | Procyanidin B4 3'-*O*-gallate |  | R_1_=R_3_=R_4_=R_5_=R_7_=H R_2_=OH R_6_=O-G | 730.1534 (C_37_H_30_O_16_) |  |  | 15593123 |
|  | | | | | | | |
| 30 | Procyanidin B5 | 12798-57-1 | R_1_=R_3_=H R_2_=OH | 578.1424 (C_30_H_26_O_12_) |  |  | 124017 |
| 31 | Procyanidin B5 3'-*O*-gallate | 249287-43-2 | R_1_=R_3_=H R_2_=O-G | 730.1534 (C_37_H_30_O_16_) |  |  | 102005056/ TCMSP |
| 32 | Procyanidin B5 3,3'-di-*O*-gallate | 106533-60-2 | R_1_=G R_2_=O-G R_3_=H | 882.1643 (C_44_H_34_O_20_) |  |  | 3086518 |
| 33 | Procyanidin B7 | 12798-59-3 | R_1_=R_2_=H R_3_=OH | 578.1424 (C_30_H_26_O_12_) |  |  | 13990893 |
| 34 | Procyanidin B7 3-*O*-gallate | 106533-59-9 | R_1_=G R_2_=H R_3_=OH | 730.1534 (C_37_H_30_O_16_) |  |  | 21626728 |
|  | | | | | | | |
| 35 | Procyanidin C1 | 37064-30-5 | R_1_=R_2_=R_4_=H R_3_=OH | 866.2058 (C_45_H_38_O_18_) | ESI^+^: 866 [M]^+^, 865, 864; ESI^-^: 865.23 [M-H]^-^, 866.242, 125.029 |  | 169853/ |
| 36 | Procyanidin C1 3',3''-di-*O*-gallate | 106533-61-3 | R_1_=R_4_=H R_2_=G R_3_=O-G | 1170.2277 (C_59_H_46_O_26_) |  |  | 21676353 |
| 37 | Procyanidin C1 3,3',3''-tri-*O*-gallate | 106533-62-4 | R_1_=R_2_=G R_3_=O-G R_4_=H | 1322.2387 (C_66_H_50_O_30_) |  |  | 16152264 |
| 38 | Procyanidin T2 | 79813-67-5 | R_1_=R_2_=R_3_=H R_4_=OH | 866.2058 (C_45_H_38_O_18_) | ESI^+^: 867 [M+H]^+^, 579, 715, 427 |  | 13752000 |
| 39 | Procyanidin T2 3,3'-di-*O*-gallate |  | R_1_=R_2_=G R_3_=H R_4_=OH | 1170.2277 (C_59_H_46_O_26_) |  |  | 16142157 |
|  | | | | | | | |
| 40 | Arecatannin B1 | 79763-28-3 | R_1_=H | 866.2058 (C_45_H_38_O_18_) |  |  | 14237657 |
| 41 | Arecatannin B1 3,3'-digallate | 106533-65-7 | R_1_=G | 1170.2277 (C_59_H_46_O_26_) |  |  | 131752219 |
| 42 | Epicatechin-(4*β*-6)-epicatechin-(4*β*-8)-epicatechin | 99297-47-9/ 101469-10-7 | R_2_=R_4_=H R_3_=OH | 866.2058 (C_45_H_38_O_18_) |  |  | 10865804 |
| 43 | 3-*O*-Galloylepicatechin-(4*β*-6)-3-*O*-galloylepicatechin-(4*β*-8)-3-*O*-galloylepicatechin | 106533-64-6 | R_2_=G R_3_=O-G R_4_=H | 1322.2387 (C_66_H_50_O_30_) |  |  | 21676352 |
| 44 | Epicatechin-(4*β*-6)-epicatechin-(4*β*-8)-catechin | 82801-35-2 | R_2_=R_3_=H R_4_=OH | 866.2058 (C_45_H_38_O_18_) |  |  | 14309769/ [32](#_ENREF_32) |
| 45 | 3-*O*-Galloylepicatechin-(4*β*-6)-3-*O*-galloylepicatechin-(4*β*-8)-catechin | 106548-99-6 | R_2_=G R_3_=H R_4_=OH | 1170.2277 (C_59_H_46_O_26_) |  |  | 21676355 |
| 46 | Epicatechin-(4*β*-6)-epicatechin-(4*β*-6)-epicatechin | 101401-64-3 | R_5_=H | 866.2058 (C_45_H_38_O_18_) |  |  | 12003800 |
| 47 | 3-*O*-Galloylepicatechin-(4*β*-6)-3-*O*-galloylepicatechin-(4*β*-6)-3-*O*-galloylepicatechin |  | R_5_=G | 1322.2387 (C_66_H_50_O_30_) |  |  | 21676354 |
|  | | | | | | | |
| 48 | Gallic acid | 149-91-7/ 6274-40-4 | R_1_=COOH R_2_=R_3_=R_4_=H | 170.0215 (C_7_H_6_O_5_) | ESI^+^: 171.028 [M+H]^+^, 153.018, 125.022, 127.038; ESI^-^: 169.01 [M-H]^-^, 125.023, 97.028/ 79.019, 124.017; EI^+^: 281, 179, 282; ESI^-^(IT): 125.1, 169.1, 125.8 | 272.5 (log E = 4.06) | 370/ [31](#_ENREF_31) |
| 49 | 4-*O*-Methylgallic acid | 4319-02-2 | R_1_=COOH R_2_=R_4_=H R_3_=CH_3_ | 184.0372 (C_8_H_8_O_5_) | EI^+^: 184, 169, 113; ESI^+^(IT): 185.044 [M+H]^+^, 141, 167, 126; ESI^-^(IT): 183.03 [M-H]^-^, 168, 183.9, 183 |  | 78016/ [1](#_ENREF_1) |
| 50 | Gallic acid 3-*O*-*β*-D-glucopyranoside | 91984-84-8 | R_1_=COOH R_2_=Glc R_3_=R_4_=H | 332.0743 (C_13_H_16_O_10_) |  |  | 101683334 |
| 51 | Gallic acid 4-*O*-*β*-D-glucopyranoside | 84274-52-2 | R_1_=COOH R_2_=R_4_=H R_3_=Glc | 332.0743 (C_13_H_16_O_10_) |  |  | 10088114 |
| 52 | Gallic acid 3-*O*-(6-galloylglucoside) | 87087-61-4 | R_1_=COOH R_2_=Glc^6^→O-G R_3_=R_4_=H | 484.0853 (C_20_H_20_O_14_) |  |  | 73157749 |
| 53 | Gallic acid 4-*O*-(6-galloylglucoside) | 87087-62-5 | R_1_=COOH R_2_=R_4_=H R_3_=Glc^6^→O-G | 484.0853 (C_20_H_20_O_14_) |  |  | 73157750 |
| 54 | 1-*O*-Galloylglycerol | 87087-60-3 | R_1_=COOCH_2_CHOHCH_2_OH R_2_=R_3_=R_4_=H | 244.0583 (C_10_H_12_O_7_) | ESI^+^: 244 [M]^+^, 153 | 221, 276 (log E = 4.47, 4.1) | 5317465/ |
| 55 | 6-*O*-Galloylglucose | 13186-19-1 | R_1_=COOCH_2_(CHOH)_4_CHO R_2_=R_3_=R_4_=H | 332.0743 (C_13_H_16_O_10_) |  |  | 128839/ [1](#_ENREF_1) |
| 56 | *β*-Glucogallin | 13405-60-2/ 554-37-0 | R_1_=COO-Glc R_2_=R_3_=R_4_=H | 332.0743 (C_13_H_16_O_10_) | ESI^-^: 332.074 [M]^-^, 211.022, 169.013, 151.003 |  | 124021 |
| 57 | 1-*O*-Galloyl-2-*O*-cinnamoylglucose | 56994-83-3 | R_1_=COO-Glc^2^→O-Cin R_2_=R_3_=R_4_=H | 462.1162 (C_22_H_22_O_11_) |  |  | 5315898 |
| 58 | 1-*O*-Galloyl-6-*O*-cinnamoylglucose | 115746-69-5 | R_1_=COO-Glc^6^→O-Cin R_2_=R_3_=R_4_=H | 462.1162 (C_22_H_22_O_11_) | ESI^+^: 485.11 [M+Na]+/ 480.15 [M+NH_4_]^+^, 315.086, 486.108/ 131.049, 103.054, 149.06 |  | 14034234 |
| 59 | 1-*O*-Galloyl-2-*O*-*p*-coumaroylglucose | 94356-18-0 | R_1_=COO-Glc^2^→O-*p*-Coum R_2_=R_3_=R_4_=H | 478.1111 (C_22_H_22_O_12_) | ESI^+^: 501 [M+Na]^+^, 308, 170 |  | 131752570/ [35](#_ENREF_35) |
| 60 | 1-*O*-Galloyl-6-*O*-*p*-coumaroylglucose | 1175782-92-9/ 1176521-18-8 | R_1_=COO-Glc^6^→O-*p*-Coum R_2_=R_3_=R_4_=H | 478.1111 (C_22_H_22_O_12_) | ESI^+^: 496.145 [M+NH_4_]^+^, 147.044, 119.049, 148.047; ESI^-^: 477.1 [M-H]^-^, 169.015, 265.074 |  | 45359700/ [25](#_ENREF_25) |
| 61 | 1,2-Di-*O*-galloylglucose | 115713-50-3 | R_1_=COO-Glc^2^→O-G R_2_=R_3_=R_4_=H | 484.0853 (C_20_H_20_O_14_) | ESI^+^: 484 [M]^+^, 314, 170 |  | 14034255/ [36](#_ENREF_36) |
| 62 | 1,6-Di-*O*-galloylglucose | 23363-08-8 | R_1_=COO-Glc^6^→O-G R_2_=R_3_=R_4_=H | 484.0853 (C_20_H_20_O_14_) | ESI^+^: 502.119 [M+NH_4_]^+^, 153.019, 297.062, 315.072; ESI^-^: 483.08 [M-H]^-^, 271.045, 169.012 |  | 3332212 |
| 63 | 1,2-Di-*O*-galloyl-6-*O*-cinnamoylglucose | 115746-70-8 | R_1_=COO-Glc^2^→O-G  ↓^6^  O-Cin R_2_=R_3_=R_4_=H | 614.1272 (C_29_H_26_O_15_) | ESI^+^: 615 [M+H]^+^, 170, 148 |  | 14034236/ [36](#_ENREF_36) |
| 64 | 1,6-Di-*O*-galloyl-2-*O*-cinnamoylglucose | 94356-17-9 | R_1_=COO-Glc^2^→O-Cin  ↓^6^  O-G R_2_=R_3_=R_4_=H | 614.1272 (CH_26_O_15_) | ESI^+^: 653 [M+K]+/ 637 [M+Na]^+^, 170, 148 |  | 6325082/ [35](#_ENREF_35) |
| 65 | 1,2-Di-*O*-galloyl-6-*O*-*p*-coumaroylglucose |  | R_1_=COO-Glc^2^→O-G  ↓^6^  O-*p*-Coum R_2_=R_3_=R_4_=H | 630.1221 (C_29_H_26_O_16_) | ESI^+^: 631 [M+H]^+^, 170, 164; ESI^-^: 629.115 [M-H]^-^, 169.013, 465.068, 313.057 |  | 14034239/ [36](#_ENREF_36) |
| 66 | 1,6-Di-*O*-galloyl-2-*O*-*p*-coumaroylglucose | 115713-49-0 | R_1_=COO-Glc^2^→O-*p*-Coum  ↓^6^  O-G R_2_=R_3_=R_4_=H | 630.1221 (C_29_H_26_O_16_) | ESI^+^: 631 [M+H]^+^, 460, 170, 164 |  | 14034242/ [36](#_ENREF_36) |
| 67 | 1,2,6-Tri-*O*-galloylglucose | 79886-49-0 | R_1_=COO-Glc^2,6^→O-G R_2_=R_3_=R_4_=H | 636.0963 (C_27_H_24_O_18_) | ESI^-^: 635.089 [M-H]-/ 671.065 [M+Cl]^-^, 465.06, 169.014, 313.06/ 635.08 |  | 440308 |
| 68 | 1,3,6-Tri-*O*-galloylglucose | 18483-17-5 | R_1_=COO-Glc^3,6^→O-G R_2_=R_3_=R_4_=H | 636.0963 (C_27_H_24_O_18_) | ESI^+^: 654.13 [M+NH_4_]^+^, 153.018, 171.03, 467.079; ESI^-^: 635.09 [M-H]^-^, 169.013, 271.047 |  | 452707/ 11969003/ TCMSP |
| 69 | *β*-Penta-*O*-galloyl-D-glucose | 14937-32-7 | R_1_=COO-Glc^2,3,4,6^→O-G R_2_=R_3_=R_4_=H | 940.1182 (C_41_H_32_O_26_) |  |  | 374874/ [25](#_ENREF_25) |
| 70 | 1-*O*-Galloylfructose | 94356-19-1 | R_1_= R_2_=R_3_=R_4_=H | 332.0743 (C_13_H_16_O_10_) | ESI^+^: 355 [M+Na]^+^ |  | 131752599/ [35](#_ENREF_35) |
| 71 | 1-*O*-(3,4,5-Trihydroxybenzoyl)-*β*-D-fructofuranose |  | R_1_= R_2_=R_3_=R_4_=H | 332.0743 (C_13_H_16_O_10_) | ESI^+^: 355 [M+Na]^+^ |  | 102517396/ [35](#_ENREF_35) |
| 72 | Sinapyl alcohol diacetate | 211366-75-5 | R_1_=CHCHCH_2_OCOCH_3_ R_2_=R_4_=CH_3_ R_3_=COCH_3_ | 294.1103 (C_15_H_18_O_6_) |  |  | 21627708/ [1](#_ENREF_1) |
|  | | | | | | | |
| 73 | 2-Cinnamoylglucose | 94356-16-8 | R_1_=R_3_=R_4_=H R_2_=Cin | 310.1052 (C_15_H_18_O_7_) | ESI^+^: 310 [M]^+^, 148 |  | 14345566/ 11972309/ [35](#_ENREF_35) |
| 74 | 6-Cinnamoylglucose | 64461-97-8 | R_1_=R_2_=R_3_=H R_4_=Cin | 310.1052 (C_15_H_18_O_7_) |  |  | 21668944/ [25](#_ENREF_25) |
| 75 | 2-*p*-Coumaroylglucose | 110351-44-5 | R_1_=R_3_=R_4_=H R_2_=*p*-Coum | 326.1002 (C_15_H_18_O_8_) |  |  | 14034264/ [25](#_ENREF_25) |
| 76 | 6-*p*-Coumaroylglucose | 114297-65-3 | R_1_=R_2_=R_3_=H R_4_=*p*-Coum | 326.1002 (C_15_H_18_O_8_) | ESI^+^: 326 [M]^+^ |  | 13915664/ [36](#_ENREF_36) |
| 77 | 2-Galloylglucose | 98917-85-2 | R_1_=R_3_=R_4_=H R_2_=G | 332.0743 (C_13_H_16_O_10_) |  |  | 14034252 |
| 78 | 6-Galloylglucose | 34781-46-9 | R_1_=R_2_=R_3_=H R_4_=G | 332.0743 (C_13_H_16_O_10_) | ESI^+^: 332 [M]^+^ |  | 250398/ [33](#_ENREF_33) |
| 79 | 2,3-Digalloylglucose | 469-32-9 | R_1_=R_4_=H R_2_=R_3_=G | 484.0853 (C_20_H_20_O_14_) |  |  | 471118/ TCMSP |
| 80 | 2,6-Digalloylglucose | 94356-20-4 | R_1_=R_3_=H R_2_=R_4_=G | 484.0853 (C_20_H_20_O_14_) | ESI^+^: 467, 170 |  | 14034261/ [35](#_ENREF_35) |
| 81 | 3,5-Dihydroxyphenyl 1-*O*-(6-*O*-galloyl-*β*-D-glucopyranoside) | 94356-21-5 | R_1_= R_2_=R_3_=H R_4_=G | 440.0955 (C_19_H_20_O_12_) | ESI^+^: 441 [M+H]^+^, 315, 170, 126 |  | 131752603/ [35](#_ENREF_35) |
|  | | | | | | | |
| 82 | 2-*O*-Galloylsucrose | 115713-46-7 | R_1_=G R_2_=R_3_=R_4_=R_5_=H | 494.1272 (C_19_H_26_O_15_) | ESI^+^: 517 [M+Na]+/ 495 [M+H]^+^, 153 |  | 14055563/ [37](#_ENREF_37) |
| 83 | 6-*O*-Galloylsucrose | 115731-15-2 | R_1_=R_3_=R_4_=R_5_=H R_2_=G | 494.1272 (C_19_H_26_O_15_) | ESI^+^: 517 [M+Na]+/ 495 [M+H]^+^, 153 |  | 14055557/ [37](#_ENREF_37) |
| 84 | 1'-*O*-Galloylsucrose | 115713-45-6 | R_1_=R_2_=R_4_=R_5_=H R_3_=G | 494.1272 (C_19_H_26_O_15_) | ESI^+^: 517 [M+Na]+/ 495 [M+H]^+^, 153 |  | 14055559/ [37](#_ENREF_37) |
| 85 | 4'-*O*-Galloylsucrose | 115713-44-5 | R_1_=R_2_=R_3_=R_5_=H R_4_=G | 494.1272 (C_19_H_26_O_15_) | ESI^+^: 517 [M+Na]+/ 495 [M+H]^+^, 153 |  | 14055553/ [37](#_ENREF_37) |
| 86 | 6'-*O*-Galloylsucrose | 115713-43-4 | R_1_=R_2_=R_3_=R_4_=H R_5_=G | 494.1272 (C_19_H_26_O_15_) | ESI^+^: 517 [M+Na]+/ 495 [M+H]^+^, 153 |  | 14055547/ [37](#_ENREF_37) |
|  | | | | | | | |
| 87 | Cyanidin | 13306-05-3 |  | 287.0556 (C_15_H_11_O_6_^+^) | ESI^+^: 287 [M]^+^, 288.061, 289.053/ 137.022, 213.055/ 286 |  | 128861/ [25](#_ENREF_25) |
| 88 | Cyanidin 3-glucoside | 7084-24-4 |  | 484.0772 (C_21_H_21_ClO_11_) |  | 510 | 197081/ [38](#_ENREF_38) |
| 89 | Keracyanin | 18719-76-1 |  | 630.1351 (C_27_H_31_ClO_15_) |  |  | 29231/ [25](#_ENREF_25) |
| 90 | [Epicatechin-(4*β*-8)]5-epicatechin | 88847-05-6 |  | 1730.3960 (C_90_H_74_O_36_) |  |  | 16129623/ TCMSP |
| 91 | Rhatannin | 80456-02-6 |  |  |  |  | [39](#_ENREF_39) |
| 92 | RG-tannin |  |  |  |  |  | [39](#_ENREF_39) |
| 93 | 3,5-Di-*O*-galloyl-4-*O*-digalloylquinic acid | 123134-19-0 |  | 800.1072 (C_35_H_28_O_22_) |  |  | 442676/ TCMSP |
| 94 | 1-*O*-Galloylpedunculagin | 79786-00-8 |  | 936.0869 (C_41_H_28_O_26_) |  |  | 452242/ TCMSP |
| 95 | Tannic acid | 5424-20-4/ 1401-55-4 |  | 1700.1730 (C_76_H_52_O_46_) |  | 280, 420, 500 (log E = 2.2, 1.8, 1.8) | 16129778/ TCMSP |
| 1. Stilbenes | | | | | | | |
| 1 | 3,3',5-Trihydroxystilbene | 150258-84-7 | R_1_=R_2_=R_4_=H R_3_=OH (*trans*) | 228.0786 (C_14_H_12_O_3_) |  |  | 54247418/ [25](#_ENREF_25) |
| 2 | Piceatannol | 4339-71-3/ 10083-24-6 | R_1_=R_2_=H R_3_=R_4_=OH (*trans*) | 244.0736 (C_14_H_12_O_4_) | ESI^-^: 243.07 [M-H]^-^, 201.06, 159.045; EI^+^: 147, 133, 131 | 220, 303, 323 (log E = 4.41, 4.36, 4.48) | 667639/ [40](#_ENREF_40) |
| 3 | Astringin/ Piceatannol 3-*O*-glucoside | 29884-49-9 | R_1_=Glc R_2_=H R_3_=R_4_=OH (*trans*) | 406.1264 (C_20_H_22_O_9_) | ESI^+^: 406.126 [M]+/ 407.42 [M+H]^+^, 105.067, 244.064/ 245, 98.972; ESI^-^: 405 [M-H]^-^, 243.064, 241.05, 242.052 | 219, 303, 326 (log E = 4.53, 4.45, 4.54) | 5281712/ [40](#_ENREF_40) |
| 4 | Piceatannol 3'-*O*-*β*-D-xylopyranoside | 94356-27-1 | R_1_=R_2_=H R_3_=O-Xyl R_4_=OH (*trans*) | 376.1158 (C_19_H_20_O_8_) | ESI^+^: 376 [M]^+^, 244, 133 | 217, 305, 322 (log E = 4.46, 4.45, 4.49) | [40](#_ENREF_40) |
| 5 | Piceatannol 3'-*O*-glucoside | 94356-26-0 | R_1_=R_2_=H R_3_=O-Glc R_4_=OH (*trans*) | 406.1264 (C_20_H_22_O_9_) | ESI^+^: 406 [M]^+^, 244, 163 | 217, 302, 320 (log E = 4.38, 4.41, 4.48) | 11968990/ [40](#_ENREF_40) |
| 6 | Piceatannol 3'-*O*-*β*-D-(6''-*O*-galloyl)-glucopyranoside | 94356-28-2 | R_1_=R_2_=H R_3_=O-Glc^6^→O-G R_4_=OH (*trans*) | 558.1373 (C_27_H_26_O_13_) | ESI^+^: 558 [M]^+^, 406, 244, 170 |  | [40](#_ENREF_40) |
| 7 | Piceatannol 4'-*O*-glucoside | 116181-54-5 | R_1_=R_2_=H R_3_=OH R_4_=O-Glc (*trans*) | 406.1264 (C_20_H_22_O_9_) | ESI^+^: 406 [M]^+^, 244, 163; ESI^-^: 445.15 [M-H]^-^, 264.95, 403.05, 399.14/ 200.87, 324.99 | 305, 319 (log E = 4.39, 4.42) | 6481477/ [41](#_ENREF_41) |
| 8 | Piceatannol 4'-*O*-*β*-D-(6''-*O*-galloyl)-glucopyranoside | 116107-19-8 | R_1_=R_2_=H R_3_=OH R_4_=O-Glc^6^→O-G (*trans*) | 558.1373 (C_27_H_26_O_13_) | ESI^+^: 581 [M+Na]+/ 558 [M]^+^, 406, 243, 170 | 301, 322 (log E = 4.33, 4.31) | 14077412/ [41](#_ENREF_41) |
| 9 | Piceatannol 3,4'-diglucoside | 282736-93-0 | R_1_=Glc R_2_=H R_3_=OH R_4_=O-Glc (*trans*) | 568.1792 (C_26_H_32_O_14_) |  |  | [1](#_ENREF_1) |
| 10 | Resveratrol | 501-36-0 | R_1_=R_2_=R_3_=H R_4_=OH (*trans*) | 228.0786 (C_14_H_12_O_3_) | ESI^+^: 229.086 [M+H]^+^, 135.044, 107.049; ESI^-^: 227.07 [M-H]^-^, 185.06, 143.049/ 228.075; EI^+^: 444, 445, 147; ESI^-^(IT): 185, 227, 183 | 218, 307, 321 | 445154 |
| 11 | Polydatin/ Resveratrol 3-*O*-glucoside | 27208-80-6/ 65914-17-2 | R_1_=Glc R_2_=R_3_=H R_4_=OH (*trans*) | 390.1315 (C_20_H_22_O_8_) | ESI^+^: 391.137 [M+H]^+^, 229.083, 229.079, 229.081; ESI^-^: 389.124 [M-H]^-^, 227.071, 185.06, 143.049 | 219, 316 | 5281718/ [42](#_ENREF_42) |
| 12 | Resveratroloside/ Resveratrol 4'-*O*-glucoside | 38963-95-0 | R_1_=R_2_=R_3_=H R_4_=O-Glc (*trans*) | 390.1315 (C_20_H_22_O_8_) | ESI^+^: 229; ESI^-^: 389 [M-H]^-^, 227 | 276 | 5322089 |
| 13 | Resveratrol 4'-*O*-*β*-D-(2''-*O*-galloyl)-glucopyranoside |  | R_1_=R_2_=R_3_=H R_4_=O-Glc^2^→O-G (*trans*) | 542.1424 (C_27_H_26_O_12_) | ESI^+^: 542 [M]^+^, 390, 314, 228, 170, 153 |  | 10325054/ [43](#_ENREF_43) |
| 14 | Resveratrol 4'-*O*-*β*-D-(6''-*O*-galloyl)-glucopyranoside | 80446-63-5 | R_1_=R_2_=R_3_=H R_4_=O-Glc^6^→O-G (*trans*) | 542.1424 (C_27_H_26_O_12_) |  |  | 5322088 |
| 15 | [6-[4-[(*Z*)-2-(3,5-Dihydroxyphenyl)ethenyl]phenoxy]-3,4,5-trihydroxyoxan-2-yl]methyl 3,4,5-trihydroxybenzoate | 64898-03-9 | R_1_=R_2_=R_3_=H R_4_=O-Glc^6^→O-G (*cis*) | 542.1424 (C_27_H_26_O_12_) |  |  | 131751610/ 11800639/ TCMSP |
| 16 | 4'-Methoxyresveratrol/ Desoxyrhapontigenin | 33626-08-3 | R_1_=R_2_=R_3_=H R_4_=OCH_3_ (*trans*) | 242.0943 (C_15_H_14_O_3_) | ESI^+^: 243.145 [M+H]^+^, 107.03, 123.04, 147.04/ 171.09 | 214.8, 304.8 | 6255462/ [44](#_ENREF_44) |
| 17 | Rhapontigenin | 500-65-2 | R_1_=R_2_=H R_3_=OH R_4_=OCH_3_ (*trans*) | 258.0892 (C_15_H_14_O_4_) | ESI^+^(IT): 259.096 [M+H]^+^, 227.1, 135.1, 241.1 | 220, 302, 322 (log E = 4.41, 4.49, 4.53) | 5320954/ [40](#_ENREF_40) |
| 18 | 5-[(*Z*)-2-(3-Hydroxy-4-methoxyphenyl)ethenyl]benzene-1,3-diol | 500-65-2 | R_1_=R_2_=H R_3_=OH R_4_=OCH_3_ (*cis*) | 258.0892 (C_15_H_14_O_4_) | ESI^+^: 259.13 [M+H]^+^, 281.02, 250.34; ESI^-^: 257 [M-H]^-^ | 320 | 10879760 |
| 19 | Isorhapontigenin | 32507-66-7 | R_1_=R_2_=H R_3_=OCH_3_ R_4_=OH (*trans*) | 258.0892 (C_15_H_14_O_4_) | ESI^+^: 259.096 [M+H]^+^, 181.064, 153.071, 152.061; ESI^-^: 257.082 [M-H]^-^, 241.050, 242.056, 255.233 |  | 5318650 |
| 20 | Rhapontigenin 3'-*O*-glucoside | 94356-22-6 | R_1_=R_2_=H R_3_=O-Glc R_4_=OCH_3_ (*trans*) | 420.1420 (C_21_H_24_O_9_) | ESI^+^: 420 [M]^+^, 258, 163 | 217, 303, 330 (log E = 4.4, 4.43, 4.51) | 45033634/ [40](#_ENREF_40) |
| 21 | Desoxyrhaponticin | 30197-14-9 | R_1_=Glc R_2_=R_3_=H R_4_=OCH_3_ (*trans*) | 404.1471 (C_21_H_24_O_8_) | ESI^+^: 404 [M]^+^, 242 | 215, 306, 319 (log E = 4.4, 4.52, 4.52) | 5316606/ |
| 22 | (*Z*)-4'-*O*-Methylpiceid |  | R_1_=Glc R_2_=R_3_=H R_4_=OCH_3_ (*cis*) | 404.1471 (C_21_H_24_O_8_) | ESI^+^: 443 [M+K]+/ 404 [M]^+^, 163 | 215, 284 (log E = 4.3, 4.04) | 11795500/ [40](#_ENREF_40) |
| 23 | Desoxyrhaponticin 6''-*O*-gallate | 94356-29-3 | R_1_=Glc^6^→O-G R_2_=R_3_=H R_4_=OCH_3_ (*trans*) | 556.1581 (C_28_H_28_O_12_) | ESI^+^: 579 [M+Na]^+^, 565, 405, 278 |  | 145972871/ [40](#_ENREF_40) |
| 24 | Rhapontin | 155-58-8 | R_1_=Glc R_2_=H R_3_=OH R_4_=OCH_3_ (*trans*) | 420.1420 (C_21_H_24_O_9_) | ESI^+^: 420 [M]^+^, 258 | 219, 302, 324 (log E = 4.4, 4.38, 4.48) | 637213/ |
| 25 | (*Z*)-Rhaponticin |  | R_1_=Glc R_2_=H R_3_=OH R_4_=OCH_3_ (*cis*) | 420.1420 (C_21_H_24_O_9_) | ESI^+^: 443 [M+Na]+/ 420 [M]^+^, 404, 163 | 216, 288 (log E = 4.46, 4.08) | 101535997/ [40](#_ENREF_40) |
| 26 | Isorhapontin | 32727-29-0 | R_1_=Glc R_2_=H R_3_=OCH_3_ R_4_=OH (*trans*) | 420.1420 (C_21_H_24_O_9_) | ESI^+^: 420.142 [M]+/ 421.46 [M+H]^+^, 258.091, 197.062, 169.066/ 259, 421, 260; ESI^-^: 419.135 [M-H]^-^, 257.081, 241.050, 242.058 |  | 5281716 |
| 27 | Rhaponticin 2''-*O*-gallate | 94356-24-8 | R_1_=Glc^2^→O-G R_2_=H R_3_=OH R_4_=OCH3 (*trans*) | 572.1530 (C_28_H_28_O_13_) | ESI^+^: 572 [M]^+^, 420, 258, 153 |  | 10325743/ [40](#_ENREF_40) |
| 28 | (*Z*)-Rhaponticin 2''-*O*-gallate |  | R_1_=Glc^2^→O-G R_2_=H R_3_=OH R_4_=OCH_3_ (*cis*) | 572.1530 (C_28_H_28_O_13_) |  |  | [40](#_ENREF_40) |
| 29 | Rhaponticin 6''-*O*-gallate | 94356-23-7 | R_1_=Glc^6^→O-G R_2_=H R_3_=OH R_4_=OCH_3_ (*trans*) | 572.1530 (C_28_H_28_O_13_) | ESI^+^: 572 [M]^+^, 420, 258, 153 |  | 10076782/ [40](#_ENREF_40) |
| 30 | (*Z*)-Rhaponticin 6''-*O*-gallate |  | R_1_=Glc^6^→O-G R_2_=H R_3_=OH R_4_=OCH_3_ (*cis*) | 572.1530 (C_28_H_28_O_13_) | ESI^+^: 595 [M+Na]^+^, 572, 420, 258 | 217, 280 (log E = 4.46, 4.04) | [40](#_ENREF_40) |
| 31 | Rhaponticin 2''-*O*-*p*-coumarate | 94356-25-9 | R_1_=Glc^2^→O-*p*-Coum R_2_=H R_3_=OH R_4_=OCH_3_ (*trans*) | 566.1788 (C_30_H_30_O_11_) | ESI^+^: 566 [M]^+^, 420, 283 | 220, 302, 315 (log E = 4.32, 4.43, 4.49) | [40](#_ENREF_40) |
| 32 | 2-[4-[(*E*)-2-(3,5-Dimethoxyphenyl)ethenyl]phenoxy]-6-(hydroxymethyl)oxane-3,4,5-triol | 38967-99-6 | R_1_=R_2_=CH_3_ R_3_=H R_4_=O-Glc (*trans*) | 418.1628 (C_22_H_26_O_8_) |  |  | 52948243/ [1](#_ENREF_1) |
| 33 | (2*S*,3*R*,4*S*,5*R*,6*R*)-2-[3-[(*E*)-2-(3,4-Dimethoxyphenyl)ethenyl]-5-methoxyphenoxy]-3,4,5-trimethoxy-6-(methoxymethyl)oxane | 266997-55-1 | R_1_= R_2_=CH_3_ R_3_=R_4_=OCH_3_ (*trans*) | 504.2359 (C_27_H_36_O_9_) |  |  | 44339901/ [1](#_ENREF_1) |
| 34 | (2*S*,3*R*,4*S*,5*R*,6*R*)-2-[5-[(*E*)-2-(3,5-Dimethoxyphenyl)ethenyl]-2-methoxyphenoxy]-3,4,5-trimethoxy-6-(methoxymethyl)oxane | 266997-56-2 | R_1_=R_2_=CH_3_  R_3_=O R_4_=OCH_3_ (*trans*) | 504.2359 (C_27_H_36_O_9_) |  |  | 44339880/ [1](#_ENREF_1) |
| 35 | [3,4,5-Triacetyloxy-6-[4-[(*E*)-2-(3,5-dimethoxyphenyl)ethenyl]phenoxy]oxan-2-yl]methyl acetate | 38968-00-2 | R_1_=R_2_=CH_3_ R_3_=H R_4_=O (*trans*) | 586.2050 (C_30_H_34_O_12_) |  |  | 69198447/ [1](#_ENREF_1) |
| 36 | [(2*R*,3*R*,4*S*,5*R*,6*S*)-3,4,5-Triacetyloxy-6-[4-[(*E*)-2-(3,5-diacetyloxyphenyl)ethenyl]phenoxy]oxan-2-yl]methyl acetate | 38968-01-3 | R_1_=R_2_=COCH_3_ R_3_=H R_4_=O (*trans*) | 642.1949 (C_32_H_34_O_14_) |  |  | 11273707/ [1](#_ENREF_1) |
|  | | | | | | | |
| 37 | Gnetin C | 84870-54-2 |  | 454.1416 (C_28_H_22_O_6_) | ESI^+^: 454 [M]^+^, 43, 58, 437, 360, 347, 331, 239, 227, 213, 181, 157, 121, 107, 94, 83 | 222, 285, 308, 325 (log E = 4.66, 4.31, 4.48, 4.51) | 21633857/ [45](#_ENREF_45) |
| 38 | Maximol A |  |  | 454.1416 (C_28_H_22_O_6_) | ESI^+^: 455.142 [M+H]^+^ | 310, 211 (log E = 4.6, 4.8) | 637098/ [46](#_ENREF_46) |
| 39 | Maximol B |  |  | 454.1416 (C_28_H_22_O_6_) | ESI^-^: 453.131 [M-H]^-^ | 298, 205 (log E = 4.5, 4.9) | 101093543/ [46](#_ENREF_46) |
| 1. Naphthalene glycosides | | | | | | | |
| 1 | 1-[1,6-Dihydroxy-3-methyl-8-[(2*S*,3*R*,4*S*,5*S*,6*R*)-3,4,5-trihydroxy-6-(hydroxymethyl)oxan-2-yl]oxynaphthalen-2-yl]ethanone | 23566-96-3 | R_1_=R_2_=R_3_=R_4_=R_5_=R_6_=H | 394.1264 (C_19_H_22_O_9_) |  |  | 100753 |
| 2 | Torachrysone 8-*O*-glucoside | 64032-49-1 | R_1_=R_3_=R_4_=R_5_=R_6_=H R_2_=CH_3_ | 408.1420 (C_20_H_24_O_9_) | ESI^+^: 409 [M+H]^+^, 247, 229; ESI^-^: 407 [M-H]^-^, 245 | 235, 240, 260, 270, 314, 326, 340 (log E = 4.74, 4.7, 4.49, 4.38, 3.39, 3.91, 3.71) | 11972479/ [47](#_ENREF_47) |
| 3 | Torachrysone 8-(6-oxalylglucoside) | 64078-76-8 | R_1_=R_3_=R_4_=R_5_=H R_2_=CH_3_ R_6_=COCOOH | 480.1268 (C_22_H_24_O_12_) |  | 235, 262, 311, 325, 340 | 78385603/ [47](#_ENREF_47) |
| 4 | 1-(1,6-Dimethoxy-3-methyl-8-((3,4,5-trihydroxy-6-(hydroxymethyl)tetrahydro-2*H*-pyran-2-yl)oxy)naphthalen-2-yl)ethan-1-one | 65222-45-9 | R_1_=R_2_=CH_3_ R_3_=R_4_=R_5_=R_6_=H | 422.1577 (C_21_H_26_O_9_) |  |  | [1](#_ENREF_1) |
| 5 | 2-((8-Acetoxy-7-acetyl-3-methoxy-6-methylnaphthalen-1-yl)oxy)-6-(acetoxymethyl)tetrahydro-2*H*-pyran-3,4,5-triyl triacetate | 65222-44-8 | R_1_=R_3_=R_4_=R_5_=R_6_=COCH_3_ R_2_=CH_3_ | 618.1949 (C_30_H_34_O_14_) |  |  | [1](#_ENREF_1) |
| 6 | 2-(Acetoxymethyl)-6-((3,8-diacetoxy-7-acetyl-6-methylnaphthalen-1-yl)oxy)tetrahydro-2*H*-pyran-3,4,5-triyl triacetate | 23600-01-3 | R_1_=R_2_=R_3_=R_4_=R_5_=R_6_=COCH_3_ | 646.1898 (C_31_H_34_O_15_) |  |  | [1](#_ENREF_1) |
|  | | | | | | | |
| 7 | Toralactone | 41743-74-2 |  | 272.0685 (C_15_H_12_O_5_) |  |  | 5321980/ TCMSP |
| 1. Butyrylbenzene glycosides | | | | | | | |
| 1 | Raspberryketone glucoside | 38963-94-9 | R_1_=R_2_=R_3_=R_4_=H | 326.1365 (C_16_H_22_O_7_) | ESI^+^: 327 [M+H]^+^, 107; ESI^-^: 325 [M-H]^-^ | 271 | 5320521 |
| 2 | Raspberry ketone tetra-*O*-acetyl-*β*-D-glucopyranoside | 38963-96-1 | R_1_=R_2_=R_3_=R_4_=COCH_3_ | 494.1788 (C_24_H_30_O_11_) |  |  | [1](#_ENREF_1) |
| 3 | Lindleyin | 59282-56-3 | R_1_=R_2_=R_3_=H R_4_=G | 478.1475 (C_23_H_26_O_11_) | ESI^+^: 479 [M+H]^+^, 107, 43, 153, 164, 94, 170, 91, 460.138; ESI^-^: 477 [M-H]^-^ | 230, 282 (log E = 4.38, 4.33) | 42994/ [48](#_ENREF_48) |
| 4 | Isolindleyin | 87075-18-1 | R_1_=G R_2_=R_3_=R_4_=H | 478.1475 (C_23_H_26_O_11_) |  |  | 10390322/ [29](#_ENREF_29) |
| 5 | Isolindleyin trimethyl ether | 87087-63-6 | R_1_=  R_2_=R_3_=R_4_=H | 520.1945 (C_26_H_32_O_11_) |  |  | [1](#_ENREF_1) |
| 6 | 4-(4-Hydroxyphenyl)-2-butanone *O*-[2,6-digalloylglucoside] | 105274-14-4 | R_1_=R_4_=G R_2_=R_3_=H | 630.1585 (C_30_H_30_O_15_) | ESI^+^: 669 [M+K]+/ 653 [M+Na]+/ 630 [M]^+^, 517, 501, 164 |  | 14345585/ [43](#_ENREF_43) |
| 7 | 4-(4-Hydroxyphenyl)-2-butanone *O*-[2-galloyl-6-cinnamoylglucoside] | 105274-15-5 | R_1_=G R_2_=R_3_=H R_4_=Cin | 608.1894 (C_32_H_32_O_12_) | ESI^+^: 647 [M+K]+/ 631 [M+Na]+/ 609 [M+H]^+^, 445, 164 |  | 14345589/ [43](#_ENREF_43) |
| 8 | 4-(4-Hydroxyphenyl)-2-butanone *O*-[2-galloyl-6-*p*-coumaroylglucoside] | 105274-16-6 | R_1_=G R_2_=R_3_=H R_4_=*p*-Coum | 624.1843 (C_32_H_32_O_13_) | ESI^+^: 624 [M]^+^, 472, 460, 314, 164 |  | 14345587/ [43](#_ENREF_43) |
|  | | | | | | | |
| 9 | 4-(4-Hydroxyphenyl)-2-butanone | 5471-51-2 |  | 164.0837 (C_10_H_12_O_2_) | EI^+^: 107, 164, 77 | 224, 239 | 21648/ [29](#_ENREF_29) |
| 1. Chromones | | | | | | | |
| 1 | 2,5-Dimethyl-7-hydroxychromone | 38412-47-4 | R_1_=R_2_=CH_3_ R_3_=H | 190.0630 (C_11_H_10_O_3_) | ESI^+^: 190 [M]^+^, 161, 122; EI^+^: 190, 150, 122 | 241, 250, 289 (log E = 4.47, 4.5, 4.28) | 5316891/ |
| 2 | 5-Acetyl-7-hydroxy-2-methyl-chromone | 94356-33-9 | R_1_=CH_3_ R_2_=COCH_3_ R_3_=H | 218.0579 (C_12_H_10_O_4_) |  |  | 5315891/ TCMSP |
| 3 | 7-Hydroxy-2-methyl-4-oxo-4*H*-1-benzopyran-5-carboxylic acid |  | R_1_=CH_3_ R_2_=COOH R_3_=H | 220.0372 (C_11_H_8_O_5_) |  |  | 5315688/ TCMSP |
| 4 | Cassiachromone | 28955-30-8 | R_1_=CH_3_ R_2_=CH_2_COCH_3_ R_3_=H | 232.0736 (C_13_H_12_O_4_) | ESI^+^: 232.073 [M]^+^, 217, 215, 190, 161, 150, 122 | 243, 250, 291 (log E = 4.32, 4.34, 4.21) | 5319500/ [49](#_ENREF_49) |
| 5 | 7-Hydroxy-2-methyl-4-oxo-4*H*-1-benzopyran-5-acetic acid | 94356-34-0 | R_1_=CH_3_ R_2_=CH_2_COOH R_3_=H | 234.0528 (C_12_H_10_O_5_) | ESI^+^: 234 [M]^+^, 218, 190, 176, 150, 122, 120 | 242, 249, 290 (log E = 4.31, 4.32, 5.05) | 14429402/ [49](#_ENREF_49) |
| 6 | Aloesol | 94356-35-1 | R_1_=CH_2_CHOHCH_3_ R_2_=CH_3_ R_3_=H | 234.0892 (C_13_H_14_O_4_) | ESI^+^: 234 [M]^+^, 190, 161, 151, 124, 122 | 243, 250, 289 (log E = 4.35, 4.37, 4.19) | 5318230/ [49](#_ENREF_49) |
| 7 | Aloesol 7-glucoside | 94356-36-2 | R_1_=CH_2_CHOHCH_3_ R_2_=CH_3_ R_3_=Glc | 396.1420 (C_19_H_24_O_9_) | ESI^+^: 419 [M+Na]+/ 397 [M+H]^+^, 379, 234, 163 |  | 131752856/ [49](#_ENREF_49) |
| 8 | 2'-Oxoaloesol 7-glucoside | 128701-04-2 | R_1_=CH_2_COCH_3_ R_2_=CH_3_ R_3_=Glc | 394.1264 (C_19_H_22_O_9_) | ESI^+^: 417 [M+Na]+/ 395 [M+H]^+^, 232, 163 | 242, 249, 278 (log E = 4.54, 4.52, 4.29) | 14524532/ [50](#_ENREF_50) |
| 9 | 7-Hydroxy-5-(4-hydroxy-2-oxopentyl)-2-methylchromone |  | R_1_=CH_3_ R_2_=CH_2_COCH_2_CHOHCH_3_ R_3_=H | 276.0998 (C_15_H_16_O_5_) | ESI^+^: 276 [M]^+^, 190, 232, 258, 217, 161, 150, 122 |  |  |
| 10 | 7-Hydroxy-5-(4-hydroxy-2-oxopentyl)-2-methylchromone 7-glucoside | 128701-05-3 | R_1_=CH_3_ R_2_=CH_2_COCH_2_CHOHCH_3_ R_3_=Glc | 438.1526 (C_21_H_26_O_10_) | ESI^+^: 439 [M+H]^+^, 277 | 240, 248, 278 (log E = 4.4, 4.35, 4.27) | 14524534/ [50](#_ENREF_50) |
|  | | | | | | | |
| 11 | Kaempferol | 520-18-3 | R_1_=OH R_2_=R_3_=R_4_=R_5_=R_6_=H | 286.0477 (C_15_H_10_O_6_) | ESI^+^: 287.05 [M+H]^+^, 287.146, 202.058; ESI^-^: 285.041/ 285.034 [M-H]^-^, 151.003, 257.046/ 187.035, 159.043; EI^+^: 286, 285, 69; ESI^-^(IT): 285, 213, 229 | 265, 365 | 5280863 |
| 12 | Afzelin | 482-39-3 | R_1_=O-Rha R_2_=R_3_=R_4_=R_5_=R_6_=H | 432.1056 (C_21_H_20_O_10_) | ESI^+^: 433.113/ 433.37 [M+H]^+^, 287.055, 288.058, 85.027/ 147, 129; ESI^-^: 477.104 [M+FA-H]^-^, 285.041, 431.1, 284.033 |  | 5316673 |
| 13 | Kaempferol 3-*O*-(2'',6''-di-*O*-rhamnopyranosyl)glucopyranoside | 55804-74-5 | R_1_=O-Glc^2,6^→O-Rha R_2_=R_3_=R_4_=R_5_=R_6_=H | 740.2164 (C_33_H_40_O_19)_ |  |  | 123132000 |
| 14 | Quercetin | 117-39-5 | R_1_=R_5_=OH R_2_=R_3_=R_4_=R_6_=H | 302.0426 (C_15_H_10_O_7_) | ESI^+^: 303.05 [M+H]^+^, 304.051, 229.048; ESI^-^: 301 [M-H]^-^, 245.045, 152.018, 150.997/ 178.914, 150.926; EI^+^: 559, 560, 133; ESI^+^(IT): 257, 229, 285 | 256, 301, 373 (log E = 4.32, 3.89, 4.32) | 5280343/ |
| 15 | Quercitrin | 522-12-3 | R_1_=O-Rha R_2_=R_3_=R_4_=R_6_=H R_5_=OH | 448.1006 (C_21_H_20_O_11_) | ESI^+^: 449.11 [M+H]^+^, 287.05, 287.058/ 303.043, 272.056; ESI^-^: 447.1 [M-H]^-^, 300.029, 301.04/ 300.039, 271.033; ESI^-^(IT): 301.1, 300.1, 284.1 | 350, 258 (log E = 4.18, 4.3) | 5280459/ [25](#_ENREF_25) |
| 16 | Isoquercitrin | 482-35-9 | R_1_=O-Glc R_2_=R_3_=R_4_=R_6_=H R_5_=OH | 464.0955 (C_21_H_20_O_12_) | ESI^+^: 465 [M+H]^+^, 303, 85.029, 97.029/ 464, 302, 145; ESI^-^: 463.088 [M-H]^-^, 300.027, 301.035 |  | 5280804/ [25](#_ENREF_25) |
| 17 | Peltatoside | 23284-18-6 | R_1_=O-Glc^6^→O-Ara R_2_=R_3_=R_4_=R_6_=H R_5_=OH | 596.1377 (C_26_H_28_O_16_) | ESI^+^: 597 [M+H]^+^, 303, 598/ 596, 302; ESI^-^: 595.13 [M-H]^-^, 300.026, 301.034 |  | 5484066/ [25](#_ENREF_25) |
| 18 | Rutin | 153-18-4 | R_1_=O-Glc^6^→O-Rha R_2_=R_3_=R_4_=R_6_=H R_5_=OH | 610.1534 (C_27_H_30_O_16_) | ESI^+^: 611.16 [M+H]^+^, 303.049, 85.029, 71.05; ESI^-^: 609.15 [M-H]^-^, 300.03, 301.03, 299.018/ 610.151, 1219.312; ESI^-^(IT): 301.1, 300.1, 343.1 |  | 5280805 |
| 19 | Eupatin | 19587-65-6 | R_1_=R_5_=OH R_2_=OCH_3_ R_3_=R_6_=CH_3_ R_4_=H | 360.0845 (C_18_H_16_O_8_) |  |  | 5317287/ TCMSP |
| 20 | Vitexin | 3681-93-4 | R_1_=R_2_=R_3_=R_5_=R_6_=H R_4_=Glc | 432.1056 (C_21_H_20_O_10_) | ESI^+^: 433.11 [M+H]^+^, 313.07, 415.1/ 397.092; ESI^-^: 431.098 [M-H]^-^, 311.056, 283.06/ 312.059; ESI^+^(IT): 415.1, 397.1, 367.1; ESI^-^(IT): 311, 341, 283 |  | 5280441 |
|  | | | | | | | |
| 21 | (*S*)-2,3-Dihydro-7-hydroxy-2-methyl-4-oxo-4*H*-1-benzopyran-5-acetic acid | 94356-37-3 |  | 236.0685 (C_12_H_12_O_5_) | ESI^+^: 236 [M]^+^, 192, 190, 176, 166, 150, 122; EI^+^: 150, 190, 192 |  | 591742/ 5319543/ [49](#_ENREF_49) |
| 22 | 3-[(*E*)-3-(3,5-Dihydroxyphenyl)prop-2-enoyl]-5-hydroxy-3,4-dihydropyran-2-one |  |  | 276.0634 (C_14_H_12_O_6_) | ESI^+^: 276.063 [M]^+^, 258, 248, 232, 189, 173, 161, 143, 131, 115, 103, 91, 77, 63 |  | 5320968/ [51](#_ENREF_51) |
| 1. Others | | | | | | | |
| 1 | Glucose | 2280-44-6/ 50-99-7 | I | 180.0634 (C_6_H_12_O_6_) | ESI^+^: 98.64, 98.48/ 80.66, 98.45/ 217.108, 293.194; ESI^-^: 179.063 [M-H]^-^, 59.013, 71.014, 89.026; EI^+^: 147, 205, 103 |  | 5793/ [25](#_ENREF_25) |
| 2 | Pectin | 18968-14-4/ 9000-69-5 | I | 194.0426 (C_6_H_10_O_7_) | ESI^+^: 76/ 87, 89, 69 |  | 441476/ [25](#_ENREF_25) |
| 3 | Starch | 9005-25-8 | I | 692.2739 (C_27_H_48_O_20_) |  |  | 24836924/ [25](#_ENREF_25) |
| 4 | Cellulose | 9004-34-6/ 9013-34-7/ 99331-82-5 | I | (162.0528)_n_ (C_6_H_10_O_5_)_n_ |  |  | 16211032/ [25](#_ENREF_25) |
| 5 | 2-Methyl-5-[(8*Z*,11*Z*)-pentadeca-8,11-dienyl]benzene-1,3-diol | 50423-14-8 | II | 330.2559 (C_22_H_34_O_2_) |  |  | 5319544/ TCMSP |
| 6 | 2'-Methylacetophenone | 577-16-2/ 26444-19-9 | III | 134.0732 (C_9_H_10_O) | EI^+^: 91, 119, 134 |  | 11340/ TCMSP |
| 7 | Benzylideneacetone | 122-57-6/ 1896-62-4 | III | 146.0732 (C_10_H_10_O) | ESI^+^: 147.08 [M+H]^+^, 129.07, 128.062; EI^+^: 131, 103, 146 |  | 637759/ TCMSP |
| 8 | (8*S*,9*S*,10*R*,13*S*,14*R*,17*S*)-17-Acetyl-10,13-dimethyl-1,2,6,7,8,9,11,12,14,15,16,17-dodecahydrocyclopenta[a]phenanthren-3-one | 57-83-0 | III | 314.2246 (C_21_H_30_O_2_) |  |  | 11876100/ TCMSP |
| 9 | 10*β*-Hydroxy-6*β*-isobutyrylfuranoeremophilane | 60410-89-1 | III | 320.1988 (C_19_H_28_O_4_) |  |  | 442377/ TCMSP |
| 10 | Tripterygone | 138570-50-0 | III | 454.3083 (C_29_H_42_O_4_) |  |  | 197388/ TCMSP |
| 11 | Rhapontisterone | 137476-71-2 | III | 496.3036 (C_27_H_44_O_8_) |  |  | 11799446/ TCMSP |
| 12 | 1-Cinnamoyl-3-hydroxy-11-methoxymeliacarpinin | 177795-22-1 | III | 696.2782 (C_37_H_44_O_13_) |  |  | 137324729/ TCMSP |
| 13 | Oxalic acid | 144-62-7 | IV | 89.9953 (C_2_H_2_O_4_) | ESI^+^: 89.55 [M]^+^, 38/ 73.1, 52.7, 52.8/ 69, 68, 89; EI^+^: 147, 148, 149 |  | 971/ [25](#_ENREF_25) |
| 14 | Lactic acid | 50-21-5/ 598-82-3/ 26100-51-6/ 92129-90-3/ 31587-11-8 | IV | 90.0317 (C_3_H_6_O_3_) | ESI^-^: 89.024 [M-H]^-^; EI^+^: 147, 117, 148 | 240 | 612/ [25](#_ENREF_25) |
| 15 | Maleic acid/ *cis*-Butenedioic acid | 110-16-7/ 68307-91-5 | IV | 116.0110 (C_4_H_4_O_4_) | ESI^+^: 117.07 [M+H]^+^, 99, 117, 98; ESI^-^: 115 [M-H]^-^, 71.01, 115.002, 97.334; EI^+^: 147, 148, 245; ESI^-^(IT): 71, 71.8 | 210 (log E = 4.14) | 444266/ [25](#_ENREF_25) |
| 16 | Fumaric acid/ *trans*-Butenedioic acid | 110-17-8/ 6915-18-0 | IV | 116.0110 (C_4_H_4_O_4_) | ESI^-^: 115 [M-H]^-^, 71.01, 114.901/ 72.017; EI^+^: 147, 245, 143 | 208 (log E = 4.2) | 444972/ [25](#_ENREF_25) |
| 17 | Succinic acid | 110-15-6 | IV | 118.0266 (C_4_H_6_O_4_) | ESI^+^: 119.034 [M+H]^+^, 118.742; ESI^-^: 117.02 [M-H]^-^, 73.03, 117.02, 99.01; EI^+^: 147, 148, 247; ESI^-^(IT): 73, 117, 99 | 208 (log E = 2) | 1110/ [25](#_ENREF_25) |
| 18 | Malic acid | 6915-15-7/ 617-48-1/ 636-61-3/ 97-67-6 | IV | 134.0215 (C_4_H_6_O_5_) | ESI^-^: 133.01 [M-H]^-^, 72.99/ 114.952, 132.909, 71.104/ 115.02, 71.013, 115.009, 71.993; EI^+^: 147, 232.95, 133; ESI^-^(IT): 115, 87.1, 71.1 |  | 525/ 92824/ 222656/ [25](#_ENREF_25) |
| 19 | Cinnamic acid | 140-10-3/ 621-82-9/ 102-94-3 | IV | 148.0524 (C_9_H_8_O_2_) | ESI^+^: 149.19 [M+H]+/ 319.094 [2M+Na]^+^, 149, 148; ESI^-^: 147.04 [M-H]^-^, 119.049, 101.038/ 103.051, 77.04, 77.033; EI^+^: 103, 131, 161; ESI^+^(IT): 149.06, 131.1, 131.9 | 205, 273 | 444539/ 5372954/ |
| 20 | Citric acid | 77-92-9/ 10024-66-5 | IV | 192.0270 (C_6_H_8_O_7_) | ESI^-^: 191.019 [M-H]^-^, 111.01, 87.01, 85.03; EI^+^: 147, 273, 133; ESI^+^(IT): 193.034 [M+H]^+^, 175, 147.1, 170.1 |  | 311/ [25](#_ENREF_25) |
| 21 | Lauric acid | 143-07-7/ 203714-07-2/ 7632-48-6/ 8000-62-2 | IV | 200.1776 (C_12_H_24_O_2_) | ESI^-^: 199.2 [M-H]^-^, 43, 55, 57; EI^+^: 117, 129, 132; ESI^+^(IT): 201.185 [M+H]^+^, 103.1, 89.1, 117.1 |  | 3893 |
| 22 | Myristic acid | 544-63-8/ 32112-52-0 | IV | 228.2089 (C_14_H_28_O_2_) | ESI^-^: 227 [M-H]^-^, 209.064, 45.335/ 158.985, 130.974; EI^+^: 117, 129, 132; ESI^-^(IT): 113.1, 209.3, 227.3 | 210 (log E = 1.85) | 11005 |
| 23 | Pentadecanoic acid | 1002-84-2 | IV | 242.2246 (C_15_H_30_O_2_) | ESI^-^: 241.22 [M-H]^-^, 242.22, 243.222/ 223.207/ 171.14, 172.143, 365.267; EI^+^: 117, 129, 132; ESI^-^(IT): 223.3, 241.4 |  | 13849 |
| 24 | Palmitic acid | 57-10-3/ 67701-02-4 | IV | 256.2402 (C_16_H_32_O_2_) | ESI^-^: 255.2 [M-H]^-^, 113.096, 167.143/ 201.836, 219.845/ 186.94, 71.028; EI^+^: 117, 129, 132; ESI^-^(IT): 237.3, 255.3, 227.1 | 210 (log E = 1.7) | 985 |
| 25 | Tripalmitin | 555-44-2 | IV | 806.7363 (C_51_H_98_O_6_) | ESI^+^: 824.7 [M+NH_4_]^+^, 551.46, 552.47, 553.492/ 550.457/ 488.395, 127.117, 327.285, 57.071; EI^+^: 239, 552, 313 |  | 11147/ TCMSP |
| 26 | Linoleic acid | 60-33-3/ 506-21-8/ 2197-37-7/ 80969-37-5/ 8024-22-4 | IV | 280.2402 (C_18_H_32_O_2_) | ESI^-^: 279.2 [M-H]^-^, 96.959, 278.371/ 279.258, 280.262, 59.012; EI^+^: 95, 82, 96; ESI^+^(IT): 281.247 [M+H]^+^, 263.1, 265.1, 281.1 | 232, 275 (log E = 3.8, 2.9) | 5280450 |
| 27 | 5-(*β*-D-Glucopyranosyloxy)-2-hydroxybenzoic acid | 1820-89-9 | IV | 316.0794 (C_13_H_16_O_9_) |  |  | 10914066/ TCMSP |
| 28 | Docosanoic acid | 112-85-6 | IV | 340.3341 (C_22_H_44_O_2_) | ESI^+^: 341 [M+H]^+^, 342, 277/ 105, 77, 57; ESI^-^: 339.33 [M-H]^-^, 340.331, 341.333; EI^+^: 117, 129, 132; ESI^-^(IT): 321.4, 322.5, 295.2 |  | 8215/ TCMSP |
| 29 | Paeonol | 552-41-0 | V | 166.0630 (C_9_H_10_O_3_) | ESI^+^: 167.07 [M+H]^+^, 149.06, 121.066; ESI^-^: 165 [M-H]^-^, 135, 122, 91; EI^+^: 151, 166, 152/ 223, 180, 224; ESI^+^(IT): 149.2, 125.2, 126.2 |  | 11092/ [25](#_ENREF_25) |
| 30 | Methyleugenol | 93-15-2 | V | 178.0994 (C_11_H_14_O_2_) | EI^+^: 178, 91, 103 | 230, 280 (log E = 3.8, 3.4) | 7127/ [25](#_ENREF_25) |
| 31 | (+)-δ-Cadinene | 483-76-1 | V | 204.1878 (C_15_H_24_) | EI^+^: 161, 134, 204 |  | 441005/ [25](#_ENREF_25) |
| 32 | *β*-Sitosterol | 83-46-5/ 68555-08-8 | VI | 414.3862 (C_29_H_50_O) | ESI^+^: 415.153 [M+H]^+^, 81.1, 95.079, 57.189/ 108.99; EI^+^: 55, 57, 69 |  | 222284/ TCMSP |
| 33 | Strumaroside |  | VI | 562.4233 (C_34_H_58_O_6_) |  |  | 73946795/ TCMSP |
| 34 | Sitogluside/ Daucosterol | 474-58-8 | VI | 576.4390 (C_35_H_60_O_6_) | ESI^+^: 576 [M]^+^, 414, 399 |  | 5742590/ [3](#_ENREF_3) |
| 35 | Serotonin | 50-67-9 | VII | 176.0950 (C_10_H_12_N_2_O) | ESI^+^: 177.102 [M+H]^+^, 160.076, 161.079/ 132.081, 115.054; EI^+^: 146, 147, 45; ESI^+^(IT): 177, 160, 161 |  | 5202/ TCMSP |
| 36 | Glutamic acid | 56-86-0/ 6106-04-3 | VIII | 147.0532 (C_5_H_9_NO_4_) | ESI^+^: 148.06 [M+H]^+^, 130.05, 84.04/ 56.049, 72.044; EI^+^: 246.1, 128.1, 147.15 |  | 33032/ [25](#_ENREF_25) |
| 37 | Arginine | 74-79-3/ 4455-52-1 | VIII | 174.1117 (C_6_H_14_N_4_O_2_) | ESI^+^: 175.12 [M+H]^+^, 112.087, 70.065; EI^+^: 102, 147, 142; ESI^+^(IT): 158.1, 157.1, 116.1 | 205 | 6322/ [25](#_ENREF_25) |
| 38 | Citroxanthin/ Mutatochrome | 515-06-0 | IX | 552.4331 (C_40_H_56_O) |  |  | 5281246/ TCMSP |
| 39 | Vitamin B_1_ | 59-43-8 | IX | 300.0812 (C_12_H_17_ClN_4_OS) |  |  | 6042/ [25](#_ENREF_25) |
| 40 | Vitamin B_2_/ Riboflavin | 83-88-5 | IX | 376.1383 (C_17_H_20_N_4_O_6_) | ESI^+^: 377.1 [M+H]^+^, 243.1, 69.034/ 377.187, 99.048; ESI^-^: 375.131 [M-H]^-^, 255.089, 212.083, 241.073; EI^+^: 242, 171, 256; ESI^+^(IT): 243, 359, 282 | 267, 373, 447 (log E = 4.51, 4.02, 4.09) | 493570/ [25](#_ENREF_25) |
| 41 | Vitamin B_6_/ Pyridoxine | 65-23-6/ 8059-24-3 | IX | 169.0739 (C_8_H_11_NO_3_) | ESI^+^: 170.081 [M+H]^+^, 152.07, 170.096/ 134.06, 124.075; ESI^-^: 168.066 [M-H]^-^, 150.055, 122.06, 166.05; EI^+^: 280, 147, 295; ESI^+^(IT): 152.1, 153.3 | 214, 292 | 1054/ [25](#_ENREF_25) |
| 42 | Vitamin D/ Cholecalciferol | 67-97-0/ 1406-16-2 | IX | 384.3392 (C_27_H_44_O) | ESI^+^: 385.6 [M+H]^+^, 385, 386; ESI^+^(IT): 385.346, 367, 259, 273/ 255 | 264.5, 373 | 5280795/ [25](#_ENREF_25) |
| 43 | Gibberellin A_1_ | 545-97-1 | IX | 348.1573 (C_19_H_24_O_6_) | ESI^-^: 347 [M-H]^-^, 346.478, 347.575/ 273.033, 259.097 |  | 5280379/ |

**References**

**Table S3**

Relative contents of 108 chemical components from rhubarb samples (*n* = 3).

| Component No. | Relative contents | | | | | | | | | | | | | | | | | |
| --- | --- | --- | --- | --- | --- | --- | --- | --- | --- | --- | --- | --- | --- | --- | --- | --- | --- | --- |
|  | water-S | water-L | 10%-S | 10%-L | 20%-S | 20%-L | 35%-S | 35%-L | 50%-S | 50%-L | 65%-S | 65%-L | 80%-S | 80%-L | 90%-S | 90%-L | ethanol-S | ethanol-L |
| **1** | 38.86423 | 32.29187 | 26.76653 | 21.26613 | 15.59970 | 15.37447 | 13.52907 | 10.06610 | 10.68358 | 7.73231 | 7.55390 | 8.31943 | 7.50436 | 7.69769 | 7.26974 | 8.72577 | 7.11476 | 7.58946 |
| **2** | 16.88187 | 21.31763 | 18.55253 | 19.33100 | 15.26820 | 13.20284 | 16.21234 | 8.78971 | 13.69573 | 7.64108 | 12.51527 | 6.82131 | 6.53398 | 4.60262 | 7.93785 | 3.65981 | 1.98451 | 6.14091 |
| **3** | 195.00633 | 118.95157 | 127.97550 | 134.46867 | 108.87887 | 109.96967 | 95.75977 | 71.40153 | 75.61567 | 61.11083 | 43.05697 | 58.53747 | 57.60723 | 50.32600 | 59.16923 | 53.16897 | 42.30223 | 57.43597 |
| **4** | 28.11723 | 39.01830 | 31.91323 | 32.69387 | 25.37967 | 21.03430 | 21.53017 | 16.62490 | 17.72703 | 11.24997 | 13.47310 | 13.22962 | 11.85990 | 10.62650 | 13.36856 | 9.38019 | 8.26683 | 12.93002 |
| **5** | 32.28777 | 31.34900 | 31.09887 | 29.87790 | 24.92060 | 23.76037 | 25.38940 | 21.40227 | 21.49113 | 16.52983 | 17.05223 | 16.27130 | 16.90133 | 13.77700 | 16.25793 | 12.73247 | 10.52070 | 11.88530 |
| **6** | 45.26547 | 33.17020 | 41.54980 | 41.43363 | 37.73330 | 41.03237 | 38.06627 | 34.12847 | 32.49520 | 32.36837 | 30.77237 | 30.21490 | 25.54900 | 24.70877 | 26.86717 | 24.21303 | 21.32253 | 24.45497 |
| **7** | 78.34833 | 78.02100 | 72.96610 | 72.50737 | 68.47677 | 62.59680 | 62.72183 | 59.34610 | 54.58330 | 56.66083 | 53.79513 | 54.44573 | 52.55070 | 44.23117 | 48.01957 | 43.44693 | 33.72813 | 35.28943 |
| **8** | 141.83500 | 96.29267 | 149.87400 | 91.10700 | 123.19933 | 63.61473 | 112.39133 | 61.86570 | 64.41557 | 65.89823 | 64.00043 | 91.24720 | 98.53147 | 83.51093 | 85.24603 | 54.62330 | 54.32763 | 82.12307 |
| **9** | 42.85033 | 41.90950 | 43.63147 | 43.85960 | 41.12070 | 40.40997 | 34.79733 | 36.75147 | 35.33610 | 35.26233 | 38.54703 | 36.12203 | 35.15570 | 34.08253 | 34.82650 | 31.78973 | 31.66830 | 32.72210 |
| **10** | 15.50527 | 15.63373 | 14.57563 | 15.47100 | 12.33397 | 13.58477 | 12.10770 | 11.93410 | 11.57669 | 12.29723 | 11.31023 | 10.95297 | 10.01382 | 9.97069 | 10.78978 | 8.78829 | 7.40777 | 8.22316 |
| **11** | 466.73633 | 494.30100 | 450.93500 | 521.48533 | 547.91633 | 527.27367 | 517.05067 | 538.19367 | 537.87933 | 496.68767 | 526.52800 | 490.67133 | 494.68267 | 531.83733 | 517.52733 | 485.22300 | 458.43433 | 501.96167 |
| **12** | 24.23630 | 20.22113 | 21.67110 | 21.91147 | 20.43247 | 17.04447 | 16.92407 | 16.91527 | 16.72260 | 11.94300 | 16.33897 | 17.13833 | 15.82660 | 9.99333 | 13.72610 | 13.36293 | 8.04837 | 5.36580 |
| **13** | 57.17620 | 55.83250 | 62.57017 | 62.79230 | 52.31640 | 54.28400 | 53.16423 | 46.96923 | 46.20240 | 47.19227 | 52.10923 | 46.53603 | 46.01293 | 46.23143 | 49.04483 | 42.68283 | 42.74800 | 50.02407 |
| **14** | 42.01317 | 45.27813 | 42.88693 | 45.49150 | 39.55007 | 33.49987 | 35.44890 | 36.13490 | 35.30083 | 32.21760 | 34.15677 | 35.89910 | 35.09667 | 31.58680 | 33.30270 | 31.86953 | 30.01300 | 31.30670 |
| **15** | 55.14533 | 58.41807 | 54.74247 | 53.52070 | 54.09880 | 47.64987 | 47.61813 | 49.38397 | 45.88990 | 47.89910 | 47.46937 | 47.49093 | 47.10467 | 46.62593 | 50.04647 | 47.48243 | 50.75363 | 49.27047 |
| **16** | 27.84413 | 29.87440 | 26.56327 | 29.21873 | 25.36217 | 24.46817 | 24.35607 | 22.54210 | 23.43030 | 23.23300 | 24.54250 | 23.98677 | 24.05503 | 24.92693 | 27.16017 | 27.14817 | 25.23583 | 24.97807 |
| **17** | 48.30113 | 47.98193 | 48.89857 | 44.68747 | 41.87197 | 42.04637 | 40.78407 | 39.06377 | 39.55070 | 34.94120 | 39.28443 | 36.02467 | 37.31040 | 38.77220 | 37.92177 | 38.04687 | 35.64183 | 39.25713 |
| **18** | 17.84513 | 64.54093 | 51.54930 | 48.43613 | 35.85980 | 26.38953 | 32.93990 | 24.19283 | 29.24210 | 17.55473 | 23.11507 | 21.34997 | 19.27937 | 17.27213 | 22.63743 | 18.86140 | 16.88280 | 21.91323 |
| **19** | 52.44097 | 46.30150 | 58.12463 | 55.27307 | 49.93760 | 48.30880 | 48.81303 | 40.23500 | 46.97710 | 48.75273 | 50.36373 | 52.03333 | 50.77500 | 47.91140 | 47.95890 | 47.16470 | 48.96863 | 47.51993 |
| **20** | 84.96280 | 79.11370 | 88.50567 | 89.73007 | 77.68233 | 69.86240 | 65.05417 | 67.37040 | 69.51860 | 69.31940 | 74.36193 | 72.57523 | 65.46090 | 64.44177 | 73.72500 | 72.58653 | 63.06197 | 70.63860 |
| **21** | 11.79817 | 12.40737 | 12.20833 | 11.03822 | 9.56356 | 9.43376 | 8.99276 | 8.64934 | 8.44251 | 8.63619 | 9.79057 | 8.81140 | 9.28673 | 9.17184 | 8.27048 | 9.31503 | 11.13220 | 9.20312 |
| **22** | 16.94593 | 25.26057 | 20.84330 | 21.76703 | 14.63425 | 9.68146 | 9.01293 | 5.93854 | 6.31077 | 4.00496 | 5.30303 | 5.88411 | 4.24035 | 4.08609 | 5.58707 | 3.44359 | 2.12070 | 5.06398 |
| **23** | 17.72693 | 16.20707 | 16.98950 | 18.20633 | 15.11180 | 12.44690 | 12.51527 | 13.31400 | 14.10480 | 13.76740 | 11.54403 | 13.48463 | 12.10083 | 12.89400 | 13.36220 | 14.26613 | 15.07797 | 14.02813 |
| **24** | 110.71393 | 119.10433 | 110.57333 | 124.62300 | 113.46233 | 120.31100 | 121.64433 | 114.85967 | 113.29467 | 108.69053 | 120.57967 | 112.21933 | 119.42833 | 129.29433 | 118.54267 | 112.79233 | 116.29900 | 113.24500 |
| **25** | 144.67200 | 116.29880 | 127.43933 | 122.32967 | 96.12510 | 91.56290 | 83.89860 | 105.33670 | 90.08820 | 94.32283 | 86.65167 | 87.41757 | 92.85987 | 92.30120 | 89.60097 | 90.62297 | 89.58740 | 91.71687 |
| **26** | 30.68460 | 23.56523 | 29.28157 | 27.76277 | 28.54587 | 31.42900 | 29.64817 | 28.63080 | 30.56323 | 30.75843 | 33.63270 | 32.16437 | 30.02480 | 31.58963 | 34.05347 | 33.31333 | 36.41380 | 34.41973 |
| **27** | 10.85283 | 11.49426 | 19.02390 | 22.55230 | 18.58836 | 22.83217 | 20.07800 | 11.90443 | 14.80643 | 6.75420 | 8.35548 | 4.39246 | 2.01173 | 0.84175 | 3.50520 | 0.28532 | 0.24921 | 3.63715 |
| **28** | 15.71027 | 15.19170 | 16.39087 | 16.21810 | 15.65983 | 13.23697 | 12.45757 | 13.90547 | 11.93513 | 11.94273 | 13.24210 | 13.22350 | 12.72057 | 14.69287 | 14.33460 | 15.66930 | 14.51233 | 13.84920 |
| **29** | 69.73027 | 72.78583 | 85.08337 | 77.99747 | 67.20330 | 57.20593 | 63.43317 | 57.44020 | 61.85130 | 71.24593 | 66.33490 | 47.01660 | 61.97123 | 63.95577 | 67.62090 | 78.63700 | 76.42870 | 71.96727 |
| **30** | 93.25953 | 92.98737 | 90.14193 | 91.49260 | 93.77183 | 102.01500 | 103.94690 | 110.97300 | 111.29470 | 98.97090 | 116.54467 | 97.86613 | 117.12333 | 108.37600 | 97.10993 | 102.18197 | 93.43957 | 118.53833 |
| **31** | 109.10887 | 133.79133 | 127.38767 | 113.36130 | 136.22233 | 120.69067 | 134.52833 | 140.94900 | 134.81300 | 114.32703 | 125.68467 | 140.78633 | 136.69533 | 97.91500 | 104.27167 | 143.75833 | 156.00033 | 160.60800 |
| **32** | 30.63860 | 31.35837 | 27.01850 | 26.21457 | 29.19807 | 31.26623 | 31.39757 | 32.73750 | 32.40293 | 26.98453 | 30.16587 | 30.86763 | 33.64733 | 28.55513 | 27.28210 | 26.02500 | 23.49630 | 29.27543 |
| **33** | 231.01300 | 246.49400 | 239.74333 | 223.23933 | 232.68133 | 219.50000 | 228.63500 | 221.59900 | 226.34733 | 230.62633 | 204.17300 | 223.45267 | 217.64167 | 215.95933 | 219.63133 | 216.47267 | 210.61533 | 226.56367 |
| **34** | 42.15440 | 37.99707 | 39.44377 | 33.36927 | 35.79140 | 36.13570 | 32.87337 | 38.88673 | 24.28690 | 35.67240 | 23.58200 | 32.64977 | 21.91673 | 21.13077 | 23.40213 | 12.99000 | 15.43227 | 20.04490 |
| **35** | 6.74177 | 8.65842 | 5.82188 | 9.30318 | 8.65491 | 7.67202 | 5.41706 | 5.16618 | 8.41630 | 6.10455 | 9.43424 | 7.31674 | 9.32228 | 10.36804 | 9.81050 | 10.38973 | 10.12969 | 10.04722 |
| **36** | 73.42903 | 56.32137 | 64.51397 | 64.51287 | 57.50063 | 50.59453 | 46.19513 | 57.94357 | 36.24307 | 55.69113 | 26.96687 | 46.78520 | 21.45000 | 26.68269 | 28.99610 | 8.37880 | 15.26456 | 13.77123 |
| **37** | 21.62397 | 14.16830 | 16.24990 | 18.04790 | 18.47510 | 18.36180 | 17.90723 | 16.99867 | 16.77847 | 21.37463 | 21.84473 | 21.13197 | 20.22897 | 24.10620 | 20.91730 | 22.59387 | 27.07393 | 24.66490 |
| **38** | 217.92333 | 228.12167 | 218.39200 | 220.06967 | 210.05967 | 204.09233 | 225.49367 | 214.51433 | 213.06133 | 202.29533 | 211.20200 | 200.63167 | 205.71133 | 210.80067 | 211.03500 | 208.42900 | 203.91833 | 208.03267 |
| **39** | 14.85357 | 13.78490 | 15.29433 | 15.38573 | 13.68840 | 14.68217 | 13.86553 | 13.58600 | 14.13513 | 13.86730 | 15.63763 | 15.34090 | 12.86590 | 14.45357 | 14.60910 | 14.04720 | 16.84160 | 15.97880 |
| **40** | 47.04637 | 71.76387 | 54.06100 | 66.43693 | 54.62260 | 53.26890 | 58.33587 | 54.91993 | 70.41110 | 54.60303 | 80.07720 | 60.19117 | 77.95553 | 82.36357 | 77.59233 | 110.38003 | 98.82503 | 113.28740 |
| **41** | 19.97723 | 19.16413 | 19.72540 | 19.40407 | 14.30870 | 12.58807 | 12.32687 | 10.99408 | 10.92301 | 10.68507 | 9.20170 | 10.71827 | 10.60133 | 11.37766 | 10.95864 | 12.17653 | 11.49717 | 11.89540 |
| **42** | 23.85540 | 7.88419 | 15.53343 | 11.36337 | 13.58640 | 14.57766 | 10.46541 | 19.32230 | 9.30146 | 22.50097 | 11.71857 | 22.16093 | 16.66070 | 21.84877 | 19.81933 | 22.40393 | 26.09137 | 21.02217 |
| **43** | 103.90800 | 70.63067 | 65.21100 | 125.36933 | 199.51433 | 166.15233 | 59.76267 | 87.68787 | 136.82267 | 197.06700 | 189.21700 | 182.95767 | 213.24533 | 142.72233 | 84.49620 | 189.97333 | 141.34633 | 74.82333 |
| **44** | 4.75416 | 0.00000 | 8.70370 | 5.93465 | 7.03730 | 6.85967 | 9.75888 | 8.19848 | 9.93786 | 8.31543 | 11.76903 | 9.56414 | 9.79421 | 11.83460 | 10.07983 | 10.82160 | 13.09423 | 7.59405 |
| **45** | 19.38263 | 19.90107 | 20.10850 | 19.20477 | 17.08743 | 14.89170 | 14.98063 | 13.46807 | 13.81157 | 14.83543 | 16.44887 | 15.23757 | 14.57530 | 14.28597 | 15.20157 | 13.25043 | 13.19890 | 12.85373 |
| **46** | 24.72343 | 18.01070 | 22.05410 | 18.19360 | 18.41660 | 18.66537 | 16.97140 | 18.38960 | 11.41127 | 17.96173 | 9.46385 | 14.79497 | 7.98893 | 7.80205 | 9.12300 | 2.66699 | 4.56961 | 8.55692 |
| **47** | 17.65013 | 17.39370 | 19.33620 | 18.50520 | 18.16360 | 17.81867 | 20.31737 | 18.63893 | 19.12390 | 17.11273 | 19.56600 | 19.04087 | 18.16170 | 19.56903 | 17.92230 | 17.86940 | 17.00093 | 16.88283 |
| **48** | 16.39740 | 20.48767 | 19.34807 | 22.46237 | 20.69797 | 17.50493 | 19.11363 | 18.99913 | 20.19787 | 17.96807 | 22.91757 | 19.90007 | 21.90963 | 23.04080 | 20.85530 | 26.35607 | 27.00043 | 26.81367 |
| **49** | 23.47690 | 24.81403 | 24.00163 | 23.82003 | 23.54350 | 26.12030 | 24.66480 | 23.54647 | 24.97530 | 22.93080 | 24.23530 | 24.68540 | 26.67493 | 25.59140 | 25.98337 | 24.66237 | 27.46160 | 26.80767 |
| **50** | 102.83770 | 77.74053 | 75.80167 | 76.53740 | 79.38393 | 81.42243 | 78.50947 | 83.37550 | 74.90573 | 77.07627 | 75.73183 | 73.72447 | 74.47797 | 69.33857 | 65.04937 | 53.22353 | 41.65293 | 56.13413 |
| **51** | 67.19070 | 49.94647 | 36.56270 | 37.38700 | 52.37360 | 53.61547 | 63.50893 | 47.77947 | 44.25650 | 33.77773 | 34.50620 | 44.00797 | 30.67567 | 40.61573 | 42.57057 | 32.53490 | 23.98227 | 35.09690 |
| **52** | 19.44610 | 18.82670 | 19.89157 | 20.94127 | 17.88083 | 20.22460 | 19.08200 | 17.26473 | 19.60343 | 17.97833 | 21.02150 | 19.79100 | 17.99417 | 19.43183 | 21.80587 | 20.25863 | 22.61740 | 21.95443 |
| **53** | 28.47490 | 12.32487 | 21.12660 | 15.70397 | 13.92983 | 17.31915 | 12.22099 | 18.29563 | 8.04249 | 18.86887 | 6.61443 | 12.29760 | 5.29653 | 2.49981 | 5.16062 | 0.99039 | 0.23138 | 4.21327 |
| **54** | 17.06060 | 13.35243 | 18.79210 | 16.24543 | 14.72227 | 14.33960 | 13.80450 | 13.44427 | 13.88417 | 16.51763 | 15.99870 | 15.21163 | 14.76050 | 17.22263 | 16.68257 | 17.53237 | 20.73323 | 18.52733 |
| **55** | 39.45177 | 33.95927 | 43.43917 | 41.52290 | 42.24180 | 48.19503 | 45.34717 | 44.37040 | 41.91220 | 49.38907 | 44.63753 | 45.31817 | 41.76177 | 35.76327 | 33.05397 | 21.59870 | 20.74033 | 27.79173 |
| **56** | 82.71453 | 88.23017 | 101.90170 | 126.93367 | 104.19500 | 105.09067 | 121.47700 | 105.92630 | 115.66633 | 98.11837 | 97.95027 | 115.84057 | 108.79467 | 132.73600 | 114.55723 | 136.03300 | 123.19867 | 134.62477 |
| **57** | 17.47670 | 10.69561 | 13.91248 | 13.45890 | 13.82063 | 13.55857 | 12.71173 | 16.34173 | 13.87743 | 19.19893 | 15.44243 | 18.69847 | 18.46800 | 21.50337 | 16.59020 | 19.92237 | 18.68790 | 14.59560 |
| **58** | 122.86567 | 121.64233 | 129.08667 | 128.36433 | 123.84867 | 122.75667 | 122.57833 | 128.80067 | 132.14367 | 122.24533 | 127.92633 | 132.82633 | 143.61467 | 144.33367 | 155.35400 | 136.34967 | 158.80433 | 149.35967 |
| **59** | 43.54837 | 47.34390 | 30.46293 | 40.31437 | 63.98980 | 74.03143 | 77.53103 | 64.43460 | 66.05647 | 53.29210 | 56.32077 | 64.09323 | 47.71010 | 53.66057 | 58.84903 | 39.52323 | 22.23700 | 40.07977 |
| **60** | 77.17003 | 78.74553 | 88.21093 | 82.71380 | 73.81270 | 67.80350 | 69.91727 | 64.60937 | 74.36093 | 67.95627 | 70.68603 | 71.51007 | 62.14613 | 75.77257 | 77.53197 | 82.47537 | 87.32897 | 81.89317 |
| **61** | 21.33487 | 13.87050 | 19.78047 | 18.96297 | 18.07937 | 15.58733 | 15.47550 | 18.31080 | 10.43863 | 19.62073 | 18.95457 | 19.92267 | 20.72890 | 22.20467 | 18.52057 | 23.81010 | 24.24100 | 22.41400 |
| **62** | 167.54967 | 141.54067 | 168.06367 | 183.57433 | 174.16000 | 192.27033 | 176.38667 | 193.20000 | 184.78833 | 174.08933 | 201.00867 | 203.90133 | 193.72933 | 205.01733 | 197.60267 | 202.79700 | 230.61433 | 208.49267 |
| **63** | 57.88957 | 39.13230 | 47.68317 | 43.73887 | 42.30403 | 49.16903 | 46.92877 | 57.56370 | 40.36483 | 51.58593 | 40.11657 | 42.17427 | 35.68710 | 21.23750 | 23.13340 | 7.98865 | 4.74196 | 18.85932 |
| **64** | 26.38413 | 23.35507 | 23.52400 | 25.03317 | 22.47240 | 20.60217 | 21.23950 | 19.36147 | 18.30607 | 19.00283 | 22.94120 | 21.26627 | 20.71853 | 19.77253 | 19.15480 | 15.34580 | 14.48617 | 15.51133 |
| **65** | 16.21833 | 29.04200 | 29.51073 | 32.81450 | 29.94567 | 30.86137 | 28.00160 | 31.97790 | 31.28947 | 30.71227 | 33.48837 | 34.46700 | 33.00670 | 34.19197 | 31.75730 | 36.94200 | 35.16783 | 34.49753 |
| **66** | 14.16307 | 12.81380 | 13.83873 | 14.84183 | 12.38603 | 11.13183 | 11.18328 | 13.04777 | 12.50893 | 10.83553 | 10.76030 | 11.91697 | 12.33727 | 13.63167 | 11.33123 | 13.77990 | 13.46817 | 12.25837 |
| **67** | 18.13880 | 18.48897 | 16.25770 | 18.71930 | 18.23557 | 17.84430 | 17.45840 | 15.37890 | 16.34100 | 15.87750 | 18.06723 | 16.60903 | 14.39960 | 17.24980 | 18.53740 | 17.80453 | 20.28697 | 18.74210 |
| **68** | 49.45933 | 32.39317 | 41.96503 | 32.31157 | 33.06873 | 31.81603 | 27.72080 | 35.26813 | 24.81323 | 36.60123 | 23.23937 | 32.66987 | 26.90380 | 31.20723 | 28.92547 | 27.26000 | 29.16510 | 23.78417 |
| **69** | 67.07690 | 88.33133 | 70.91693 | 75.95400 | 78.56460 | 80.31973 | 78.48347 | 70.34003 | 81.06713 | 69.51203 | 84.17213 | 79.68203 | 71.20433 | 80.95737 | 85.97517 | 81.02083 | 94.29293 | 91.45193 |
| **70** | 78.92963 | 72.02583 | 91.79330 | 92.30720 | 100.38170 | 106.08080 | 112.56190 | 109.14043 | 109.79600 | 111.63600 | 104.34770 | 107.18297 | 117.63833 | 119.73467 | 131.01033 | 123.87400 | 135.82333 | 132.44067 |
| **71** | 48.84073 | 56.19090 | 66.93557 | 56.47050 | 57.83670 | 53.07473 | 55.12663 | 43.44197 | 53.48380 | 45.19320 | 49.46473 | 48.39913 | 46.82343 | 49.32143 | 50.70220 | 49.21570 | 53.72610 | 56.26653 |
| **72** | 96.95493 | 118.64467 | 115.39067 | 116.47820 | 118.46633 | 126.00567 | 133.23300 | 133.01600 | 133.42700 | 116.08500 | 135.30867 | 125.12167 | 129.56433 | 129.26533 | 127.89033 | 145.28933 | 122.45667 | 130.24033 |
| **73** | 42.42967 | 39.89140 | 43.60360 | 41.36490 | 43.98830 | 46.18790 | 47.05770 | 48.64813 | 46.12703 | 53.47297 | 47.74273 | 48.65850 | 49.96480 | 51.40557 | 51.80900 | 52.26723 | 52.46677 | 58.21040 |
| **74** | 201.62133 | 238.64333 | 226.91133 | 246.57133 | 233.86433 | 249.53967 | 243.06067 | 239.92533 | 270.38167 | 235.93967 | 237.86600 | 228.63433 | 257.53133 | 251.04667 | 239.85167 | 276.97567 | 266.26067 | 266.63833 |
| **75** | 29.84420 | 28.57990 | 31.68963 | 31.21530 | 30.54370 | 31.78213 | 28.93177 | 26.77700 | 29.46303 | 27.24990 | 28.56067 | 29.78907 | 25.20687 | 31.65160 | 32.65080 | 31.12537 | 36.21193 | 35.04603 |
| **76** | 147.32833 | 160.95700 | 149.93833 | 101.88433 | 162.28867 | 166.12100 | 157.95900 | 174.71300 | 167.40900 | 164.74933 | 153.82667 | 154.78267 | 168.39433 | 169.31067 | 174.47733 | 173.35267 | 171.82767 | 168.87300 |
| **77** | 122.04967 | 121.57100 | 137.01733 | 136.22867 | 127.37600 | 144.44933 | 158.26167 | 156.37100 | 157.88000 | 142.93933 | 144.97633 | 147.25000 | 161.15367 | 142.29300 | 136.46633 | 144.14833 | 161.79767 | 168.38100 |
| **78** | 22.79413 | 27.23093 | 29.05093 | 30.88903 | 29.24070 | 29.61750 | 28.14843 | 28.28697 | 32.77637 | 33.32420 | 32.42880 | 31.78330 | 28.32947 | 34.31977 | 36.52557 | 32.84787 | 38.93900 | 33.09997 |
| **79** | 71.01563 | 68.27303 | 81.71050 | 85.84993 | 77.19670 | 79.97373 | 91.93133 | 84.78610 | 94.52530 | 86.98910 | 93.81350 | 91.75390 | 90.99340 | 102.73720 | 109.71067 | 100.01287 | 122.56600 | 99.60573 |
| **80** | 46.47303 | 42.64550 | 44.54347 | 43.27693 | 42.75197 | 45.94223 | 47.32000 | 52.82563 | 46.88593 | 50.87637 | 49.31443 | 50.70337 | 47.83513 | 52.48187 | 48.70550 | 50.00783 | 47.36720 | 44.03607 |
| **81** | 43.85637 | 31.71547 | 40.47157 | 35.12073 | 43.13787 | 44.79057 | 40.30993 | 49.48927 | 36.16810 | 53.15243 | 37.05343 | 50.71633 | 45.39113 | 50.93223 | 45.93980 | 48.95910 | 52.13987 | 47.98227 |
| **82** | 184.55133 | 211.37267 | 220.56733 | 206.72500 | 213.70867 | 220.80567 | 247.61833 | 248.10233 | 227.28667 | 231.70633 | 240.81467 | 233.26167 | 246.74933 | 240.72367 | 218.76133 | 242.65500 | 252.13967 | 253.03400 |
| **83** | 62.88013 | 50.96803 | 60.13557 | 53.89620 | 54.94420 | 70.95457 | 73.76353 | 86.73167 | 67.13350 | 90.93157 | 57.84677 | 76.53910 | 77.07920 | 81.18660 | 73.04777 | 74.24603 | 77.67390 | 65.93727 |
| **84** | 23.33903 | 23.45743 | 30.43367 | 28.01837 | 26.28073 | 30.04940 | 27.08710 | 27.11493 | 30.13140 | 28.73473 | 30.52527 | 29.08397 | 26.77293 | 31.20127 | 32.12517 | 33.50250 | 36.50490 | 34.18570 |
| **85** | 49.37113 | 58.68583 | 51.32223 | 54.16400 | 54.88087 | 66.87683 | 65.38477 | 68.32477 | 72.39030 | 62.93077 | 72.06527 | 71.72237 | 65.65500 | 74.63850 | 64.06260 | 72.44393 | 74.94953 | 71.01453 |
| **86** | 74.29650 | 58.13917 | 66.71943 | 58.46120 | 77.32243 | 72.69143 | 81.10667 | 100.49097 | 75.04477 | 97.68217 | 76.65737 | 96.50063 | 75.73020 | 83.63907 | 88.51733 | 77.69323 | 76.70863 | 75.64440 |
| **87** | 6.47959 | 10.75931 | 11.70576 | 12.50357 | 12.80610 | 14.68967 | 15.27073 | 13.68313 | 16.10890 | 16.07770 | 16.43973 | 15.22923 | 16.07170 | 17.99533 | 18.55500 | 16.71270 | 21.02967 | 20.10503 |
| **88** | 6.26739 | 10.60109 | 9.11745 | 11.78530 | 11.33293 | 11.25627 | 11.79377 | 10.79313 | 13.82530 | 11.20017 | 11.57570 | 13.61243 | 15.19223 | 17.31783 | 15.22303 | 17.70983 | 17.66743 | 16.34863 |
| **89** | 67.84000 | 57.20507 | 59.65690 | 53.30573 | 58.76493 | 66.46440 | 64.22877 | 76.68200 | 65.05793 | 84.27067 | 68.40810 | 78.97450 | 73.13023 | 73.27963 | 70.77090 | 69.81667 | 74.00580 | 70.85697 |
| **90** | 26.43770 | 34.08323 | 34.60610 | 35.91543 | 35.88397 | 37.31817 | 33.11863 | 33.16460 | 31.40633 | 32.16813 | 32.30280 | 30.40003 | 31.76697 | 37.05877 | 35.75373 | 35.89183 | 37.41557 | 39.39383 |
| **91** | 9.39131 | 20.53047 | 21.64517 | 21.62103 | 26.11897 | 28.35630 | 28.10313 | 31.74300 | 35.00057 | 36.95173 | 35.06720 | 34.89397 | 37.77750 | 46.57293 | 41.61733 | 46.58723 | 51.80147 | 43.79047 |
| **92** | 32.15403 | 39.64147 | 39.28460 | 42.64203 | 37.05910 | 35.20237 | 34.97510 | 30.52947 | 36.02350 | 30.72033 | 33.42003 | 31.39193 | 33.67470 | 32.05473 | 32.64543 | 34.23763 | 33.98480 | 34.67613 |
| **93** | 23.31370 | 24.29420 | 27.37413 | 23.94937 | 17.19260 | 11.44627 | 13.63190 | 15.10347 | 17.12147 | 19.74147 | 18.59773 | 18.07967 | 19.58853 | 18.13543 | 17.84540 | 17.95473 | 19.12093 | 15.42453 |
| **94** | 5.18570 | 12.31480 | 11.92532 | 12.81723 | 16.24747 | 17.01537 | 18.69400 | 16.96837 | 20.90297 | 17.77973 | 20.38990 | 18.92233 | 18.66553 | 21.60070 | 23.55507 | 22.95403 | 26.88753 | 23.86080 |
| **95** | 144.58733 | 156.80200 | 142.76400 | 137.60633 | 140.61100 | 150.71933 | 141.79467 | 154.73133 | 143.42467 | 125.49733 | 123.35800 | 136.58633 | 156.32433 | 130.07267 | 138.21467 | 128.37500 | 116.03933 | 140.69400 |
| **96** | 240.43900 | 313.55367 | 330.15100 | 344.40600 | 354.82433 | 371.87833 | 378.62533 | 405.33933 | 411.00400 | 382.86300 | 351.07533 | 408.54133 | 364.96800 | 362.30700 | 380.39833 | 361.91467 | 401.60500 | 411.85667 |
| **97** | 54.18387 | 55.82950 | 51.24683 | 52.86350 | 53.71510 | 56.60070 | 56.62053 | 55.67270 | 54.34750 | 50.43377 | 52.83753 | 55.91293 | 56.36547 | 55.45743 | 52.81263 | 54.87193 | 52.03883 | 52.75580 |
| **98** | 32.63117 | 36.92317 | 28.61357 | 31.93460 | 34.40233 | 41.74470 | 38.47123 | 34.11623 | 38.66937 | 37.15580 | 37.68150 | 36.00900 | 34.64260 | 33.18053 | 38.78077 | 33.25107 | 35.76640 | 35.63193 |
| **99** | 19.98140 | 21.83040 | 17.69130 | 19.25487 | 19.59447 | 21.77807 | 20.11463 | 20.47913 | 20.32400 | 20.89150 | 20.98417 | 22.84780 | 22.58267 | 20.70837 | 21.81727 | 22.95030 | 23.77783 | 22.28480 |
| **100** | 110.86400 | 141.03867 | 132.63900 | 128.39300 | 130.47833 | 135.54667 | 127.16500 | 163.07100 | 144.20933 | 155.90500 | 161.99567 | 166.26733 | 164.93733 | 164.68733 | 153.25333 | 157.62600 | 161.03167 | 160.83600 |
| **101** | 0.00000 | 4.43937 | 0.00000 | 0.00000 | 9.34983 | 14.83703 | 17.17057 | 13.35496 | 16.34117 | 16.71300 | 18.30670 | 16.77607 | 17.11037 | 19.45280 | 16.72573 | 18.49250 | 17.86783 | 13.43970 |
| **102** | 0.00000 | 3.91350 | 0.00000 | 0.00000 | 6.67461 | 7.18614 | 13.01905 | 24.15523 | 28.44083 | 27.18140 | 27.09427 | 25.55267 | 29.17363 | 30.05300 | 22.24587 | 33.53223 | 23.97550 | 22.35717 |
| **103** | 0.00000 | 6.23333 | 0.00000 | 0.00000 | 12.74297 | 17.80857 | 20.79923 | 22.50540 | 29.64630 | 34.04563 | 33.70453 | 28.35470 | 32.29513 | 32.41263 | 30.02673 | 30.64127 | 25.00263 | 17.49753 |
| **104** | 0.00000 | 2.08736 | 0.00000 | 0.00000 | 5.03715 | 6.71990 | 9.50803 | 10.57230 | 14.15860 | 13.98280 | 14.93950 | 12.98430 | 14.02757 | 15.49047 | 13.83799 | 15.84617 | 15.39210 | 10.45291 |
| **105** | 0.00000 | 0.00000 | 1.50745 | 1.41131 | 4.93454 | 21.31503 | 36.66183 | 31.10047 | 20.69743 | 24.20023 | 20.76953 | 26.28353 | 19.78880 | 23.23340 | 20.18393 | 25.81817 | 26.07800 | 24.66680 |
| **106** | 0.00000 | 20.88060 | 0.00000 | 0.00000 | 36.22430 | 49.98187 | 70.52487 | 88.87363 | 110.19350 | 126.33200 | 82.10887 | 107.33280 | 119.34667 | 117.29867 | 95.63013 | 99.98830 | 92.95940 | 66.72210 |
| **107** | 0.00000 | 4.17040 | 0.00000 | 0.00000 | 7.90548 | 8.37153 | 16.08764 | 25.61360 | 30.12060 | 38.26787 | 35.80953 | 32.83363 | 37.29137 | 38.28220 | 32.88367 | 34.62723 | 27.62840 | 17.95980 |
| **108** | 0.00000 | 0.00000 | 0.00000 | 0.00000 | 1.37201 | 4.14124 | 13.80196 | 16.05193 | 24.93923 | 26.67373 | 28.01533 | 16.98593 | 26.58050 | 25.26827 | 17.05510 | 18.86462 | 21.77813 | 10.13073 |

**Table S4**

The scoring coefficient matrix of E1 (10 indicators, KMO = 0.548 & significance of Bartlett’s Test = 0, initial eigenvalue > 0.8, on behalf of 80.834% raw data).

| Variables | F1 | F2 | F3 | F4 | F5 | F6 |
| --- | --- | --- | --- | --- | --- | --- |
| First black stool time /min | -0.264 | -0.212 | 0.099 | 0.247 | -0.181 | -0.141 |
| No. of black stools | 0.453 | -0.098 | 0.074 | -0.036 | -0.170 | -0.032 |
| Fecal weights within 12 h /g | 0.472 | -0.115 | -0.027 | 0.234 | -0.002 | 0.000 |
| Colonic content weights /g | -0.073 | -0.050 | 0.046 | -0.045 | 0.892 | -0.016 |
| Organ coefficient of colon /% | 0.062 | -0.004 | 0.001 | 0.770 | -0.019 | 0.076 |
| Organ coefficient of stomach /% | 0.071 | 0.058 | 0.040 | 0.060 | -0.028 | 0.939 |
| MTL /pg·mL^-1^ | -0.072 | 0.663 | -0.230 | -0.002 | 0.166 | -0.095 |
| SS /pg·mL^-1^ | -0.048 | 0.561 | 0.078 | -0.031 | -0.241 | 0.162 |
| VIP /pg·mL^-1^ | 0.055 | -0.067 | 0.569 | 0.361 | -0.076 | -0.154 |
| AchE /nmol·L^-1^ | -0.070 | -0.137 | 0.691 | -0.223 | 0.109 | 0.114 |
| Total weight of common factors | 2.088 | 1.382 | 1.280 | 1.180 | 1.089 | 1.064 |

**Table S5**

The scoring coefficient matrix of E2 (13 indicators, KMO = 0.562 & significance of Bartlett’s Test = 0, initial eigenvalue > 0.7, on behalf of 82.112% raw data).

| Variables | F1 | F2 | F3 | F4 | F5 | F6 | F7 |
| --- | --- | --- | --- | --- | --- | --- | --- |
| TG /nmol·L^-1^ | -0.002 | -0.170 | -0.111 | -0.199 | 0.786 | -0.043 | 0.086 |
| Na^+^-K^+^-ATPase /μmol·mL^-1^ | -0.030 | 0.479 | 0.303 | -0.282 | -0.031 | 0.019 | -0.036 |
| TNF-α /pg·mL^-1^ | 0.033 | 0.651 | -0.217 | 0.126 | -0.236 | -0.063 | 0.137 |
| IL-1β /pg·mL^-1^ | 0.126 | -0.034 | -0.123 | -0.188 | -0.198 | 0.569 | 0.150 |
| Ratio (p-NF-κB p65/GAPDH) | -0.339 | -0.125 | 0.086 | 0.326 | 0.223 | 0.172 | -0.009 |
| Ratio (NF-κB p65/GAPDH) | 0.058 | 0.014 | -0.160 | 0.732 | -0.219 | -0.103 | -0.014 |
| Ratio (p-p38/GAPDH) | -0.168 | 0.068 | 0.521 | 0.088 | -0.063 | 0.000 | 0.015 |
| Ratio (p38/GAPDH) | -0.096 | 0.081 | 0.052 | -0.131 | 0.004 | -0.076 | 0.954 |
| Ratio (p-ERK/GAPDH) | 0.219 | -0.083 | 0.090 | 0.079 | 0.288 | 0.070 | -0.152 |
| Ratio (ERK/GAPDH) | 0.359 | 0.132 | -0.044 | 0.036 | 0.019 | -0.142 | 0.008 |
| Ratio (p-JNK/GAPDH) | 0.040 | 0.030 | 0.088 | 0.018 | 0.061 | 0.574 | -0.151 |
| Ratio (JNK/GAPDH) | 0.413 | -0.137 | -0.111 | 0.079 | 0.011 | 0.242 | -0.024 |
| Ratio (TLR4/GAPDH) | 0.088 | -0.128 | 0.528 | -0.242 | -0.153 | -0.032 | 0.143 |
| Total weight of common factors | 2.172 | 1.628 | 1.567 | 1.430 | 1.422 | 1.363 | 1.092 |

**Table S6**

The scoring coefficient matrix of E3 (16 indicators, KMO = 0.646 & significance of Bartlett’s Test = 0, initial eigenvalue > 0.7, on behalf of 78.44% raw data).

| Variables | F1 | F2 | F3 | F4 | F5 | F6 | F7 | F8 |
| --- | --- | --- | --- | --- | --- | --- | --- | --- |
| Anal temperatures /°C | 0.109 | -0.127 | 0.043 | 0.063 | 0.007 | -0.002 | -0.011 | 0.879 |
| HSP-70 /pg·mL^-1^ | 0.095 | 0.075 | 0.138 | -0.007 | -0.675 | 0.025 | 0.077 | -0.026 |
| SOD /ng·mL^-1^ | 0.430 | -0.434 | -0.122 | 0.169 | -0.130 | 0.086 | 0.013 | 0.130 |
| NO /μmol·L^-1^ | -0.149 | -0.099 | 0.070 | 0.489 | 0.174 | 0.058 | 0.233 | -0.007 |
| TNF-α /pg·mL^-1^ | -0.176 | -0.015 | -0.099 | -0.050 | -0.119 | 0.705 | 0.029 | -0.079 |
| IL-1β /pg·mL^-1^ | -0.105 | 0.048 | -0.020 | 0.009 | -0.069 | -0.016 | 0.645 | -0.036 |
| IL-6 /pg·mL^-1^ | -0.034 | 0.288 | 0.004 | 0.625 | -0.048 | -0.137 | -0.137 | 0.031 |
| Ratio (p-NF-κB p65/GAPDH) | 0.494 | -0.021 | -0.247 | -0.042 | -0.105 | 0.135 | -0.207 | -0.033 |
| Ratio (NF-κB p65/GAPDH) | 0.091 | 0.483 | -0.211 | 0.026 | -0.015 | -0.089 | 0.166 | -0.161 |
| Ratio (p-p38/GAPDH) | 0.109 | -0.329 | 0.218 | -0.045 | 0.159 | 0.437 | -0.169 | 0.141 |
| Ratio (p38/GAPDH) | -0.058 | 0.399 | 0.016 | 0.161 | -0.125 | 0.129 | -0.141 | 0.186 |
| Ratio (p-ERK/GAPDH) | 0.270 | -0.047 | 0.306 | -0.088 | -0.141 | -0.317 | 0.237 | -0.076 |
| Ratio (ERK/GAPDH) | -0.298 | -0.030 | 0.621 | 0.036 | -0.085 | 0.069 | -0.092 | -0.002 |
| Ratio (p-JNK/GAPDH) | 0.069 | 0.102 | 0.072 | 0.103 | 0.311 | -0.070 | 0.079 | -0.209 |
| Ratio (JNK/GAPDH) | 0.229 | 0.023 | 0.095 | 0.031 | 0.221 | -0.168 | 0.013 | 0.062 |
| Ratio (TLR4/GAPDH) | 0.127 | 0.009 | 0.128 | -0.035 | 0.059 | 0.035 | 0.289 | 0.211 |
| Total weight of common factors | 1.876 | 1.842 | 1.799 | 1.535 | 1.476 | 1.467 | 1.384 | 1.172 |

**Table S7**

The scoring coefficient matrix of E4 (10 indicators excluding APTT with the worst correlation, KMO = 0.55 & significance of Bartlett’s Test = 0, initial eigenvalue > 0.65, on behalf of 84.936% raw data).

| Variables | F1 | F2 | F3 | F4 | F5 | F6 |
| --- | --- | --- | --- | --- | --- | --- |
| TT /s | 0.058 | 0.628 | -0.274 | 0.159 | 0.151 | -0.198 |
| PT /s | 0.193 | 0.211 | 0.345 | -0.110 | -0.295 | -0.141 |
| FIB /g·L^-1^ | 0.266 | -0.441 | -0.144 | 0.209 | 0.025 | -0.414 |
| TXB_2_ /pg·mL^-1^ | 0.750 | -0.006 | 0.034 | 0.207 | -0.151 | -0.173 |
| 6-keto-PGF_1α_ /pg·mL^-1^ | 0.147 | -0.001 | -0.047 | 0.731 | -0.064 | 0.096 |
| Ratio (TXB_2_/6-keto-PGF_1α_) | 0.249 | 0.063 | -0.176 | -0.281 | -0.077 | 0.101 |
| PGE_2_ /pg·mL^-1^ | 0.049 | -0.118 | 0.592 | 0.072 | 0.038 | -0.027 |
| ET-1 /pg·mL^-1^ | -0.197 | 0.107 | -0.119 | -0.002 | 0.858 | -0.128 |
| Mg^2+^ /pg·mL^-1^ | 0.329 | -0.228 | 0.429 | -0.186 | 0.239 | 0.028 |
| Ca^2+^ /pg·mL^-1^ | -0.167 | 0.019 | -0.052 | 0.095 | -0.117 | 0.873 |
| Total weight of common factors | 1.567 | 1.555 | 1.507 | 1.393 | 1.262 | 1.209 |

**Table S8**

The scoring coefficient matrix of E5 (15 indicators, KMO = 0.807 & significance of Bartlett’s Test = 0, initial eigenvalue > 0.65, on behalf of 87.993% raw data).

| Variables | F1 | F2 | F3 | F4 | F5 | F6 | F7 |
| --- | --- | --- | --- | --- | --- | --- | --- |
| Organ coefficient of liver /% | -0.158 | -0.131 | -0.053 | 0.017 | 0.851 | 0.160 | -0.017 |
| Organ coefficient of gallbladder /% | -0.168 | -0.038 | -0.130 | -0.030 | -0.062 | 0.046 | 0.973 |
| T-SOD /ng·mL^-1^ | 0.311 | -0.580 | -0.007 | 0.239 | -0.153 | 0.130 | 0.037 |
| MDA /nmol·mL^-1^ | -0.291 | -0.063 | -0.133 | 0.289 | 0.153 | -0.257 | -0.169 |
| GSH /ng·mL^-1^ | -0.078 | -0.035 | 0.149 | 0.023 | -0.075 | 0.041 | -0.029 |
| Fe^3+^ (liver) /μmol·L^-1^ | 0.117 | 0.085 | 0.134 | -0.068 | 0.155 | 0.759 | 0.000 |
| ALT /ng·mL^-1^ | 0.098 | 0.062 | -0.103 | 0.047 | 0.025 | -0.110 | -0.028 |
| AST /ng·mL^-1^ | 0.057 | 0.254 | 0.495 | -0.041 | -0.161 | 0.117 | -0.019 |
| ALP /ng·mL^-1^ | 0.130 | -0.297 | 0.023 | 0.557 | 0.114 | 0.086 | 0.058 |
| Fe^3+^ (serum) /μmol·L^-1^ | 0.441 | -0.068 | 0.723 | -0.102 | 0.123 | -0.156 | -0.018 |
| GST /ng·mL^-1^ | -0.108 | 0.087 | -0.202 | 0.434 | -0.140 | -0.151 | -0.032 |
| GGT /ng·mL^-1^ | 0.195 | -0.001 | -0.032 | -0.033 | 0.029 | -0.020 | 0.064 |
| TBIL /μmol·L^-1^ | -0.134 | 0.629 | 0.154 | -0.029 | -0.142 | 0.245 | 0.060 |
| DBIL /μmol·L^-1^ | 0.318 | -0.096 | 0.017 | 0.066 | -0.043 | 0.042 | 0.007 |
| TBA /μmol·L^-1^ | 0.340 | -0.072 | 0.127 | 0.224 | 0.015 | -0.059 | -0.188 |
| Total weight of common factors | 3.200 | 2.163 | 2.072 | 1.791 | 1.425 | 1.420 | 1.129 |

**Table S9**

Normal distribution test parameters of the integration E1 in each group.

| Groups | *P* value | 99% confidence interval range^#^ | *n*_1_ (before data elimination) | σ_1_ (before data elimination) | *n*_2_ (after data elimination) | σ_2_ (after data elimination) | Comprehensive weight scores (mean) |
| --- | --- | --- | --- | --- | --- | --- | --- |
| Control | 0.200 | 1.73~2.43 | 8 | 0.28 | 8 | 0.28 | 2.08 |
| Model | 0.200 | -7.91~-3.04 | 8 | 1.96 | 8 | 1.96 | -5.48^△△△^ |
| Positive | 0.200 | -3.19~6.48 | 8 | 3.91 | 8 | 3.91 | 1.65*** |
| water-S | 0.200 | -3.75~2.59 | 8 | 2.56 | 8 | 2.56 | -0.58***^△^ |
| water-L | 0.200 | -2.41~1.71 | 8 | 1.67 | 8 | 1.67 | -0.35***^△△^ |
| 10%-S | 0.200 | -0.25~1.98 | 8 | 2.52 | 7 | 0.80 | 0.87***^△△^ |
| 10%-L | 0.111 | -3.11~3.15 | 8 | 2.53 | 8 | 2.53 | 0.02***^△^ |
| 20%-S | 0.171 | -3.69~-4.26 | 8 | 3.44 | 6 | 2.42 | 0.29*** |
| 20%-L | 0.200 | -2.07~3.11 | 8 | 2.09 | 8 | 2.09 | 0.52*** |
| 35%-S | 0.200 | -3.62~3.80 | 8 | 3.00 | 8 | 3.00 | 0.09*** |
| 35%-L | 0.200 | -2.36~3.96 | 8 | 2.55 | 8 | 2.55 | 0.80*** |
| 50%-S | 0.200 | -3.33~2.23 | 8 | 2.25 | 8 | 2.25 | -0.55***^△△^ |
| 50%-L | 0.200 | -3.55~3.81 | 8 | 2.98 | 8 | 2.98 | 0.13*** |
| 65%-S | 0.200 | -5.09~1.42 | 8 | 2.63 | 8 | 2.63 | -1.84**^△△△^ |
| 65%-L | 0.200 | -3.98~3.41 | 8 | 3.30 | 8 | 3.30 | -0.29** |
| 80%-S | 0.200 | -4.23~2.88 | 8 | 3.18 | 8 | 3.18 | -0.67**^△^ |
| 80%-L | 0.200 | -3.43~4.37 | 8 | 3.49 | 8 | 3.49 | 0.47*** |
| 90%-S | 0.200 | -2.71~4.71 | 8 | 3.00 | 8 | 3.00 | 1.00*** |
| 90%-L | 0.200 | -2.46~3.41 | 8 | 2.38 | 8 | 2.38 | 0.47*** |
| ethanol-S | 0.112 | -2.89~5.74 | 8 | 3.69 | 7 | 3.08 | 1.42*** |
| ethanol-L | 0.200 | -2.57~6.95 | 8 | 4.93 | 7 | 3.40 | 2.19*** |

^#^ 99% confidence interval range for guarantee of sample size meeting statistical requirements.

^△^ *P*<0.05, ^△△^ *P*<0.01, ^△△△^ *P*<0.001 compared with the Control;

* *P*<0.05, ** *P*<0.01, *** *P*<0.001 compared with the Model (the same tables below).

**Table S10**

Normal distribution test parameters of the integration E2 in each group.

| Groups | *P* value | 99% confidence interval range^#^ | *n*_1_ (before data elimination) | σ_1_ (before data elimination) | *n*_2_ (after data elimination) | σ_2_ (after data elimination) | Comprehensive weight scores (mean) |
| --- | --- | --- | --- | --- | --- | --- | --- |
| Control | 0.200 | -4.55~-0.52 | 8 | 1.96 | 8 | 1.96 | -2.53 |
| Model | 0.200 | 1.51~9.26 | 8 | 3.77 | 8 | 3.77 | 5.39^△△△^ |
| Positive | 0.200 | -2.16~2.19 | 8 | 2.12 | 8 | 2.12 | 0.01***^△^ |
| water-S | 0.200 | -3.72~3.08 | 8 | 3.04 | 8 | 3.04 | -0.32** |
| water-L | 0.200 | -0.47~0.71 | 8 | 3.06 | 7 | 0.42 | 0.12**^△△^ |
| 10%-S | 0.200 | -2.94~1.28 | 8 | 3.15 | 7 | 1.89 | -0.83*** |
| 10%-L | 0.200 | -0.32~0.93 | 8 | 3.49 | 6 | 0.38 | 0.30**^△△^ |
| 20%-S | 0.200 | -2.66~3.94 | 8 | 3.21 | 8 | 3.21 | 0.64**^△^ |
| 20%-L | 0.200 | -1.40~5.26 | 8 | 2.98 | 8 | 2.98 | 1.93*^△△^ |
| 35%-S | 0.200 | -2.99~5.43 | 8 | 3.76 | 8 | 3.76 | 1.22*^△^ |
| 35%-L | 0.200 | -2.46~5.42 | 8 | 3.52 | 8 | 3.52 | 1.48*^△△^ |
| 50%-S | 0.200 | -3.04~2.83 | 8 | 2.62 | 8 | 2.62 | -0.11**^△^ |
| 50%-L | 0.200 | -1.68~3.87 | 8 | 2.48 | 8 | 2.48 | 1.10*^△△^ |
| 65%-S | 0.200 | -4.55~2.36 | 8 | 3.09 | 8 | 3.09 | -1.10*** |
| 65%-L | 0.200 | -3.04~1.97 | 8 | 3.54 | 7 | 2.24 | -0.53*** |
| 80%-S | 0.149 | -4.26~1.49 | 8 | 3.03 | 7 | 2.57 | -1.38*** |
| 80%-L | 0.200 | -4.49~2.98 | 8 | 3.02 | 8 | 3.02 | -0.76** |
| 90%-S | 0.200 | -4.78~1.92 | 8 | 2.71 | 8 | 2.71 | -1.43*** |
| 90%-L | 0.200 | -3.66~-0.56 | 8 | 1.25 | 8 | 1.25 | -2.11*** |
| ethanol-S | 0.200 | -5.85~-0.93 | 8 | 1.99 | 8 | 1.99 | -3.39*** |
| ethanol-L | 0.142 | -3.36~-1.86 | 8 | 1.79 | 7 | 0.61 | -2.61*** |

**Table S11**

Normal distribution test parameters of the integration E3 in each group.

| Groups | *P* value | 99% confidence interval range^#^ | *n*_1_ (before data elimination) | σ_1_ (before data elimination) | *n*_2_ (after data elimination) | σ_2_ (after data elimination) | Comprehensive weight scores (mean) |
| --- | --- | --- | --- | --- | --- | --- | --- |
| Control | 0.200 | -7.05~4.56 | 6 | 3.53 | 6 | 3.53 | -1.24 |
| Model | 0.200 | -0.09~8.88 | 6 | 3.62 | 6 | 3.62 | 4.40^△^ |
| Positive | 0.200 | -4.70~2.95 | 6 | 2.32 | 6 | 2.32 | -0.88** |
| water-S | 0.200 | -3.11~8.41 | 6 | 3.50 | 6 | 3.50 | 2.65 |
| water-L | 0.200 | -4.76~7.20 | 6 | 4.15 | 5 | 2.90 | 1.22 |
| 10%-S | 0.200 | -2.20~6.74 | 6 | 2.72 | 6 | 2.72 | 2.27 |
| 10%-L | 0.180 | -2.45~4.26 | 6 | 2.04 | 6 | 2.04 | 0.91 |
| 20%-S | 0.200 | -3.89~7.18 | 6 | 3.36 | 6 | 3.36 | 1.65 |
| 20%-L | 0.200 | -6.73~5.09 | 6 | 3.59 | 6 | 3.59 | -0.82* |
| 35%-S | 0.200 | -7.19~5.99 | 6 | 4.00 | 6 | 4.00 | -0.60* |
| 35%-L | 0.200 | -3.46~2.46 | 6 | 4.27 | 5 | 1.44 | -0.50* |
| 50%-S | 0.200 | -6.58~6.04 | 6 | 3.83 | 6 | 3.83 | -0.27* |
| 50%-L | 0.180 | -3.74~2.09 | 6 | 4.28 | 5 | 1.42 | -0.82* |
| 65%-S | 0.200 | -4.30~3.08 | 6 | 4.02 | 5 | 2.24 | -0.61* |
| 65%-L | 0.200 | -5.23~1.40 | 6 | 2.01 | 6 | 2.01 | -1.92** |
| 80%-S | 0.200 | -5.65~5.40 | 6 | 3.36 | 6 | 3.36 | -0.12* |
| 80%-L | 0.200 | -5.03~1.61 | 6 | 2.02 | 6 | 2.02 | -1.71** |
| 90%-S | 0.200 | -6.67~2.17 | 6 | 2.69 | 6 | 2.69 | -2.25** |
| 90%-L | 0.135 | -9.17~4.37 | 6 | 3.69 | 5 | 3.29 | -2.40** |
| ethanol-S | 0.200 | -5.82~1.89 | 6 | 2.34 | 6 | 2.34 | -1.96** |
| ethanol-L | 0.200 | -4.42~1.91 | 6 | 3.99 | 5 | 1.54 | -1.25** |

**Table S12**

Normal distribution test parameters of the integration E4 in each group.

| Groups | *P* value | 99% confidence interval range^#^ | *n*_1_ (before data elimination) | σ_1_ (before data elimination) | *n*_2_ (after data elimination) | σ_2_ (after data elimination) | Comprehensive weight scores (mean) |
| --- | --- | --- | --- | --- | --- | --- | --- |
| Control | 0.200 | 0.90~2.87 | 6 | 0.60 | 6 | 0.60 | 1.89 |
| Model | 0.200 | -3.96~2.18 | 6 | 1.86 | 6 | 1.86 | -0.89^△△^ |
| Positive | 0.200 | -1.24~4.64 | 6 | 1.78 | 6 | 1.78 | 1.70* |
| water-S | 0.200 | -2.30~3.53 | 6 | 1.77 | 6 | 1.77 | 0.62 |
| water-L | 0.200 | -1.93~5.77 | 6 | 2.34 | 6 | 2.34 | 1.92* |
| 10%-S | 0.200 | -4.42~7.63 | 6 | 3.66 | 6 | 3.66 | 1.60 |
| 10%-L | 0.200 | -0.52~2.07 | 6 | 4.26 | 5 | 0.63 | 0.77^△^ |
| 20%-S | 0.200 | -1.96~2.11 | 6 | 1.24 | 6 | 1.24 | 0.08^△△^ |
| 20%-L | 0.200 | -3.69~3.71 | 6 | 3.47 | 5 | 1.80 | 0.01^△^ |
| 35%-S | 0.200 | -7.09~5.13 | 6 | 4.10 | 5 | 2.97 | -0.98^△^ |
| 35%-L | 0.200 | -1.87~1.20 | 6 | 2.45 | 5 | 0.74 | -0.33^△△△^ |
| 50%-S | 0.200 | -8.55~6.67 | 6 | 4.33 | 5 | 3.69 | -0.94 |
| 50%-L | 0.200 | -4.94~4.60 | 6 | 4.04 | 5 | 2.32 | -0.17 |
| 65%-S | 0.200 | -6.25~4.87 | 6 | 3.38 | 6 | 3.38 | -0.69 |
| 65%-L | 0.200 | -4.06~2.74 | 6 | 2.06 | 6 | 2.06 | -0.66^△^ |
| 80%-S | 0.200 | -5.08~4.04 | 6 | 3.95 | 5 | 2.21 | -0.52^△^ |
| 80%-L | 0.200 | -2.91~4.70 | 6 | 2.31 | 6 | 2.31 | 0.89 |
| 90%-S | 0.200 | -0.88~4.04 | 6 | 1.49 | 6 | 1.49 | 1.58* |
| 90%-L | 0.200 | -2.29~3.60 | 6 | 1.79 | 6 | 1.79 | 0.66 |
| ethanol-S | 0.118 | -4.77~7.22 | 6 | 4.19 | 5 | 2.91 | 1.22 |
| ethanol-L | 0.200 | -1.39~3.34 | 6 | 1.44 | 6 | 1.44 | 0.98 |

**Table S13**

Normal distribution test parameters of the integration E5 in each group.

| Groups | *P* value | 99% confidence interval range^#^ | *n*_1_ (before data elimination) | σ_1_ (before data elimination) | *n*_2_ (after data elimination) | σ_2_ (after data elimination) | Comprehensive weight scores (mean) |
| --- | --- | --- | --- | --- | --- | --- | --- |
| Control | 0.200 | -4.52~2.37 | 8 | 2.78 | 8 | 2.78 | -1.07 |
| Model | 0.200 | 2.48~8.32 | 8 | 2.36 | 8 | 2.36 | 5.40^△△△^ |
| Positive | 0.200 | -4.81~3.84 | 8 | 4.18 | 7 | 3.09 | -0.48** |
| water-S | 0.199 | 1.70~5.50 | 8 | 1.53 | 8 | 1.53 | 3.60^△△△^ |
| water-L | 0.200 | -1.43~6.82 | 8 | 3.69 | 8 | 3.69 | 2.70^△^ |
| 10%-S | 0.200 | 1.02~5.41 | 8 | 1.77 | 8 | 1.77 | 3.22^△△^ |
| 10%-L | 0.200 | -3.85~6.06 | 8 | 5.02 | 7 | 4.00 | 1.10* |
| 20%-S | 0.200 | -2.76~5.31 | 8 | 3.26 | 8 | 3.26 | 1.27* |
| 20%-L | 0.200 | -2.73~3.64 | 8 | 2.58 | 8 | 2.58 | 0.45** |
| 35%-S | 0.200 | -4.40~5.10 | 8 | 3.84 | 8 | 3.84 | 0.35** |
| 35%-L | 0.200 | -3.11~1.17 | 8 | 1.73 | 8 | 1.73 | -0.97*** |
| 50%-S | 0.200 | -5.67~5.24 | 8 | 4.80 | 7 | 3.89 | -0.21** |
| 50%-L | 0.200 | -3.84~1.82 | 8 | 2.29 | 8 | 2.29 | -1.01*** |
| 65%-S | 0.120 | -3.68~3.03 | 8 | 4.41 | 7 | 2.39 | -0.32*** |
| 65%-L | 0.200 | -5.62~2.53 | 8 | 3.29 | 8 | 3.29 | -1.55*** |
| 80%-S | 0.200 | -6.24~6.52 | 8 | 6.40 | 6 | 3.88 | 0.14** |
| 80%-L | 0.200 | -6.09~5.35 | 8 | 5.98 | 6 | 3.48 | -0.37** |
| 90%-S | 0.200 | -5.40~3.89 | 8 | 3.76 | 8 | 3.76 | -0.76** |
| 90%-L | 0.200 | -4.13~3.29 | 8 | 3.00 | 8 | 3.00 | -0.42*** |
| ethanol-S | 0.142 | -5.26~4.28 | 8 | 3.86 | 8 | 3.86 | -0.49** |
| ethanol-L | 0.200 | -5.47~5.63 | 8 | 5.62 | 7 | 3.96 | 0.08** |

**Table S14**

The predicted (P), true (T) and their relative error (R) values of test samples in single, double and three hidden layers.

| Runs | Single hidden layer | | | | | | | | | Double hidden layers | | | | | | | | | Three hidden layers | | | | | | | | |
| --- | --- | --- | --- | --- | --- | --- | --- | --- | --- | --- | --- | --- | --- | --- | --- | --- | --- | --- | --- | --- | --- | --- | --- | --- | --- | --- | --- |
|  | 90% EW-L group | | | ethanol-S group | | | ethanol-L group | | | 90% EW-L group | | | ethanol-S group | | | ethanol-L group | | | 90% EW-L group | | | ethanol-S group | | | ethanol-L group | | |
|  | P | T | R | P | T | R | P | T | R | P | T | R | P | T | R | P | T | R | P | T | R | P | T | R | P | T | R |
| 1 | 0.669 | 0.475 | 0.406 | 2.374 | 1.424 | 0.668 | 2.374 | 2.188 | 0.085 | 1.133 | 0.475 | 1.384 | 1.783 | 1.424 | 0.252 | 2.254 | 2.188 | 0.030 | 0.868 | 0.475 | 0.827 | 0.716 | 1.424 | 0.497 | 0.717 | 2.188 | 0.672 |
| 2 | 0.313 | 0.475 | 0.342 | 1.023 | 1.424 | 0.282 | 1.956 | 2.188 | 0.106 | -0.530 | 0.475 | 2.114 | -0.564 | 1.424 | 1.396 | -0.585 | 2.188 | 1.267 | -0.239 | 0.475 | 1.503 | 1.513 | 1.424 | 0.063 | 1.513 | 2.188 | 0.308 |
| 3 | 0.813 | 0.475 | 0.710 | 0.929 | 1.424 | 0.347 | 0.953 | 2.188 | 0.565 | 1.286 | 0.475 | 1.704 | 1.406 | 1.424 | 0.012 | 1.402 | 2.188 | 0.359 | 0.291 | 0.475 | 0.388 | 0.755 | 1.424 | 0.470 | 1.717 | 2.188 | 0.215 |
| 4 | 1.043 | 0.475 | 1.195 | 1.323 | 1.424 | 0.071 | 1.179 | 2.188 | 0.461 | 0.842 | 0.475 | 0.772 | 0.813 | 1.424 | 0.429 | 0.796 | 2.188 | 0.636 | -0.114 | 0.475 | 1.241 | 1.924 | 1.424 | 0.352 | 2.066 | 2.188 | 0.056 |
| 5 | 0.474 | 0.475 | 0.002 | 0.825 | 1.424 | 0.420 | 0.920 | 2.188 | 0.580 | 0.648 | 0.475 | 0.364 | 1.463 | 1.424 | 0.027 | 1.403 | 2.188 | 0.359 | 1.467 | 0.475 | 2.086 | 0.778 | 1.424 | 0.454 | 1.431 | 2.188 | 0.346 |
| 6 | 2.654 | 0.475 | 4.583 | 1.024 | 1.424 | 0.281 | 2.102 | 2.188 | 0.039 | 1.144 | 0.475 | 1.405 | 1.153 | 1.424 | 0.190 | 1.345 | 2.188 | 0.385 | 0.930 | 0.475 | 0.956 | 1.632 | 1.424 | 0.147 | 1.632 | 2.188 | 0.254 |
| 7 | 0.452 | 0.475 | 0.049 | 1.501 | 1.424 | 0.055 | 2.080 | 2.188 | 0.049 | 0.739 | 0.475 | 0.554 | 1.410 | 1.424 | 0.010 | 0.777 | 2.188 | 0.645 | -0.443 | 0.475 | 1.931 | -1.495 | 1.424 | 2.050 | -1.509 | 2.188 | 1.690 |
| 8 | 0.269 | 0.475 | 0.435 | -2.344 | 1.424 | 2.646 | -1.085 | 2.188 | 1.496 | 0.555 | 0.475 | 0.168 | 1.870 | 1.424 | 0.314 | 1.954 | 2.188 | 0.107 | 0.473 | 0.475 | 0.006 | 1.638 | 1.424 | 0.151 | 2.214 | 2.188 | 0.012 |
| 9 | 0.724 | 0.475 | 0.522 | 0.725 | 1.424 | 0.490 | 0.791 | 2.188 | 0.638 | 0.777 | 0.475 | 0.635 | 0.881 | 1.424 | 0.381 | 0.873 | 2.188 | 0.601 | 1.506 | 0.475 | 2.167 | 2.132 | 1.424 | 0.498 | 2.132 | 2.188 | 0.025 |
| 10 | 0.363 | 0.475 | 0.236 | 3.531 | 1.424 | 1.481 | 3.506 | 2.188 | 0.603 | -1.246 | 0.475 | 3.622 | 1.694 | 1.424 | 0.190 | 0.996 | 2.188 | 0.544 | 0.351 | 0.475 | 0.261 | 1.653 | 1.424 | 0.161 | 1.712 | 2.188 | 0.217 |
| 11 | -0.187 | 0.475 | 1.394 | 1.551 | 1.424 | 0.090 | 2.136 | 2.188 | 0.024 | 0.244 | 0.475 | 0.488 | 1.655 | 1.424 | 0.163 | 2.029 | 2.188 | 0.072 | -0.478 | 0.475 | 2.006 | 2.100 | 1.424 | 0.475 | 2.095 | 2.188 | 0.042 |
| 12 | 0.669 | 0.475 | 0.406 | 2.374 | 1.424 | 0.668 | 2.374 | 2.188 | 0.085 | 0.925 | 0.475 | 0.946 | 1.629 | 1.424 | 0.144 | 2.691 | 2.188 | 0.230 | 0.524 | 0.475 | 0.103 | 1.079 | 1.424 | 0.242 | 1.079 | 2.188 | 0.507 |
| 13 | 0.313 | 0.475 | 0.342 | 1.023 | 1.424 | 0.282 | 1.956 | 2.188 | 0.106 | 1.044 | 0.475 | 1.196 | 1.403 | 1.424 | 0.014 | 1.348 | 2.188 | 0.384 | 0.433 | 0.475 | 0.090 | 0.433 | 1.424 | 0.696 | 0.433 | 2.188 | 0.802 |
| 14 | 0.813 | 0.475 | 0.710 | 0.929 | 1.424 | 0.347 | 0.953 | 2.188 | 0.565 | 0.977 | 0.475 | 1.054 | 0.777 | 1.424 | 0.454 | 0.881 | 2.188 | 0.597 | -0.175 | 0.475 | 1.369 | 1.705 | 1.424 | 0.198 | 1.741 | 2.188 | 0.204 |
| 15 | 1.043 | 0.475 | 1.195 | 1.323 | 1.424 | 0.071 | 1.179 | 2.188 | 0.461 | -0.073 | 0.475 | 1.153 | -0.641 | 1.424 | 1.450 | 0.591 | 2.188 | 0.730 | 1.175 | 0.475 | 1.472 | 0.738 | 1.424 | 0.481 | 0.701 | 2.188 | 0.680 |
| 16 | 0.474 | 0.475 | 0.002 | 0.825 | 1.424 | 0.420 | 0.920 | 2.188 | 0.580 | 0.188 | 0.475 | 0.605 | 0.384 | 1.424 | 0.730 | 0.891 | 2.188 | 0.593 | 0.861 | 0.475 | 0.810 | 1.088 | 1.424 | 0.236 | 1.074 | 2.188 | 0.509 |
| 17 | 2.654 | 0.475 | 4.583 | 1.024 | 1.424 | 0.281 | 2.102 | 2.188 | 0.039 | 1.350 | 0.475 | 1.841 | 2.000 | 1.424 | 0.405 | 2.021 | 2.188 | 0.076 | 0.724 | 0.475 | 0.523 | 0.600 | 1.424 | 0.578 | 0.600 | 2.188 | 0.726 |
| 18 | 0.452 | 0.475 | 0.049 | 1.501 | 1.424 | 0.055 | 2.080 | 2.188 | 0.049 | 1.000 | 0.475 | 1.103 | 1.346 | 1.424 | 0.054 | 1.343 | 2.188 | 0.386 | 0.600 | 0.475 | 0.262 | 1.494 | 1.424 | 0.049 | 1.494 | 2.188 | 0.317 |
| 19 | 0.269 | 0.475 | 0.435 | -2.344 | 1.424 | 2.646 | -1.085 | 2.188 | 1.496 | 1.535 | 0.475 | 2.229 | 1.812 | 1.424 | 0.273 | 1.854 | 2.188 | 0.152 | 1.002 | 0.475 | 1.108 | 1.074 | 1.424 | 0.245 | 1.076 | 2.188 | 0.508 |
| 20 | 0.724 | 0.475 | 0.522 | 0.725 | 1.424 | 0.490 | 0.791 | 2.188 | 0.638 | 1.550 | 0.475 | 2.260 | 1.650 | 1.424 | 0.159 | 1.627 | 2.188 | 0.256 | 0.941 | 0.475 | 0.980 | 0.826 | 1.424 | 0.420 | 0.835 | 2.188 | 0.618 |

**Table S15**

The predicted (P), true (T) and their relative error (R) values of test samples in a different number of neurons per hidden layer.

| No. of neurons | Runs | 1 | | | 2 | | | 3 | | | 4 | | | 5 | | |
| --- | --- | --- | --- | --- | --- | --- | --- | --- | --- | --- | --- | --- | --- | --- | --- | --- |
|  | Groups | 90% EW-L | ethanol-S | ethanol-L | 90% EW-L | ethanol-S | ethanol-L | 90% EW-L | ethanol-S | ethanol-L | 90% EW-L | ethanol-S | ethanol-L | 90% EW-L | ethanol-S | ethanol-L |
| 5 | P | 0.093 | -1.275 | 0.073 | -0.318 | 2.380 | 2.376 | 0.597 | 0.657 | 0.548 | 0.671 | 4.497 | 4.388 | 0.484 | 1.421 | 1.446 |
|  | T | 0.475 | 1.424 | 2.188 | 0.475 | 1.424 | 2.188 | 0.475 | 1.424 | 2.188 | 0.475 | 1.424 | 2.188 | 0.475 | 1.424 | 2.188 |
|  | R | 0.804 | 1.895 | 0.966 | 1.668 | 0.672 | 0.086 | 0.256 | 0.538 | 0.749 | 0.411 | 2.159 | 1.006 | 0.018 | 0.002 | 0.339 |
| 10 | P | 1.159 | 1.305 | 1.547 | 2.201 | 2.337 | 2.337 | 0.407 | 2.045 | 2.147 | 0.839 | 1.060 | 1.060 | 0.066 | 1.127 | 1.147 |
|  | T | 0.475 | 1.424 | 2.188 | 0.475 | 1.424 | 2.188 | 0.475 | 1.424 | 2.188 | 0.475 | 1.424 | 2.188 | 0.475 | 1.424 | 2.188 |
|  | R | 1.438 | 0.083 | 0.293 | 3.630 | 0.642 | 0.068 | 0.145 | 0.437 | 0.019 | 0.765 | 0.256 | 0.516 | 0.861 | 0.208 | 0.476 |
| 15 | P | -0.050 | 2.630 | -0.272 | 0.588 | 1.350 | 0.197 | 0.645 | 1.829 | 1.639 | -0.446 | 2.646 | 2.593 | -0.065 | 1.166 | 1.522 |
|  | T | 0.475 | 1.424 | 2.188 | 0.475 | 1.424 | 2.188 | 0.475 | 1.424 | 2.188 | 0.475 | 1.424 | 2.188 | 0.475 | 1.424 | 2.188 |
|  | R | 1.105 | 0.848 | 1.124 | 0.237 | 0.051 | 0.910 | 0.356 | 0.285 | 0.251 | 1.938 | 0.859 | 0.185 | 1.136 | 0.181 | 0.304 |
| 20 | P | 0.568 | 1.501 | 2.243 | 0.720 | 1.696 | 1.697 | 0.746 | 1.500 | 1.487 | 1.684 | 1.359 | 1.363 | 0.280 | 1.140 | 1.288 |
|  | T | 0.475 | 1.424 | 2.188 | 0.475 | 1.424 | 2.188 | 0.475 | 1.424 | 2.188 | 0.475 | 1.424 | 2.188 | 0.475 | 1.424 | 2.188 |
|  | R | 0.194 | 0.054 | 0.025 | 0.515 | 0.192 | 0.224 | 0.569 | 0.054 | 0.320 | 2.542 | 0.046 | 0.377 | 0.410 | 0.200 | 0.411 |
| 40 | P | -0.322 | 0.893 | 0.900 | 0.709 | 8.725 | 7.155 | -0.033 | 1.004 | 0.769 | 0.828 | 1.580 | 1.578 | -0.517 | 2.158 | 1.605 |
|  | T | 0.475 | 1.424 | 2.188 | 0.475 | 1.424 | 2.188 | 0.475 | 1.424 | 2.188 | 0.475 | 1.424 | 2.188 | 0.475 | 1.424 | 2.188 |
|  | R | 1.678 | 0.373 | 0.589 | 0.491 | 5.129 | 2.271 | 1.069 | 0.295 | 0.648 | 0.742 | 0.110 | 0.279 | 2.087 | 0.516 | 0.266 |
| 60 | P | 1.137 | 1.508 | 1.462 | 0.671 | 1.433 | 1.433 | 1.234 | 3.747 | 4.296 | 0.981 | 1.925 | 1.640 | -0.094 | -1.183 | -2.040 |
|  | T | 0.475 | 1.424 | 2.188 | 0.475 | 1.424 | 2.188 | 0.475 | 1.424 | 2.188 | 0.475 | 1.424 | 2.188 | 0.475 | 1.424 | 2.188 |
|  | R | 1.391 | 0.060 | 0.332 | 0.411 | 0.007 | 0.345 | 1.595 | 1.632 | 0.964 | 1.064 | 0.352 | 0.250 | 1.198 | 1.831 | 1.932 |
| 70 | P | 1.426 | 1.689 | 1.682 | 0.691 | 1.939 | 2.064 | -0.596 | 2.919 | 3.056 | -1.437 | 0.370 | 0.297 | 1.330 | 1.721 | 1.745 |
|  | T | 0.475 | 1.424 | 2.188 | 0.475 | 1.424 | 2.188 | 0.475 | 1.424 | 2.188 | 0.475 | 1.424 | 2.188 | 0.475 | 1.424 | 2.188 |
|  | R | 1.999 | 0.186 | 0.231 | 0.454 | 0.362 | 0.056 | 2.254 | 1.051 | 0.397 | 4.023 | 0.740 | 0.864 | 1.797 | 0.209 | 0.202 |
| 75 | P | -4.928 | -7.662 | -4.662 | -0.226 | 1.124 | 1.053 | 1.347 | 1.900 | 1.775 | -0.795 | 1.290 | 1.317 | -3.324 | -10.446 | -5.182 |
|  | T | 0.475 | 1.424 | 2.188 | 0.475 | 1.424 | 2.188 | 0.475 | 1.424 | 2.188 | 0.475 | 1.424 | 2.188 | 0.475 | 1.424 | 2.188 |
|  | R | 11.366 | 6.383 | 3.131 | 1.476 | 0.210 | 0.519 | 1.833 | 0.335 | 0.188 | 2.673 | 0.094 | 0.398 | 7.992 | 8.338 | 3.369 |
| 80 | P | 0.328 | 5.055 | 4.429 | 0.742 | 1.469 | 0.194 | 1.434 | 1.468 | 1.935 | 0.424 | 0.373 | 0.083 | 4.738 | 8.925 | 8.373 |
|  | T | 0.475 | 1.424 | 2.188 | 0.475 | 1.424 | 2.188 | 0.475 | 1.424 | 2.188 | 0.475 | 1.424 | 2.188 | 0.475 | 1.424 | 2.188 |
|  | R | 0.311 | 2.551 | 1.025 | 0.561 | 0.032 | 0.911 | 2.017 | 0.031 | 0.115 | 0.109 | 0.738 | 0.962 | 8.966 | 5.269 | 2.828 |
| 100 | P | 5.754 | 5.384 | -0.079 | -4.070 | -6.232 | -3.769 | 0.034 | 3.858 | 4.104 | 1.067 | 2.199 | 2.247 | -8.494 | -8.200 | -9.022 |
|  | T | 0.475 | 1.424 | 2.188 | 0.475 | 1.424 | 2.188 | 0.475 | 1.424 | 2.188 | 0.475 | 1.424 | 2.188 | 0.475 | 1.424 | 2.188 |
|  | R | 11.104 | 2.782 | 1.036 | 9.561 | 5.378 | 2.723 | 0.929 | 1.710 | 0.876 | 1.244 | 0.545 | 0.027 | 18.867 | 6.761 | 5.124 |
| 130 | P | 0.528 | 0.533 | 0.533 | 3.051 | 1.145 | 0.796 | 0.487 | 1.422 | 2.159 | 4.425 | 4.455 | 4.457 | 6.013 | 3.203 | 3.474 |
|  | T | 0.475 | 1.424 | 2.188 | 0.475 | 1.424 | 2.188 | 0.475 | 1.424 | 2.188 | 0.475 | 1.424 | 2.188 | 0.475 | 1.424 | 2.188 |
|  | R | 0.111 | 0.626 | 0.756 | 5.417 | 0.195 | 0.636 | 0.025 | 0.001 | 0.013 | 8.309 | 2.130 | 1.037 | 11.649 | 1.250 | 0.588 |
| 160 | P | 1.345 | 1.325 | 1.165 | 0.640 | 1.637 | 1.830 | 3.167 | 2.415 | 7.978 | 0.530 | 1.825 | 3.713 | 1.324 | 2.429 | 3.018 |
|  | T | 0.475 | 1.424 | 2.188 | 0.475 | 1.424 | 2.188 | 0.475 | 1.424 | 2.188 | 0.475 | 1.424 | 2.188 | 0.475 | 1.424 | 2.188 |
|  | R | 1.829 | 0.070 | 0.467 | 0.347 | 0.150 | 0.163 | 5.662 | 0.696 | 2.647 | 0.115 | 0.282 | 0.697 | 1.785 | 0.706 | 0.380 |
| 190 | P | 1.023 | 1.337 | 2.586 | -0.046 | 1.959 | 2.347 | 1.289 | 1.782 | 1.648 | -2.078 | -4.031 | 0.867 | 2.787 | 11.829 | 11.326 |
|  | T | 0.475 | 1.424 | 2.188 | 0.475 | 1.424 | 2.188 | 0.475 | 1.424 | 2.188 | 0.475 | 1.424 | 2.188 | 0.475 | 1.424 | 2.188 |
|  | R | 1.151 | 0.061 | 0.182 | 1.097 | 0.376 | 0.073 | 1.711 | 0.252 | 0.247 | 5.371 | 3.832 | 0.604 | 4.863 | 7.310 | 4.177 |

**Table S16**

The predicted (P), true (T) and their relative error (R) values of test samples in different learning rates.

| Learning rates | Runs | 1 | | | 2 | | | 3 | | | 4 | | | 5 | | |
| --- | --- | --- | --- | --- | --- | --- | --- | --- | --- | --- | --- | --- | --- | --- | --- | --- |
|  | Groups | 90% EW-L | ethanol-S | ethanol-L | 90% EW-L | ethanol-S | ethanol-L | 90% EW-L | ethanol-S | ethanol-L | 90% EW-L | ethanol-S | ethanol-L | 90% EW-L | ethanol-S | ethanol-L |
| 0.01 | P | 0.504 | 1.901 | 1.902 | -0.807 | -0.374 | 0.393 | 1.101 | -0.412 | -0.812 | 0.310 | 0.526 | 0.509 | 1.122 | 0.349 | 0.235 |
|  | T | 0.475 | 1.424 | 2.188 | 0.475 | 1.424 | 2.188 | 0.475 | 1.424 | 2.188 | 0.475 | 1.424 | 2.188 | 0.475 | 1.424 | 2.188 |
|  | R | 0.061 | 0.336 | 0.131 | 2.697 | 1.263 | 0.820 | 1.316 | 1.289 | 1.371 | 0.347 | 0.631 | 0.767 | 1.360 | 0.755 | 0.893 |
| 0.02 | P | 0.895 | 2.121 | 2.239 | 0.692 | 0.367 | 0.224 | 0.269 | 1.398 | 2.331 | 3.115 | 2.233 | 2.311 | 0.612 | 1.429 | 2.074 |
|  | T | 0.475 | 1.424 | 2.188 | 0.475 | 1.424 | 2.188 | 0.475 | 1.424 | 2.188 | 0.475 | 1.424 | 2.188 | 0.475 | 1.424 | 2.188 |
|  | R | 0.882 | 0.490 | 0.024 | 0.456 | 0.742 | 0.898 | 0.433 | 0.018 | 0.066 | 5.553 | 0.569 | 0.056 | 0.288 | 0.004 | 0.052 |
| 0.03 | P | 0.343 | 1.645 | 1.717 | 0.768 | 2.250 | 2.251 | 1.003 | 0.952 | 0.904 | 0.984 | 1.836 | 1.467 | 0.779 | 2.076 | 2.076 |
|  | T | 0.475 | 1.424 | 2.188 | 0.475 | 1.424 | 2.188 | 0.475 | 1.424 | 2.188 | 0.475 | 1.424 | 2.188 | 0.475 | 1.424 | 2.188 |
|  | R | 0.278 | 0.156 | 0.215 | 0.616 | 0.580 | 0.029 | 1.109 | 0.331 | 0.587 | 1.070 | 0.290 | 0.329 | 0.639 | 0.459 | 0.051 |
| 0.04 | P | 1.586 | 2.317 | 2.304 | 0.890 | 2.498 | 2.520 | -0.700 | 1.817 | 2.058 | -0.575 | 4.090 | 3.376 | 1.546 | 1.900 | 1.678 |
|  | T | 0.475 | 1.424 | 2.188 | 0.475 | 1.424 | 2.188 | 0.475 | 1.424 | 2.188 | 0.475 | 1.424 | 2.188 | 0.475 | 1.424 | 2.188 |
|  | R | 2.337 | 0.628 | 0.053 | 0.872 | 0.755 | 0.152 | 2.473 | 0.276 | 0.059 | 2.210 | 1.873 | 0.543 | 2.252 | 0.334 | 0.233 |
| 0.05 | P | -0.854 | 0.811 | 0.800 | -0.958 | -0.440 | 1.278 | 0.832 | 1.596 | 1.626 | 0.243 | 1.387 | 1.635 | -0.056 | 1.231 | 1.237 |
|  | T | 0.475 | 1.424 | 2.188 | 0.475 | 1.424 | 2.188 | 0.475 | 1.424 | 2.188 | 0.475 | 1.424 | 2.188 | 0.475 | 1.424 | 2.188 |
|  | R | 2.797 | 0.430 | 0.634 | 3.015 | 1.309 | 0.416 | 0.749 | 0.121 | 0.257 | 0.488 | 0.026 | 0.253 | 1.118 | 0.135 | 0.434 |
| 0.06 | P | 0.478 | 1.534 | 1.870 | -0.226 | 1.686 | 1.302 | 1.836 | 1.721 | 1.010 | 1.908 | 1.536 | 1.332 | -0.567 | 0.544 | 0.681 |
|  | T | 0.475 | 1.424 | 2.188 | 0.475 | 1.424 | 2.188 | 0.475 | 1.424 | 2.188 | 0.475 | 1.424 | 2.188 | 0.475 | 1.424 | 2.188 |
|  | R | 0.004 | 0.077 | 0.145 | 1.476 | 0.185 | 0.405 | 2.862 | 0.209 | 0.538 | 3.014 | 0.079 | 0.391 | 2.192 | 0.618 | 0.689 |
| 0.07 | P | 0.587 | 1.318 | 1.424 | 0.118 | 1.571 | 1.634 | 0.665 | 1.196 | 0.709 | -0.269 | 3.119 | 3.048 | 1.091 | 1.214 | 1.819 |
|  | T | 0.475 | 1.424 | 2.188 | 0.475 | 1.424 | 2.188 | 0.475 | 1.424 | 2.188 | 0.475 | 1.424 | 2.188 | 0.475 | 1.424 | 2.188 |
|  | R | 0.236 | 0.074 | 0.349 | 0.752 | 0.104 | 0.253 | 0.399 | 0.160 | 0.676 | 1.565 | 1.191 | 0.394 | 1.294 | 0.148 | 0.168 |
| 0.08 | P | -0.914 | 2.179 | 2.025 | 0.722 | 2.092 | 2.092 | 1.467 | 4.354 | 4.346 | 1.820 | 1.457 | 1.973 | 0.830 | 2.550 | 2.585 |
|  | T | 0.475 | 1.424 | 2.188 | 0.475 | 1.424 | 2.188 | 0.475 | 1.424 | 2.188 | 0.475 | 1.424 | 2.188 | 0.475 | 1.424 | 2.188 |
|  | R | 2.922 | 0.530 | 0.074 | 0.518 | 0.470 | 0.044 | 2.087 | 2.059 | 0.987 | 2.828 | 0.023 | 0.098 | 0.747 | 0.791 | 0.182 |
| 0.09 | P | 0.190 | 6.010 | 5.901 | -0.057 | 1.184 | 2.051 | 2.369 | 1.906 | 1.469 | 0.992 | 0.245 | 1.240 | 0.334 | 1.422 | 1.389 |
|  | T | 0.475 | 1.424 | 2.188 | 0.475 | 1.424 | 2.188 | 0.475 | 1.424 | 2.188 | 0.475 | 1.424 | 2.188 | 0.475 | 1.424 | 2.188 |
|  | R | 0.599 | 3.222 | 1.697 | 1.120 | 0.168 | 0.063 | 3.983 | 0.339 | 0.328 | 1.086 | 0.828 | 0.433 | 0.297 | 0.001 | 0.365 |
| 0.1 | P | 0.966 | 0.965 | 1.027 | 0.906 | 1.915 | 2.469 | 0.003 | 1.584 | 2.036 | 2.262 | 2.332 | 2.337 | 0.063 | 1.609 | 1.649 |
|  | T | 0.475 | 1.424 | 2.188 | 0.475 | 1.424 | 2.188 | 0.475 | 1.424 | 2.188 | 0.475 | 1.424 | 2.188 | 0.475 | 1.424 | 2.188 |
|  | R | 1.032 | 0.322 | 0.531 | 0.905 | 0.345 | 0.129 | 0.993 | 0.113 | 0.069 | 3.758 | 0.638 | 0.068 | 0.868 | 0.130 | 0.246 |
| 0.2 | P | 0.590 | 1.934 | 1.481 | 0.700 | 1.236 | 2.004 | 0.700 | 0.329 | 0.341 | 0.691 | 0.673 | 0.879 | 0.505 | 1.455 | 0.916 |
|  | T | 0.475 | 1.424 | 2.188 | 0.475 | 1.424 | 2.188 | 0.475 | 1.424 | 2.188 | 0.475 | 1.424 | 2.188 | 0.475 | 1.424 | 2.188 |
|  | R | 0.240 | 0.359 | 0.323 | 0.472 | 0.132 | 0.084 | 0.473 | 0.769 | 0.844 | 0.453 | 0.528 | 0.598 | 0.061 | 0.022 | 0.581 |
| 0.3 | P | -1.544 | 1.292 | 1.268 | 0.781 | 1.378 | 2.294 | 1.301 | -0.622 | 1.203 | 1.288 | 1.740 | 1.767 | 1.336 | 1.601 | 1.798 |
|  | T | 0.475 | 1.424 | 2.188 | 0.475 | 1.424 | 2.188 | 0.475 | 1.424 | 2.188 | 0.475 | 1.424 | 2.188 | 0.475 | 1.424 | 2.188 |
|  | R | 4.247 | 0.092 | 0.420 | 0.643 | 0.032 | 0.049 | 1.738 | 1.437 | 0.450 | 1.709 | 0.223 | 0.192 | 1.810 | 0.125 | 0.178 |
| 0.4 | P | 1.000 | 2.687 | 3.043 | -0.776 | -0.159 | -0.538 | 2.216 | 3.516 | 3.272 | 0.129 | 2.136 | 2.136 | -1.151 | 0.346 | 0.601 |
|  | T | 0.475 | 1.424 | 2.188 | 0.475 | 1.424 | 2.188 | 0.475 | 1.424 | 2.188 | 0.475 | 1.424 | 2.188 | 0.475 | 1.424 | 2.188 |
|  | R | 1.105 | 0.888 | 0.391 | 2.632 | 1.111 | 1.246 | 3.662 | 1.470 | 0.496 | 0.730 | 0.500 | 0.024 | 3.422 | 0.757 | 0.725 |
| 0.5 | P | 0.017 | 0.349 | 1.063 | 0.342 | 1.129 | 1.081 | 0.406 | 1.877 | 1.821 | 1.871 | 2.719 | 2.718 | 1.241 | 1.526 | 1.551 |
|  | T | 0.475 | 1.424 | 2.188 | 0.475 | 1.424 | 2.188 | 0.475 | 1.424 | 2.188 | 0.475 | 1.424 | 2.188 | 0.475 | 1.424 | 2.188 |
|  | R | 0.963 | 0.755 | 0.514 | 0.281 | 0.207 | 0.506 | 0.146 | 0.319 | 0.168 | 2.935 | 0.910 | 0.242 | 1.611 | 0.072 | 0.291 |
| 0.6 | P | 3.036 | 3.764 | 3.817 | 0.518 | 2.884 | 2.161 | 0.589 | 1.702 | 1.949 | -0.190 | 3.307 | 3.369 | 2.567 | 2.361 | 2.361 |
|  | T | 0.475 | 1.424 | 2.188 | 0.475 | 1.424 | 2.188 | 0.475 | 1.424 | 2.188 | 0.475 | 1.424 | 2.188 | 0.475 | 1.424 | 2.188 |
|  | R | 5.387 | 1.644 | 0.745 | 0.089 | 1.026 | 0.012 | 0.240 | 0.196 | 0.109 | 1.400 | 1.323 | 0.540 | 4.400 | 0.659 | 0.079 |
| 0.7 | P | 2.148 | 2.142 | 2.143 | 0.647 | 1.596 | 1.570 | 0.472 | 1.175 | 1.307 | 0.545 | 1.415 | 1.693 | 1.740 | 2.868 | 3.113 |
|  | T | 0.475 | 1.424 | 2.188 | 0.475 | 1.424 | 2.188 | 0.475 | 1.424 | 2.188 | 0.475 | 1.424 | 2.188 | 0.475 | 1.424 | 2.188 |
|  | R | 3.519 | 0.505 | 0.020 | 0.361 | 0.121 | 0.282 | 0.008 | 0.174 | 0.403 | 0.147 | 0.006 | 0.226 | 2.661 | 1.015 | 0.423 |
| 0.8 | P | 0.706 | 1.021 | 0.994 | 0.872 | 0.827 | 0.844 | 0.526 | 1.541 | 2.176 | 0.242 | 1.330 | 1.396 | 0.429 | 2.003 | 2.178 |
|  | T | 0.475 | 1.424 | 2.188 | 0.475 | 1.424 | 2.188 | 0.475 | 1.424 | 2.188 | 0.475 | 1.424 | 2.188 | 0.475 | 1.424 | 2.188 |
|  | R | 0.484 | 0.283 | 0.546 | 0.834 | 0.419 | 0.614 | 0.106 | 0.083 | 0.005 | 0.491 | 0.066 | 0.362 | 0.098 | 0.407 | 0.005 |

**Table S17**

Decision weights over 0.01 of rhubarb components ranking by their total weights (the ordinal number after the values represents the top 10 order in each effect respectively; weights < 0.01 are ignored by “-”).

| No. | E1 | E2 | E3 | E4 | E5 | Total weights |
| --- | --- | --- | --- | --- | --- | --- |
| **42** | 0.01966 ^(1)^ | 0.01213 ^(1)^ | 0.01190 | 0.01181 | 0.01133 | 0.06683 |
| **36** | 0.01290 ^(9)^ | 0.01070 | 0.01025 | 0.01002 | 0.01016 | 0.05403 |
| **46** | 0.01431 ^(6)^ | - | 0.01219 | 0.01012 | 0.01110 | 0.04772 |
| **92** | - | 0.01082 | 0.01088 | 0.01024 | 0.01029 | 0.04223 |
| **35** | 0.01770 ^(3)^ | 0.01115 | - | - | 0.01192 ^(8)^ | 0.04077 |
| **55** | 0.01058 | - | 0.01249 ^(10)^ | - | 0.01448 ^(2)^ | 0.03755 |
| **81** | - | 0.01101 | - | 0.01458 ^(2)^ | 0.01023 | 0.03582 |
| **72** | 0.01084 | - | 0.01329 ^(5)^ | - | 0.01071 | 0.03484 |
| **59** | 0.01280 | - | 0.01043 | - | 0.01157 | 0.03480 |
| **33** | - | 0.01080 | 0.01132 | - | 0.01267 ^(5)^ | 0.03479 |
| **29** | - | 0.01155 ^(5)^ | - | 0.01240 | 0.01061 | 0.03456 |
| **88** | 0.01186 | - | 0.01113 | - | 0.01122 | 0.03421 |
| **58** | - | 0.01108 | - | 0.01256 ^(10)^ | 0.01006 | 0.03370 |
| **106** | 0.01052 | 0.01075 | - | 0.01154 | - | 0.03281 |
| **107** | 0.01008 | 0.01172 ^(3)^ | - | - | 0.01096 | 0.03276 |
| **90** | 0.01032 | - | 0.01023 | 0.01220 | - | 0.03275 |
| **89** | 0.01160 | - | - | 0.01061 | 0.01051 | 0.03272 |
| **108** | 0.01123 | 0.01127 ^(9)^ | - | - | 0.01004 | 0.03254 |
| **21** | 0.01925 ^(2)^ | - | 0.01243 | - | - | 0.03168 |
| **37** | 0.01484 ^(4)^ | - | 0.01306 ^(6)^ | - | - | 0.02790 |
| **12** | - | - | 0.01358 ^(3)^ | - | 0.01366 ^(4)^ | 0.02724 |
| **34** | 0.01435 ^(5)^ | - | 0.01247 | - | - | 0.02682 |
| **40** | - | 0.01092 | - | 0.01538 ^(1)^ | - | 0.02630 |
| **69** | - | - | - | 0.01410 ^(5)^ | 0.01178 | 0.02588 |
| **18** | 0.01153 | - | 0.01368 ^(2)^ | - | - | 0.02521 |
| **19** | - | 0.01034 | - | - | 0.01486 ^(1)^ | 0.02520 |
| **64** | - | - | - | 0.01333 ^(8)^ | 0.01182 ^(10)^ | 0.02515 |
| **62** | - | - | - | 0.01077 | 0.01423 ^(3)^ | 0.02500 |
| **98** | 0.01307 ^(8)^ | 0.01135 ^(8)^ | - | - | - | 0.02442 |
| **52** | - | - | - | 0.01200 | 0.01224 ^(6)^ | 0.02424 |
| **80** | - | 0.01117 | 0.01281 ^(8)^ | - | - | 0.02398 |
| **83** | - | 0.01157 ^(4)^ | - | 0.01227 | - | 0.02384 |
| **22** | - | - | 0.01174 | - | 0.01199 ^(7)^ | 0.02373 |
| **11** | 0.01316 ^(7)^ | 0.01052 | - | - | - | 0.02368 |
| **27** | 0.01283 ^(10)^ | - | 0.01068 | - | - | 0.02351 |
| **100** | - | 0.01052 | - | 0.01287 ^(9)^ | - | 0.02339 |
| **51** | 0.01042 | - | 0.01293 ^(7)^ | - | - | 0.02335 |
| **28** | - | 0.01145 ^(6)^ | 0.01097 | - | - | 0.02242 |
| **87** | - | - | - | 0.01028 | 0.01185 ^(9)^ | 0.02213 |
| **61** | 0.01048 | 0.01144 ^(7)^ | - | - | - | 0.02192 |
| **95** | 0.01016 | - | - | - | 0.01164 | 0.02180 |
| **67** | - | - | 0.01060 | 0.01113 | - | 0.02173 |
| **71** | - | - | - | 0.01068 | 0.01057 | 0.02125 |
| **6** | 0.01059 | - | 0.01004 | - | - | 0.02063 |
| **45** | 0.01048 | - | - | - | 0.01009 | 0.02057 |
| **24** | - | - | 0.01499 ^(1)^ | - | - | 0.01499 |
| **56** | - | - | - | 0.01453 ^(3)^ | - | 0.01453 |
| **75** | - | - | - | 0.01452 ^(4)^ | - | 0.01452 |
| **53** | - | - | - | 0.01368 ^(6)^ | - | 0.01368 |
| **9** | - | - | - | 0.01341 ^(7)^ | - | 0.01341 |
| **86** | - | - | 0.01341 ^(4)^ | - | - | 0.01341 |
| **26** | - | - | 0.01258 ^(9)^ | - | - | 0.01258 |
| **49** | - | - | - | 0.01241 | - | 0.01241 |
| **74** | - | - | - | 0.01236 | - | 0.01236 |
| **16** | - | - | 0.01223 | - | - | 0.01223 |
| **97** | - | - | 0.01209 | - | - | 0.01209 |
| **63** | - | - | - | 0.01199 | - | 0.01199 |
| **7** | - | 0.01178 ^(2)^ | - | - | - | 0.01178 |
| **66** | - | - | - | 0.01164 | - | 0.01164 |
| **8** | - | 0.01121 ^(10)^ | - | - | - | 0.01121 |
| **78** | 0.01107 | - | - | - | - | 0.01107 |
| **43** | - | - | - | - | 0.01106 | 0.01106 |
| **25** | - | - | - | - | 0.01102 | 0.01102 |
| **105** | 0.01100 | - | - | - | - | 0.01100 |
| **48** | - | - | - | 0.01097 | - | 0.01097 |
| **15** | - | 0.01085 | - | - | - | 0.01085 |
| **31** | - | - | - | 0.01082 | - | 0.01082 |
| **50** | - | 0.01069 | - | - | - | 0.01069 |
| **2** | - | - | 0.01067 | - | - | 0.01067 |
| **79** | - | 0.01060 | - | - | - | 0.01060 |
| **104** | - | - | - | - | 0.01060 | 0.01060 |
| **94** | - | - | - | - | 0.01055 | 0.01055 |
| **99** | - | - | - | - | 0.01047 | 0.01047 |
| **70** | - | - | 0.01046 | - | - | 0.01046 |
| **57** | - | 0.01045 | - | - | - | 0.01045 |
| **32** | 0.01029 | - | - | - | - | 0.01029 |
| **44** | - | - | 0.01025 | - | - | 0.01025 |
| **41** | 0.01024 | - | - | - | - | 0.01024 |
| **1** | - | - | 0.01019 | - | - | 0.01019 |
| **20** | - | - | - | 0.01016 | - | 0.01016 |
| **60** | - | - | - | 0.01015 | - | 0.01015 |
| **38** | 0.01014 | - | - | - | - | 0.01014 |
| **82** | - | - | 0.01006 | - | - | 0.01006 |
| **101** | - | 0.01005 | - | - | - | 0.01005 |
| **39** | - | 0.01004 | - | - | - | 0.01004 |
| **73** | - | - | 0.01003 | - | - | 0.01003 |
| **4** | - | - | 0.01002 | - | - | 0.01002 |
| **30** | - | - | 0.01002 | - | - | 0.01002 |
| **102** | - | - | - | - | 0.01001 | 0.01001 |

**Table S18**

Identificaiton of the top 10 components in total decision weights of five effects.

| Ranking | Weights | No. | MS/ reference CAS | Component name | Structure/ Formula |
| --- | --- | --- | --- | --- | --- |
| 1 | 0.06683 | **42** | 419.134 [M-H]^-^,  259.098 [M-Glc+H]^+^ | Rhapontigenin 3'-*O*-glucoside |  |
| 2 | 0.05403 | **36** | 463.123 [M+H]^+^ | 1,4-Dihydroxy-2-methoxy-3-  methyl-5-[3,4,5-trihydroxy-6-  (hydroxymethyl)oxan-2-yl]  oxyanthracene-9,10-dione |  |
| 3 | 0.04772 | **46** | CAS# 81-27-6 | Sennoside A |  |
| 4 | 0.04223 | **92** | 502.1 [M]^+^, 501.106 [M-H]^-^ | Chrysophanol 8-*O*-*β*-D-(6'-*O*-malonyl)-  glucoside |  |
| 5 | 0.04077 | **35** | 660.104 [M]^-^ | - | - |
| 6 | 0.03755 | **55** | 283.065 [M-Glc-H]^-^ | Rhein/ Physcion glucoside | C_22_H_22_O_10_ |
| 7 | 0.03582 | **81** | 247.097 [M-Glc+H]^+^,  245.081 [M-Glc-H]^-^ | Torachrysone glucoside | C_20_H_24_O_9_ |
| 8 | 0.03484 | **72** | CAS# 4839-60-5 | Chrysophanol 1-*O*-*β*-D-glucoside |  |
| 9 | 0.03480 | **59** | CAS# 94356-33-9 | 5-Acetyl-7-hydroxy-  2-methyl-chromone |  |
| 10 | 0.03479 | **33** | CAS# 59282-56-3 | Lindleyin |  |

**Table S19**

Identificaiton of the top 10 components in E1.

| Ranking | Weights | No. | MS/ reference CAS | Component name | Structure/ Formula |
| --- | --- | --- | --- | --- | --- |
| 1 | 0.01966 | **42** | 419.134 [M-H]^-^,  259.098 [M-Glc+H]^+^ | Rhapontigenin 3'-*O*-glucoside |  |
| 2 | 0.01925 | **21** | 407.134 [M-H]^-^,  245.082 [M-H-Glc]^-^ | Torachrysone glucoside | C_20_H_24_O_9_ |
| 3 | 0.01770 | **35** | 660.104 [M]^-^ | - | - |
| 4 | 0.01484 | **37** | 731.161 [M+H]^+^,  729.145 [M-H]^-^ | Procyanidin B 3-*O*-gallate | C_37_H_30_O_16_ |
| 5 | 0.01435 | **34** | CAS# 128-57-4 | Sennoside B |  |
| 6 | 0.01431 | **46** | CAS# 81-27-6 | Sennoside A |  |
| 7 | 0.01316 | **11** | CAS# 154-23-4 | Cianidanol |  |
| 8 | 0.01307 | **98** | 219.065 [M+H]^+^ | chromone | C_12_H_10_O_4_ |
| 9 | 0.01290 | **36** | 463.123 [M+H]^+^ | 1,4-Dihydroxy-2-methoxy-3-  methyl-5-[3,4,5-trihydroxy-6-  (hydroxymethyl)oxan-2-yl]  oxyanthracene-9,10-dione |  |
| 10 | 0.01283 | **27** | 197.045 [M-H]^-^ | - | - |

**Table S20**

Identificaiton of the top 10 components in E2.

| Ranking | Weights | No. | MS/ reference CAS | Component name | Structure/ Formula |
| --- | --- | --- | --- | --- | --- |
| 1 | 0.01213 | **42** | 419.134 [M-H]^-^,  259.098 [M-Glc+H]^+^ | Rhapontigenin 3'-*O*-glucoside |  |
| 2 | 0.01178 | **7** | 291.087 [M+H]^+^, 289.071 [M-H]^-^ | Catechin | C_15_H_14_O_6_ |
| 3 | 0.01172 | **107** | 409.235 [M-H]^-^ | - | - |
| 4 | 0.01157 | **83** | 255.066 [M-Glc+H]^+^, 253.05 [M-Glc-H]^-^ | Chrysophanol glucoside | C_21_H_20_O_9_ |
| 5 | 0.01155 | **29** | 883.172 [M+H]^+^, 881.157 [M-H]^-^ | Procyanidin B 3,3'-di-*O*-gallate | C_44_H_34_O_20_ |
| 6 | 0.01145 | **28** | 547.115 [M+H]^+^ | Rhein-8-*O*-*β*-D-[6'-*O*-  (3''-methoxylmalonyl)]  glucopyranoside |  |
| 7 | 0.01144 | **61** | 883.172 [M+H]^+^, 881.157 [M-H]^-^ | Procyanidin B 3,3'-di-*O*-gallate | C_44_H_34_O_20_ |
| 8 | 0.01135 | **98** | 219.065 [M+H]^+^ | chromone | C_12_H_10_O_4_ |
| 9 | 0.01127 | **108** | 435.251 [M-H]^-^ | - | - |
| 10 | 0.01121 | **8** | 579.15 [M+H]^+^, 577.135 [M-H]^-^ | Procyanidin B | C_30_H_26_O_12_ |

**Table S21**

Identificaiton of the top 10 components in E3.

| Ranking | Weights | No. | MS/ reference CAS | Component name | Structure/ Formula |
| --- | --- | --- | --- | --- | --- |
| 1 | 0.01499 | **24** | CAS# 38963-95-0 | Resveratroloside |  |
| 2 | 0.01368 | **18** | CAS# 490-46-0 | (-)-Epicatechin |  |
| 3 | 0.01358 | **12** | 867.213 [M+H]^+^,  865.199 [M-H]^-^ | Flavanol trimer | C_45_H_38_O_18_ |
| 4 | 0.01341 | **86** | 255.066 [M-Glc+H]^+^, 253.05 [M-Glc-H]^-^ | Chrysophanol glucoside | C_21_H_20_O_9_ |
| 5 | 0.01329 | **72** | CAS# 4839-60-5 | Chrysophanol 1-*O*-*β*-D-glucoside |  |
| 6 | 0.01306 | **37** | 731.161 [M+H]^+^,  729.145 [M-H]^-^ | Procyanidin B 3-*O*-gallate | C_37_H_30_O_16_ |
| 7 | 0.01293 | **51** | 331.263 [M+H]^+^ | 2-Methyl-5-[pentadeca-  8,11-dienyl]benzene-1,3-diol |  |
| 8 | 0.01281 | **80** | 271.061 [M-Glc+H]^+^ | Emodin/ Aloe-emodin glucoside | C_21_H_20_O_10_ |
| 9 | 0.01258 | **26** | 477.103 [M-H]^-^ | 1-*O*-Galloyl *p*-coumaroylglucose | C_22_H_22_O_12_ |
| 10 | 0.01249 | **55** | 283.065 [M-Glc-H]^-^ | Rhein/ Physcion glucoside | C_22_H_22_O_10_ |

**Table S22**

Identificaiton of the top 10 components in E4.

| Ranking | Weights | No. | MS/ reference CAS | Component name | Structure/ Formula |
| --- | --- | --- | --- | --- | --- |
| 1 | 0.01538 | **40** | 419.173 [M+H]^+^ | 2-[4-[2-(3,5-Dimethoxyphenyl)  ethenyl]phenoxy]-6-(hydroxymethyl)  oxane-3,4,5-triol |  |
| 2 | 0.01458 | **81** | 247.097 [M-Glc+H]^+^,  245.081 [M-Glc-H]^-^ | Torachrysone glucoside | C_20_H_24_O_9_ |
| 3 | 0.01453 | **56** | 395.134 [M+H]^+^,  393.118 [M-H]^-^ | 2'-Oxoaloesol 7-glucoside |  |
| 4 | 0.01452 | **75** | 495.19 [M+H]^+^ | Raspberry ketone tetra-*O*-acetyl-*β*-D-glucopyranoside |  |
| 5 | 0.01410 | **69** | 451.124 [M-H]^-^ | (+)-Catechin glucopyranoside | C_21_H_24_O_11_ |
| 6 | 0.01368 | **53** | 270.053, 432.106 | Sennoside | C_42_H_40_O_19_ |
| 7 | 0.01341 | **9** | 483.077 [M-H]^-^ | Di-galloylglucose | C_20_H_20_O_14_ |
| 8 | 0.01333 | **64** | 253.05 [M-Glc-H]^-^ | Chrysophanol glucoside | C_21_H_20_O_9_ |
| 9 | 0.01287 | **100** | CAS# 518-82-1 | Emodin |  |
| 10 | 0.01256 | **58** | CAS# 38840-23-2 | Emodin 1-*O*-*β*-D-glucoside |  |

**Table S23**

Identificaiton of the top 10 components in E5.

| Ranking | Weights | No. | MS/ reference CAS | Component name | Structure/ Formula |
| --- | --- | --- | --- | --- | --- |
| 1 | 0.01486 | **19** | 451.124 [M-H]^-^ | (+)-Catechin glucopyranoside | C_21_H_24_O_11_ |
| 2 | 0.01448 | **55** | 283.065 [M-Glc-H]^-^ | Rhein/ Physcion glucoside | C_22_H_22_O_10_ |
| 3 | 0.01423 | **62** | 461.108 [M-H]^-^ | 1-*O*-Galloyl cinnamoylglucose | C_22_H_22_O_11_ |
| 4 | 0.01366 | **12** | 867.213 [M+H]^+^,  865.199 [M-H]^-^ | Flavanol trimer | C_45_H_38_O_18_ |
| 5 | 0.01267 | **33** | CAS# 59282-56-3 | Lindleyin |  |
| 6 | 0.01224 | **52** | 271.061 [M-Glc+H]^+^, 269.045 [M-Glc-H]^-^ | Emodin/ Aloe-emodin glucoside | C_21_H_20_O_10_ |
| 7 | 0.01199 | **22** | 431.098 [M-H]^-^ | Emodin/ Aloe-emodin glucoside | C_21_H_20_O_10_ |
| 8 | 0.01192 | **35** | 660.104 [M]^-^ | - | - |
| 9 | 0.01185 | **87** | 519.185 [M-H]^-^ | Isolindleyin trimethyl ether |  |
| 10 | 0.01182 | **64** | 253.05 [M-Glc-H]^-^ | Chrysophanol glucoside | C_21_H_20_O_9_ |

**
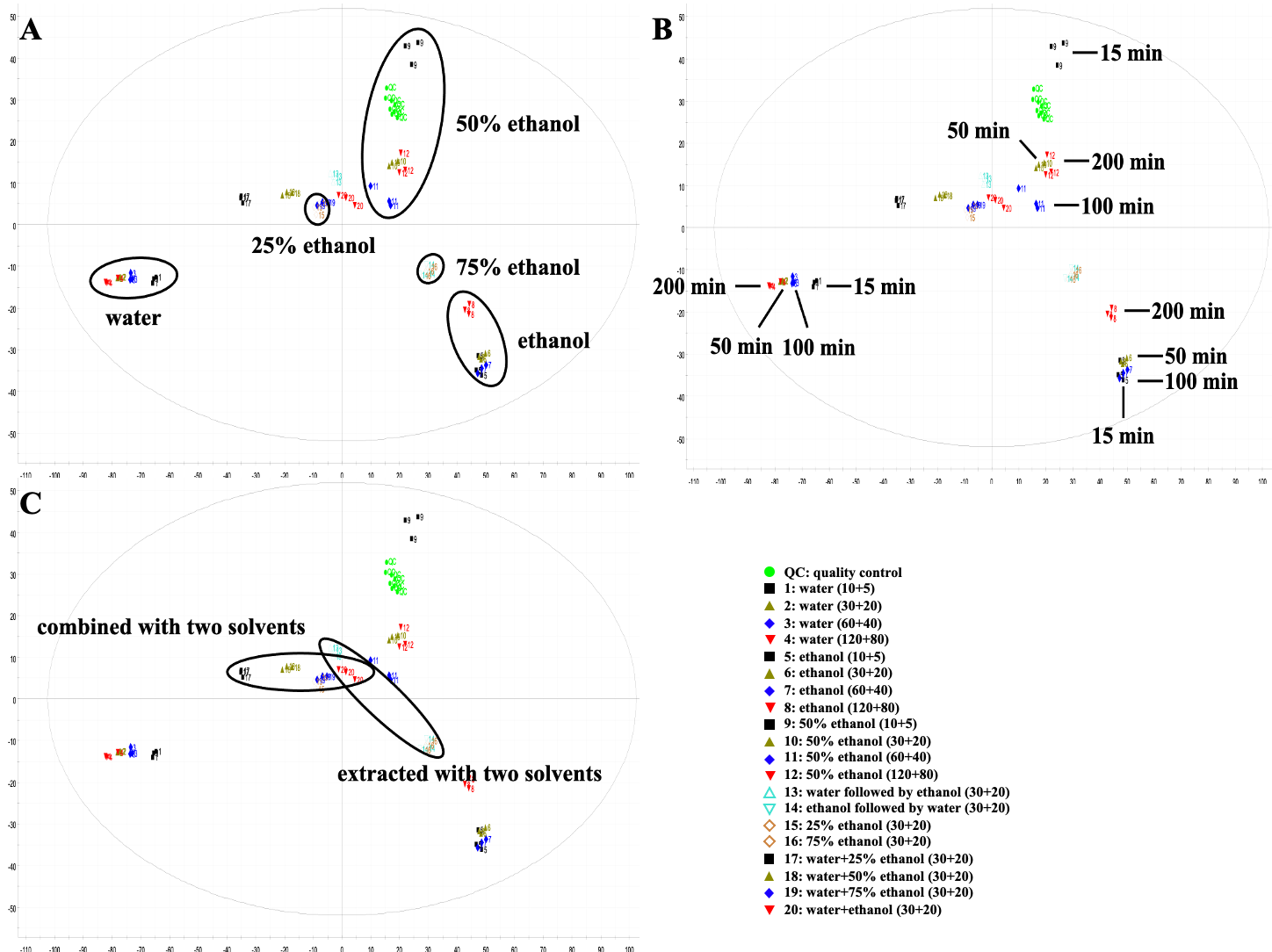
**

**Figure S1**

The LC-MS profiling related to different extraction methods: effect of extraction solvents (A), extraction time (B), extraction sequence and incorporation (C) on the difference of composition changes.

**
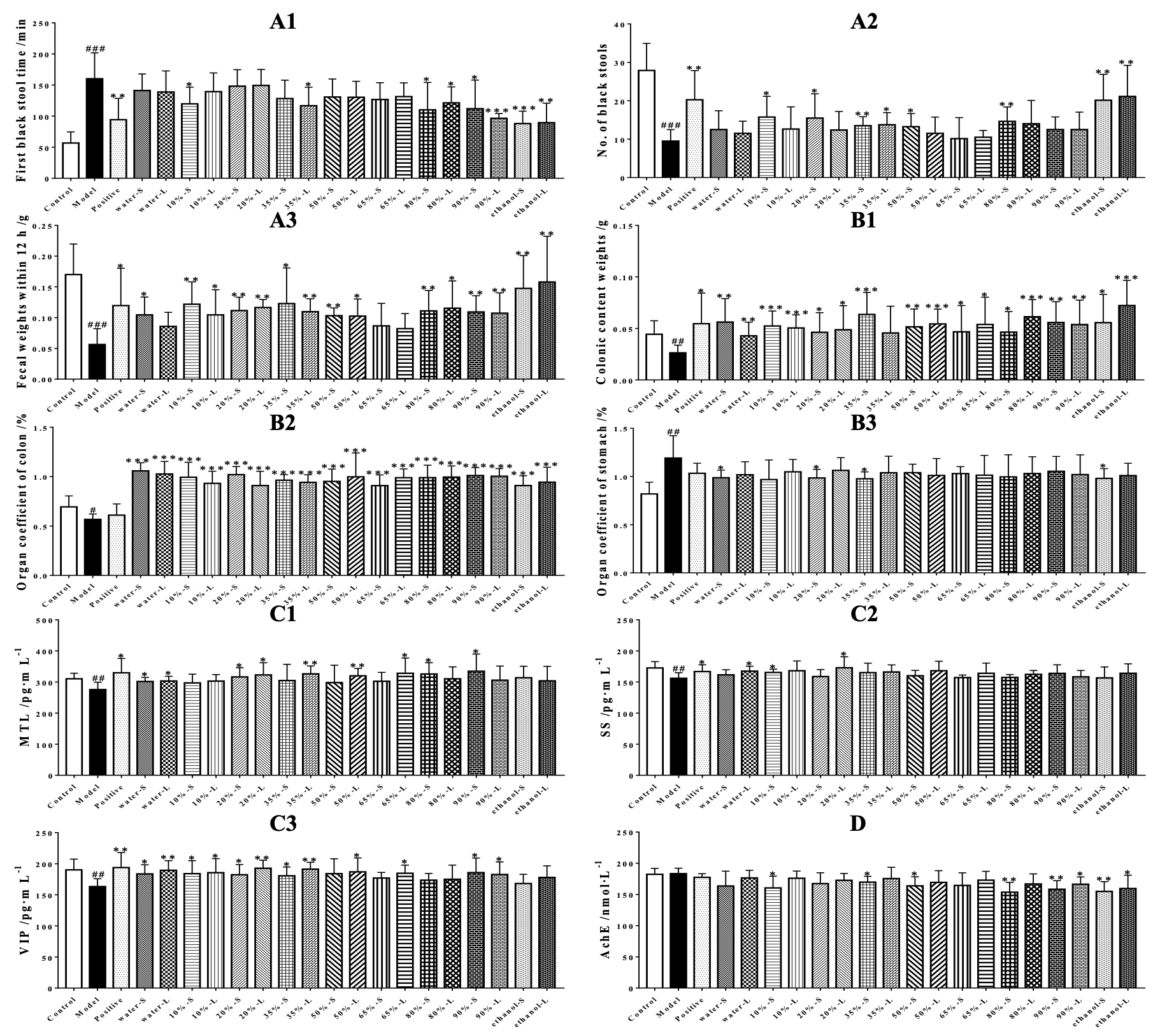
**

**Figure S2**

E1 indexes: defecation characteristics of first black stool time (A1), the number of black stools (A2), fecal weights within 12 h (A3); colonic content weights (B1), organ coefficients of colon (B2) and stomach (B3) after dissection; MTL (C1), SS (C2), VIP (C3) in mouse serum; AchE (D) in duodenal tissues. (*n* = 8, mean ± SD)

^#^ *P*<0.05, ^##^ *P*<0.01, ^###^ *P*<0.001 compared with the Control; * *P*<0.05, ** *P*<0.01, *** *P*<0.001 compared with the Model (the same figures below).

**
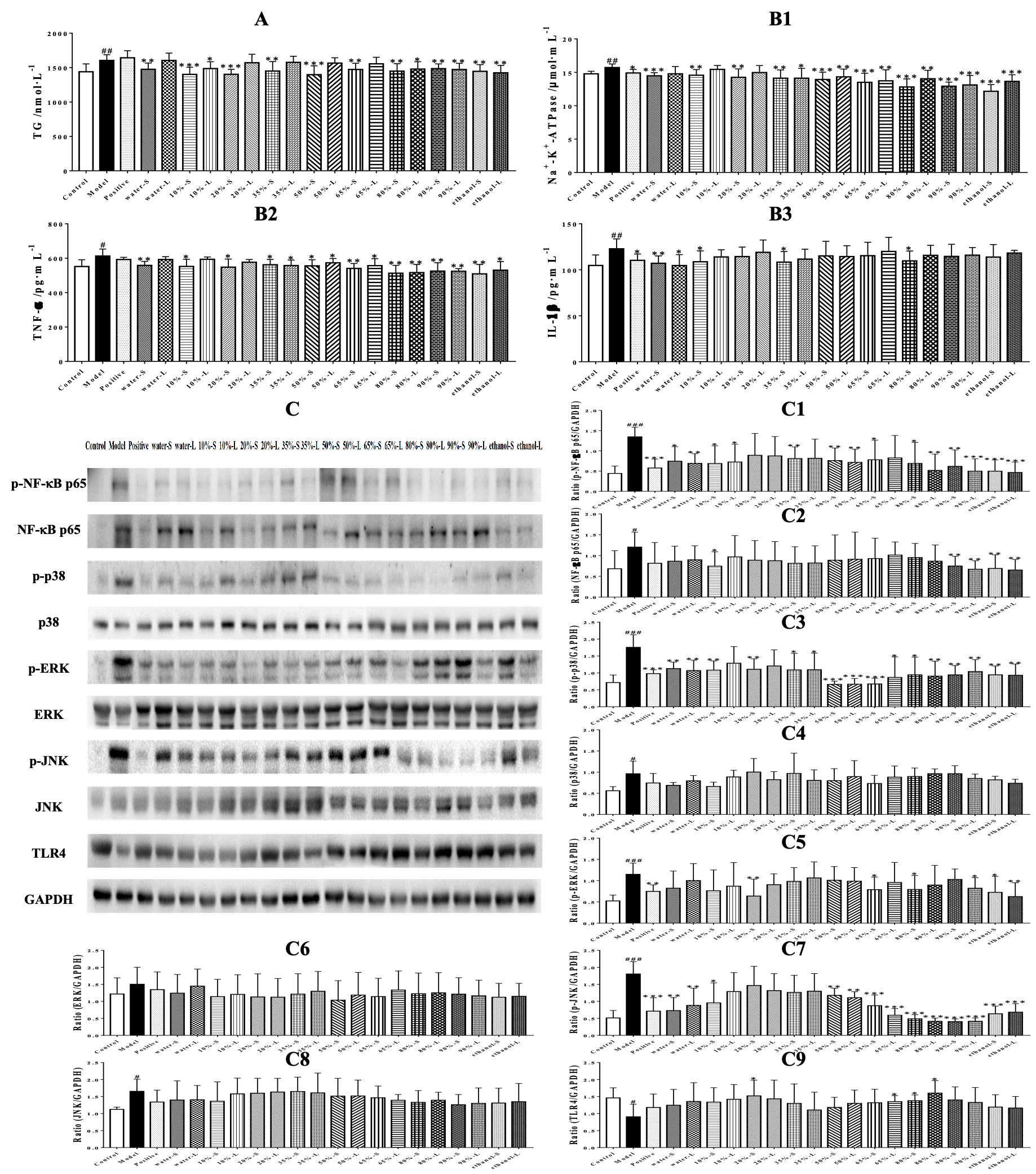
**

**Figure S3**

E2 indexes: TG (A) in mouse serum; Na^+^-K^+^-ATPase (B1), TNF-α (B2), IL-1β (B3) in duodenal tissues; representative exposed protein bands (C) and corresponding grayscale ratios of p-NF-κB p65 (C1), NF-κB p65 (C2), p-p38 (C3), p38 (C4), p-ERK (C5), ERK (C6), p-JNK (C7), JNK (C8), TLR4 (C9) in the colon of each group. (*n* = 8, mean ± SD)

**
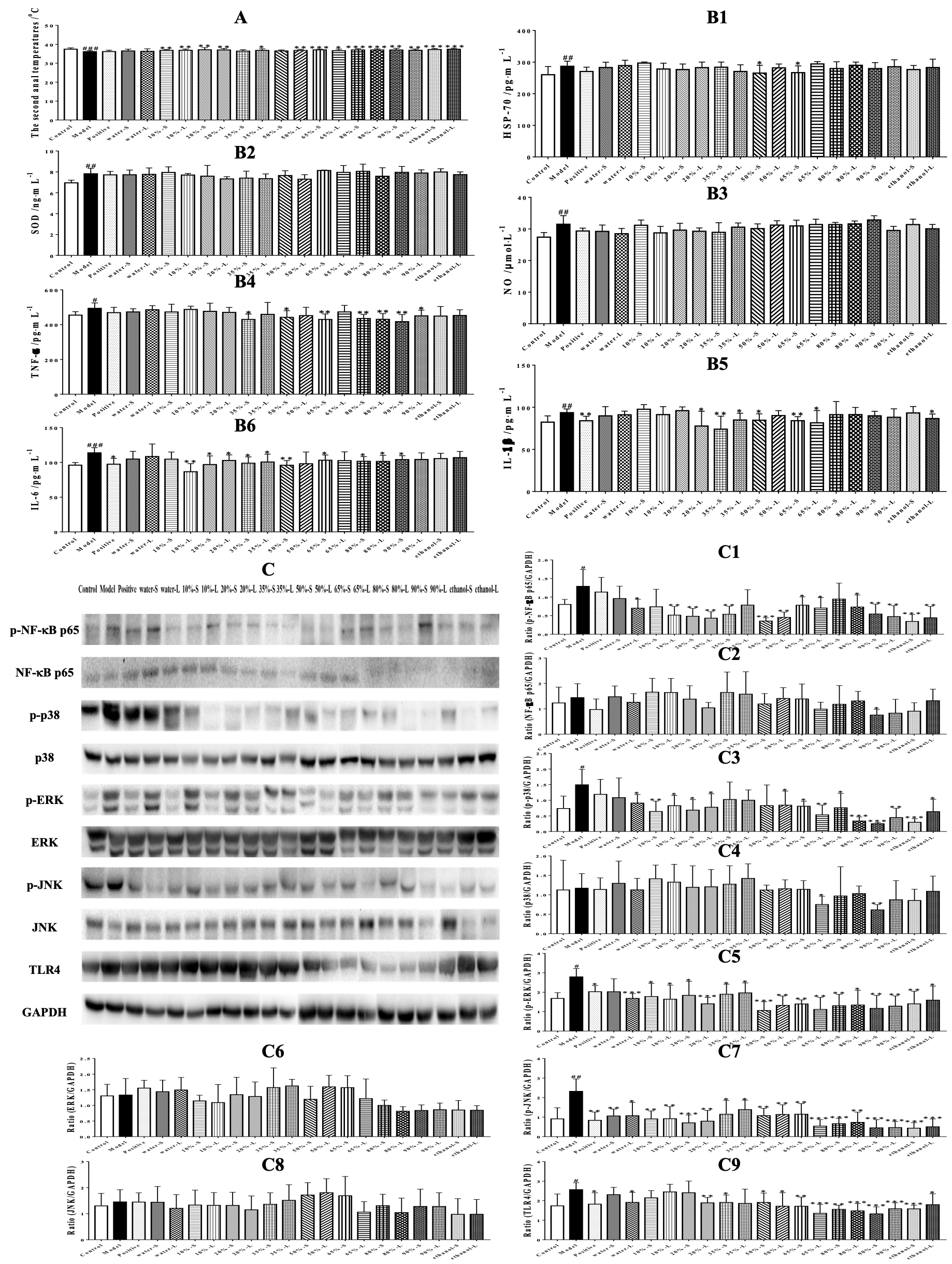
**

**Figure S4**

E3 indexes: the second measurement of anal temperatures (A); HSP-70 (B1), SOD (B2), NO (B3), TNF-α (B4), IL-1β (B5), IL-6 (B6) in mouse serum; representative exposed protein bands (C) and corresponding grayscale ratios of p-NF-κB p65 (C1), NF-κB p65 (C2), p-p38 (C3), p38 (C4), p-ERK (C5), ERK (C6), p-JNK (C7), JNK (C8), TLR4 (C9) in the colon of each group. (*n* = 6, mean ± SD)

**
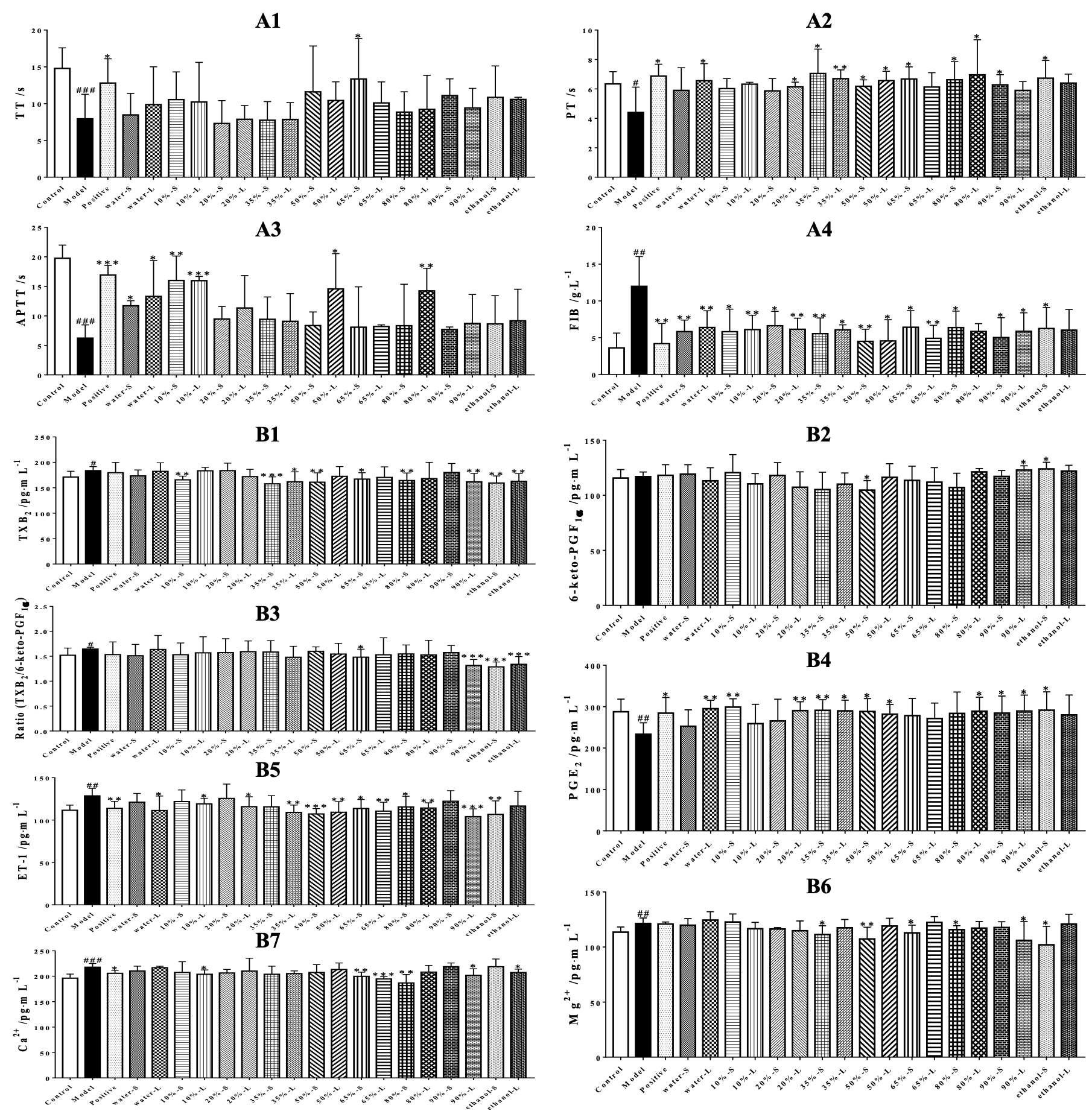
**

**Figure S5**

E4 indexes: TT (A1), PT (A2), APTT (A3), FIB (A4) in mouse plasma; TXB_2_ (B1), 6-keto-PGF_1α_ (B2), ratios of TXB_2_ to 6-keto-PGF_1α_ (B3), PGE_2_ (B4), ET-1 (B5), Mg^2+^ (B6), Ca^2+^ (B7) in the serum. (*n* = 6, mean ± SD)

**
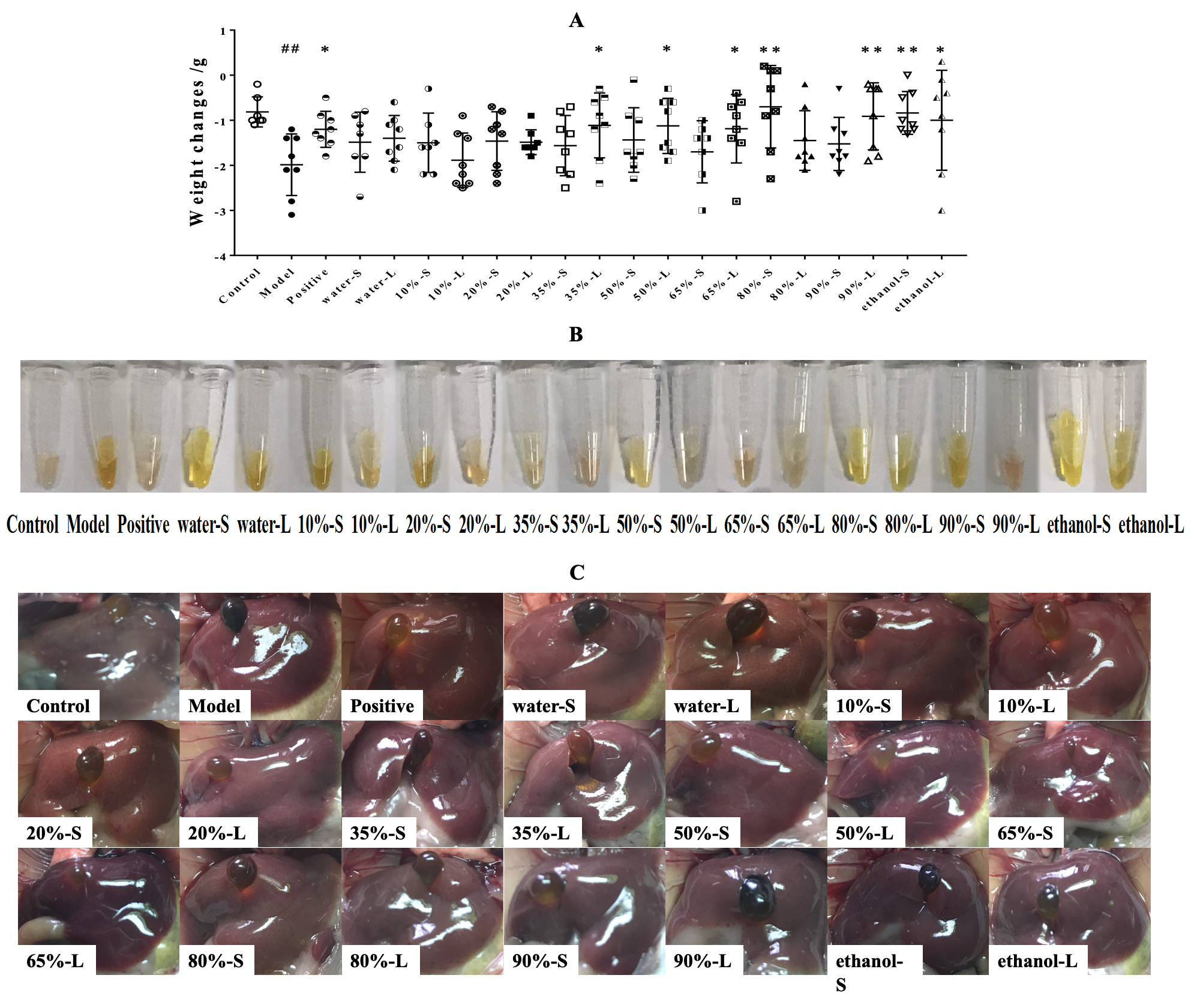
**

**Figure S6**

Cholestatic manifestations of weight changes (A) before and after ANIT modeling, representative serum color (B), livers and gallbladders (C) after morphological dissection in each mouse group. (*n* = 8, mean ± SD)

**
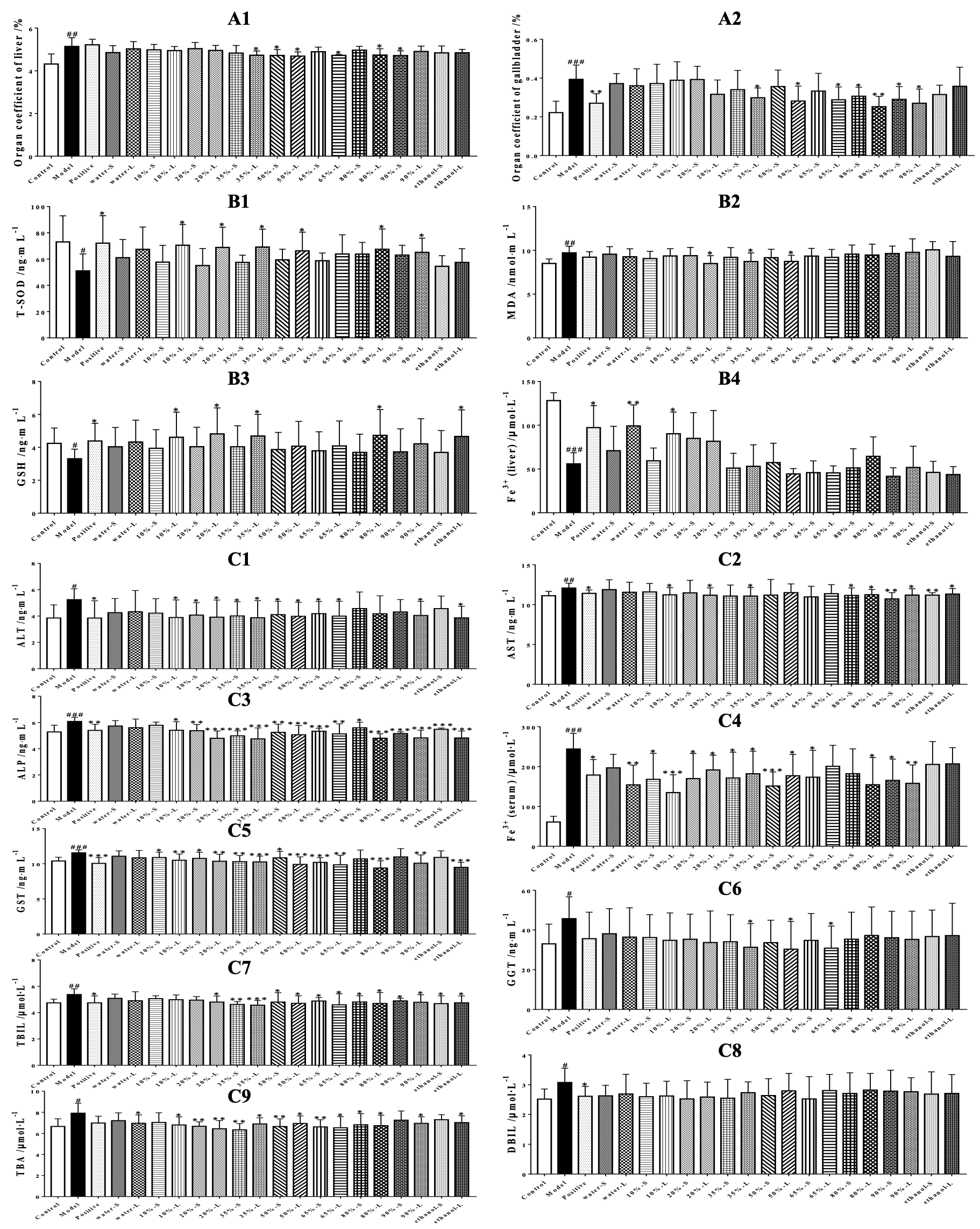
**

**Figure S7**

E5 indexes: organ coefficients of liver (A1) and gallbladder (A2); T-SOD (B1), MDA (B2), GSH (B3), Fe^3+^ (B4) in the homogenate of liver tissues; ALT (C1), AST (C2), ALP (C3), Fe^3+^ (C4), GST (C5), GGT (C6), TBIL (C7), DBIL (C8), TBA (C9) in mouse serum. (*n* = 8, mean ± SD)

**
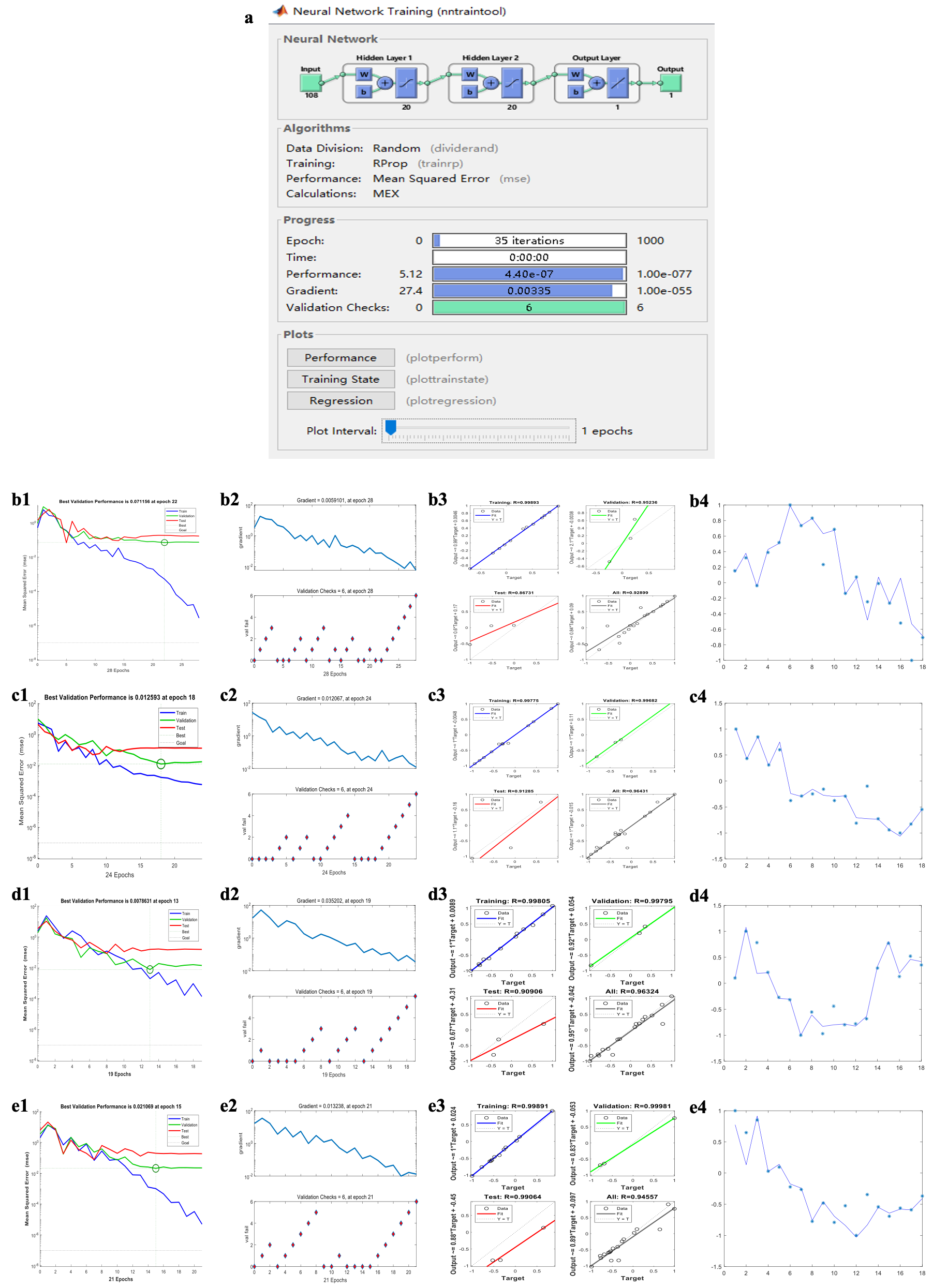
**

**Figure S8**

The running program of BP neural network (a) including four parts: the network structure, algorithms, training progress and plots; running effect diagrams of E2 (b), E3 (c), E4 (d) and E5 (e) including the neural network performance (1), training state (2), regression analysis (3), comparison between the predicted and true values (4: the line is the prediction curve and “*” denotes the true value of each group).
